# Anticancer Activity of Half-Sandwich Ru, Rh and Ir Complexes with Chrysin Derived Ligands: Strong Effect of the Side Chain in the Ligand and Influence of the Metal

**DOI:** 10.3390/pharmaceutics13101540

**Published:** 2021-09-23

**Authors:** Ana R. Rubio, Rocío González, Natalia Busto, Mónica Vaquero, Ana L. Iglesias, Félix A. Jalón, Gustavo Espino, Ana M. Rodríguez, Begoña García, Blanca R. Manzano

**Affiliations:** 1 Departamento de Química, Facultad de Ciencias, Universidad de Burgos, Plaza Misael Bañuelos s/n, 09001 Burgos, Spain; ana.rosa.rubio.antolin@gmail.com (A.R.R.); mvaquero@ubu.es (M.V.); gespino@ubu.es (G.E.); begar@ubu.es (B.G.); 2 Facultad de Ciencias y Tecnologías Químicas-IRICA, Universidad de Castilla-La Mancha, Avda. C. J. Cela 10, 13071 Ciudad Real, Spain; dra.gonzalez.alvarez@gmail.com (R.G.); aiglesias@uabc.edu.mx (A.L.I.); Felix.Jalon@uclm.es (F.A.J.); 3 Facultad de Ciencias de la Ingeniería y Tecnología (FCITEC), Universidad Autónoma de Baja California, Blvd. Universitario # 1000, Unidad Valle de las Palmas, Baja California, Tijuana 21500, Mexico; 4 Departamento de Química Inorgánica, Orgánica y Bioquímica, Escuela Técnica Superior de Ingenieros Industriales, Universidad de Castilla-La Mancha, Avda. C. J. Cela 2, 13071 Ciudad Real, Spain; AnaMaria.RFdez@uclm.es

**Keywords:** chrysin ligands, iridium, ruthenium, rhodium, cancer, metallodrugs, piperidine, half-sandwich

## Abstract

An important challenge in the field of anticancer chemotherapy is the search for new species to overcome the resistance of standard drugs. An interesting approach is to link bioactive ligands to metal fragments. In this work, we have synthesized a set of *p*-cymene-Ru or cyclopentadienyl-M (M = Rh, Ir) complexes with four chrysin-derived pro-ligands with different -OR substituents at position 7 of ring A. The introduction of a piperidine ring on chrysin led to the highly cytotoxic pro-ligand **HL4** and its metal complexes **L4-M** (SW480 and A549 cell lines, cytotoxic order: **L4-Ir** > **L4-Ru** ≈ **L4-Rh**). **HL4** and its complexes induce apoptosis and can overcome cis-platinum resistance. However, **HL4** turns out to be more cytotoxic in healthy than in tumor cells in contrast to its metal complexes which displayed higher selectivity than cisplatin towards cancer cells. All **L4-M** complexes interact with double stranded DNA. Nonetheless, the influence of the metal is clear because only complex **L4-Ir** causes DNA cleavage, through the generation of highly reactive oxygen species (^1^O_2_). This result supports the hypothesis of a potential dual mechanism consisting of two different chemical pathways: DNA binding and ROS generation. This behavior provides this complex with a great effectivity in terms of cytotoxicity.

## 1. Introduction

**Figure 1:**
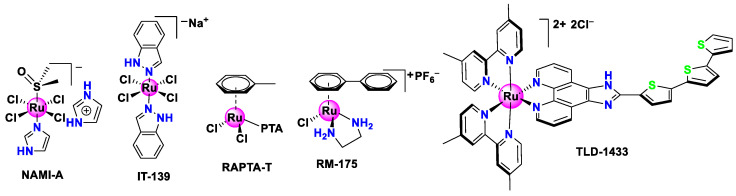
Examples of ruthenium complexes with clinical potential.

The acquired or intrinsic resistance of standard drugs is a major challenge in cancer treatment [1]. In order to overcome this drawback, new kinds of anticancer agents are the focus of current research as an alternative to the classical organic or platinum-based drugs [2,3]. During recent decades, organometallic anticancer agents have emerged [4,5,6,7] as potential pharmaceutical options. More specifically, ruthenium compounds are considered the most promising drug candidates to date, because of their favorable kinetic properties, rich redox chemistry and high selectivity for cancer cells [8,9,10,11,12,13,14,15,16,17,18,19,20,21,22]. In fact, several ruthenium complexes, such as the octahedral NAMI-A [23] or sodium *trans*-tetra-chloridobis(1H-indazole)ruthenate(III) (IT-139) [15,24,25,26] complexes as well as arene-Ru(II) derivatives of the RAPTA family [27,28,29,30], containing the PTA ligand, or RM175 [31,32], have been evaluated as chemotherapeutic agents in clinical trials (see Figure 1). Unfortunately, during a phase I/II study, the clinical activity of NAMI-A was found to be disappointing [33]. Besides, the octahedral Ru complex TLD-1433 is the first Ru(II)-based photosensitizer for photodynamic therapy (PDT) to enter a human clinical trial [34].

Iridium or rhodium complexes have also become the focus of research due to their antiproliferative activity, efficient cellular uptake, and, in some cases, positive tolerance by healthy cells [35,36,37,38]. Moreover, half-sandwich organometallic low-spin d^6^ metal complexes as Ru-arene or Rh- or Ir-cyclopentadienyl derivatives have attracted great interest as anticancer agents because of the hydrophobic nature of the cyclopentadienyl or arene fragments that can be modulated. These entities can influence the cellular uptake [39,40,41] and complex stability [42]. Besides, the pharmacological properties of these derivatives can be easily modulated by variation not only of these entities but also of the chelating or monodentate ancillary ligands and the leaving groups [43]. Considering these premises, it is necessary to design innovative complexes that could overcome the limitations of existing drugs.

An interesting approach to address the problem of the resistance in the field of metallic chemotherapeutic agents is to use multi-targeted drugs. This may be achieved by linking bioactive ligands to the metal fragments [10,22,44,45]. The compounds thus obtained offer additional advantages over classic anti-cancer agents, such as improved pharmacological properties, tunable antitumor activity, intramolecular combination therapy and selective targeted properties [46]. In this context, flavonoids are a group of naturally occurring plant phenolic compounds that exhibit interesting biological properties including antioxidant behavior [47,48,49], which depends mostly on the number and position of the hydroxyl groups in their structure, but also anti-inflammatory, estrogenic, antimicrobial and anticancer activity [47,50,51,52]. Flavonoids act as enzyme inhibitors [53], signaling molecules [54], DNA intercalators [52,55] and chelators of metal ions [56]. Some enzymes targeted by flavone derivatives are important for the growth of cancer cells, a fact that reinforces the design concept of metal-flavone complexes as multitargeted compounds [57]. Amidst flavonoids, quercetin and its metal complexes have been studied in terms of DNA binding studies, in order to establish structure-activity relationships [58]. Recently, Ru [59,60,61,62,63,64,65,66], V [67,68], Zn [69], Cu [70,71], Fe [72] and La [73] complexes bearing bioactive flavonoids or related ligands have been described. This also include half-sandwich ruthenium [74,75,76,77], rhodium [74,75] and osmium [75,78] derivatives that exhibit cytotoxic activity. An excellent review of the biological properties of Ru complexes with flavonoids ligands has recently appeared [57]. Research on the synthesis of iridium complexes with flavonoids is very scarce; only a flavonol derivative of this metal, active as antioxidant, has been recently reported [79]. The anticancer activity of different flavonoid-containing metal complexes of Ni, Zn, Se, Mn and Cu has been reported [71,80]. In the case of Ru(II)-*p*-cymene derivatives [76] the role played by the ligand system has been evaluated. However, the effect of the metal has been barely analyzed [75].

The natural flavone chrysin (5,7-dihydroxy-2-phenyl-4Hchromen-4-one) exhibits interesting pharmacological properties. It reduces oxidative stress in neuronal tissues, neurotoxicity, neuro-inflammation, cognitive dysfunctions [81,82,83] and also has potential as anticancer agent [84]. To the best of our knowledge, only some recent reports of octahedral Ru complexes with deprotonated chrysin (chrys) and N^N [85,86], chloride [87] or trithiacyclononane [60] as accompanying ligands have been reported. Some of these compounds bind CT DNA and induce apoptosis in cancer cells or additionally have a strong affinity to BSA (bovine serum albumin). With regard to arene complexes, the derivative [(*p*-cym)RuCl(chrys)] has been shown to modulate platelet activity [88] and similar derivatives with organocalcogen (S,Se) side chains in position 7 of the chrysin structure, exhibited chemotherapeutic activity against breast cancer cells [89]. However, the synthesis and study of the biological properties of half-sandwich Rh and/or Ir complexes with chrysin or chrysin derived ligands have not been addressed.

**Figure 2:**
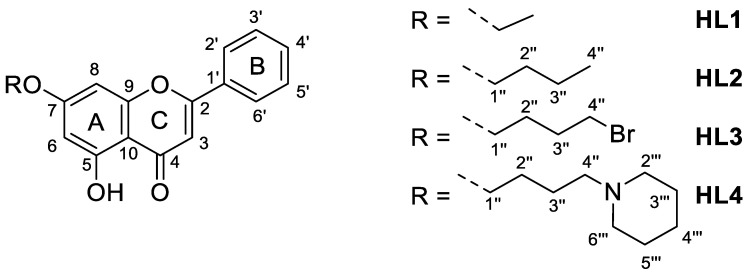
Molecular structure of ligand precursors (**HL1**–**HL4**) and atom numbering scheme.

In this work, we functionalized the -OH group at position 7 of chrysin with alkyl chains with or without terminal groups (Br, piperidine), in order to modulate their biological properties (see Figure 2). The introduction of the piperidine moiety was based on previous positive effects in the cytotoxic activity after the introduction of this group in chrysin or related derivatives [90,91]. After deprotonation of the chrysin compounds, the anionic species will be able to act as chelators for the metal centers and, in fact, they coordinate to *p*-cymene-Ru, Cp*-Rh and Cp*-Ir fragments to afford the corresponding half-sandwich complexes. In this manner, potential multi-targeted compounds will be obtained. Furthermore, the coordination will eliminate the intramolecular hydrogen bond between the C=O group on the 5-OH position, a bond that has been proposed to produce low bioavailability [90]. The aim of this work was to carry out a SAR study evaluating the influence not only of the substitution on the ligand but also that of the metal center in their biological properties. We will show that the most cytotoxic species are those containing the ligand **L4** and the most promising one is that with iridium as the metal center. The targets were determined, and the results of cytotoxicity-cellular uptake were also examined.

## 2. Materials and Methods

All reactions, except where noted, were carried out under an atmosphere of dry oxygen-free nitrogen using standard Schlenk techniques. Solvents were distilled under inert atmosphere from the appropriate drying agents and degassed before use or purified in a MBraun SPS MB-SPS-800 from commercial HPLC solvents. Piperidine was distilled prior to use. CDCl_3_ was deoxygenated and distilled. Reactions requiring heat were performed with thermostated oil baths. Elemental analyses were performed with a Thermo Quest FlashEA 1112 microanalyser and IR spectra on a Shimadzu IR Prestige-21 infrared spectrometer equipped with a Pike Technologies ATR. The FAB^+^ mass spectrometry measurements were obtained with a Thermo MAT95XP mass spectrophotometer with a magnetic sector. NMR spectra were recorded on Varian Innova 500 and Varian Unity 400. ^1^H and ^13^C{^1^H} NMR chemical shifts are reported in ppm referenced to TMS. For ^1^H−^13^C g−HMQC spectra, the standard Varian pulse sequences were used (VNMR 6.1 C software). The spectra were acquired using 16096 Hz (^1^H) and 25133.5 Hz (^13^C) widths; 16 transients of 2048 data points were collected for each of the 256 increments. In the NMR analysis s, d, t, q, qt, sex, sept, m and bs denote singlet, doublet, triplet, quartet, quintuplet, sextuplet, septuplet, multiplet and broad signal, respectively. Unless otherwise stated, the ^13^C{^1^H} NMR signals are singlets. The proligands **HL1**-**HL2** [92,93], **HL3** [47,94,95,96,97,98] and **HL4** [47,95] and the metal precursors [Ru(*ŋ*^6^-*p*-cym)Cl_2_]_2_ (*p*-cym = *p*-cymene) [99,100] or [M(*ŋ*^5^-Cp*)Cl_2_]_2_ (M = Rh, Ir, Cp* = pentamethylcyclopentadienyl) [101] were prepared by published procedures.

Information regarding the synthesis and characterization of the 12 new Ru, Rh and Ir complexes is gathered in the Supplementary Materials.

### 2.1. Crystal Data for **HL2**

Suitable crystals of **HL2** were grown from a toluene/pentane mixture. Intensity data were collected at 290 K on a Bruker X8 APEX II CCD-based diffractometer, equipped with a graphite monochromated MoKα radiation source (λ = 0.71073 Å). Data were integrated using SAINT [102] and an absorption correction was performed with the program SADABS [103]. The structure was solved by direct methods using WINGX package [104,105], and refined by full-matrix least-squares methods based on *F*^2^. Hydrogen atoms were placed using a “riding model” and included in the refinement at calculated positions. The CCDC deposition number is 2019578.

### 2.2. General Procedure for Stability Studies by NMR Spectroscopy

For the stability in DMSO-*d*_6_, the complexes **L4-Ru**, **L4-Rh**, and **L4-Ir** (5 mg) were dissolved in 300 µL DMSO-*d*_6_ (approx. 2.1 × 10^−2^ M), and ^1^H NMR spectra were recorded during 24 h in a 400 MHz spectrometer.

For the aquation studies, 200 μL of D_2_O (approx. 1.5 × 10^−2^ M) was added over the previous solution, and ^1^H NMR spectra were recorded during 24 h in a 400 MHz spectrometer.

### 2.3. Cell Lines Culture

Human colon cancer (SW480), lung adenocarcinoma (A549) and lung fibroblasts (IMR90) cells from the ECACC (European Collection of Authenticated Cell Cultures, Salisbury, UK) were cultured in Dulbecco’s Modified Eagle’s Medium (DMEM) supplemented with 10% fetal bovine serum (FBS) and 1% amphotericin-penicillin-streptomycin solution. Ovarian cancer (A2780) and cisplatin resistant ovarian cancer cells (A2780cis) cells were cultured in RPMI-1640 supplemented with 2 mM of Glutamine, 10% fetal bovine serum (FBS) and 1% amphotericin-penicillin-streptomycin solution. Cells were incubated at 37 °C under a humidified 5% CO_2_ atmosphere.

### 2.4. Antiproliferative Activity

Antiproliferative activity was evaluated by the MTT Assay as previously described [106]. Briefly, cells were seeded in 96-well plates at a density of 5 × 10^3^ (IMR-90 and A549) or 1 × 10^4^ (SW480, A2780 and A2780cis) and incubated for 24 h. After attachment to the surface, the cells were incubated with various concentrations of the studied complexes, freshly dissolved in DMSO and diluted in the culture medium. Then, 100 µL of 500 µg/mL of thiazolyl blue tetrazolium bromide (MTT) was added to each well. After 4 h, the formazan product was dissolved by adding 100 µL of 10% sodium dodecyl sulfate (SDS) in 0.01 M HCl and incubated overnight. Absorbance was read at 590 nm in a microplate reader. Cisplatin was also included as a positive control. Half maximal inhibitory concentration (IC_50_) values, expressed as percentage values respect to the controls, were calculated using the GrahPad Prism 6.01 analysis software from three independent experiments.

### 2.5. Cellular Uptake Studies

To study cellular uptake by inductively coupled plasma-mass spectrometry (ICP-MS), 1.5 × 10^5^ A549 cells were seeded in 2 mL DMEM culture medium in 6-well plates for 24 h at 37 °C and 5% CO_2_. Then cells were treated at a determined concentration for 24 h and they were harvested, centrifuged and re-suspended in phosphate buffered saline (PBS). Finally, samples were digested with 65% HNO_3_ at room temperature for 24 h and diluted with Mili-Q water to obtain final 2% HNO_3_ solutions before being analyzed by ICP-MS.

### 2.6. Intracellular ROS Generation

A549 cells were seeded in 12-well plates at a density of 4 × 10^4^ cells/well and incubated for 24 h at 37 °C and 5% CO_2_. Then, cells were incubated for 30 min with 25 µM of H_2_DCFDA in DMEM medium without phenol red and 1% FBS. Then, cells were washed twice with DPBS and incubated with the compounds under study in complete medium at their half maximal inhibitory concentration for other 4 h. A total of 20 µM of TBH were included as positive control. After treatment, cells were harvested and washed with DPBS. Data were collected in a NovoCyte Flow cytometer (ACEA Biosciences, Inc., Diego, CA, USA). A total of 10,000 events per sample were counted and analyzed by NovoExpress 1.4.0 Software. Three replicates and two independent experiments were performed.

### 2.7. Apoptosis Detection

Apoptosis was evaluated by an Annexin V:FITC Assay Kit (Biorad, Hercules, CA, USA) according to the manufacturer’s instructions. Data were collected in a NovoCyte Flow cytometer (ACEA Biosciences, Inc., Diego, CA, USA). A total of 10,000 events were counted and analyzed by NovoExpress 1.4.0 Software. Two replicates and two independent experiments were performed.

### 2.8. Cell Cycle Arrest

A total of 1.5 × 10^5^ A549 cells per well were seeded in 6-well plates and incubated for 24 h. Then, cells were treated at the IC_50_ value for each compound. Cells without any treatment and vehicle-treated cells (0.5% DMSO) were used as control. After 24 h of treatment, cells were harvested and centrifuged, resuspended in cold PBS and fixed in 70% EtOH overnight at 4 °C. Then, samples were treated with 0.1 mg/mL PI, 0.1 mM EDTA and 0.1% Triton-×100 in PBS to permeabilize and stain cells, and with RNAse (2 mg/mL) for 30 min in ice and protected from light. Finally, cells were analyzed using the NovoCyte Flow cytometer (ACEA Biosciences, Inc., Diego, CA, USA). Cell cycle distribution was evaluated by NovoExpress 1.4.0 Software. Two replicates were included, and two independent experiments were performed.

### 2.9. Statistical Analysis

All the results were expressed as the mean with the standard deviation. Statistical analysis was performed by ANOVA with Dunnet’s Test for multiple comparison with the software GraphPad Prism 6.

### 2.10. UV-Vis Spectroscopy

Absorbance spectra were performed on a Hewlett-Packard 8453A (Agilent Technologies, Santa Clara, CA, USA) spectrophotometer fitted out with a Peltier temperature control system at T = 25 °C in 1.0 cm path-length cells. Substitution or binding kinetics were measured analyzing the absorbance change of the complexes solutions recorded over time. The melting experiments were carried out using a DNA solution in three C_D_/C_P_ ratios: 0, 0.05 and 0.5, recording the absorbance spectra while heating from 30 to 90 °C at 0.3 °C/min.

### 2.11. Circular Dichroism Spectroscopy

CD measurements were performed with a MOS-450 biological spectrophotometer (Bio-Logic SAS, Claix, France) fitted out with 1.0 cm path length cells. Samples were prepared incubating DNA with flavonoid complexes overnight in 2.5 mM sodium cacodylate (NaCaC) buffer at pH = 7.0 and T = 25 °C. Spectrograms were obtained in the 200−600 nm range at 2 nm/s speed. Molar ellipticity (Deg M^−1^ cm^−1^) was calculated using [θ] = 100·θ/C_P_·l, where C_P_ is the polynucleotide concentration and l is the cell light path (cm).

### 2.12. Viscosity Measurements

A Micro-Ubbelohde viscometer whose temperature was controlled externally (25 ± 0.1 °C) was used for viscosity measurements. The flow time was measured with a digital stopwatch. Mean values of triplicated measurements were taken to evaluate the DNA viscosity in the absence (η_0_) and in the presence, (η) of iridium complexes. The viscosity readings were expressed as L/L_0_ = (η/η_0_)^1/3^ versus C_D_/C_P_ ratio.

### 2.13. Native Polyacrylamide Gel Electrophoresis (PAGE)

Native PAGE was performed as previously described [106]. Briefly, 1.5 µM of BSA was incubated with different complex concentrations in Tris HCl buffer (0.5 M, pH = 6.8. Then samples were loaded in 10% polyacrylamide gel BSA, which was immersed in tris borate and EDTA (TBE) 1X buffer. The gel was run at 6.6 V/cm for 4 h at 4 °C and then stained with Coomassie Brilliant Blue R-250. Finally, the gel was visualized with a Bio-Rad Gel Doc XR+ Imaging System.

### 2.14. Plasmidic Electrophoresis Assays

Agarose gel electrophoresis of plasmid DNA pUC18 was performed using 1% agarose gel in TBE buffer and ethidium bromide as staining agent. The samples were prepared by mixing 23.8 μM of pUC18 plasmid with different concentration of the studied compounds being the final [complex]/[plasmid] ratios of 5, 10 and 20. The samples were incubated overnight. Afterwards, 2 μL of loading buffer (Bio-Rad, Glycerol 25%) were added for a 12 µL final volume. After homogenization, electrophoresis was run at 5 V/cm during 1 h and the gel was visualized with a Bio-Rad Gel Doc XR+ Imaging System. Another electrophoresis was carried out in the same way incubating the samples with ROS scavengers.

## 3. Results and Discussion

### 3.1. Synthesis and Characterization of the Ligands and Complexes

**Scheme 1:**
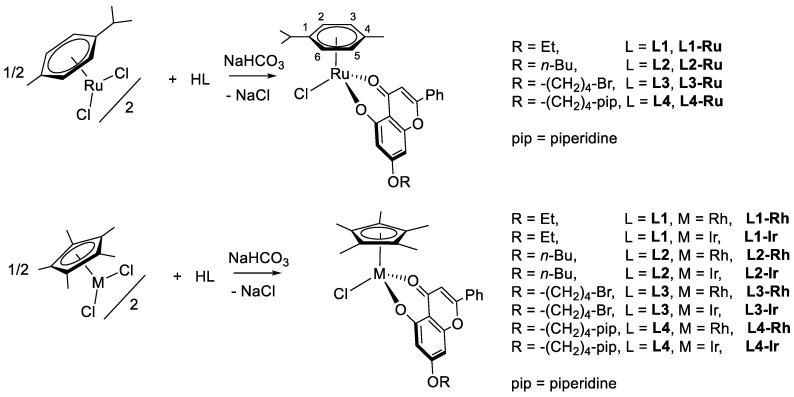
Synthesis and molecular structure of the new Ru, Rh and Ir complexes containing chrysin derived ligands.

The proligands **HL1**, **HL2** [92,93] and **HL3** [47,94,95,96,97,98] were obtained from the treatment of chrysin with K_2_CO_3_ and the corresponding bromo or dibromo alkanes. Reaction of **HL3** with piperidine and K_2_CO_3_ yielded **HL4** [47,95]. The proligands were then deprotonated with NaHCO_3_ and the obtained **L1**–**L4** anionic species were made to react with [Ru(η^6^-*p*-cym)Cl_2_]_2_ (*p*-cym = *p*-cymene) or [M(η^5^-Cp*)Cl_2_]_2_ (M = Rh, Ir,; Cp* = pentamethylcyclopentadienyl) dimers, to afford 12 new complexes of general formula [LMCl(O^O)], where L = *p*-cym, Cp* (see Scheme 1).

The proligands **HL1**–**HL4** and the complexes were fully characterized by elemental analysis, mass spectrometry, IR and ^1^H and ^13^C{^1^H} NMR spectroscopy. The assignment of the different resonances was based on the literature data [60,95,98,101,102,103,104,105,106,107,108] and 2D NMR experiments.

In the IR spectra of **HL1**–**HL4**, the ν(C=O) vibration appears in the range 1651–1661 cm^−1^, while for the respective complexes the band is displaced to lower wavenumbers (1627–1636 cm^−1^), a fact that reflects the involvement of the C=O group in the coordination to the metal centers [109].

The ^1^H NMR spectra of **HL1**–**HL4** displayed a broad singlet at 12.61–12.70 ppm for the phenolic hydroxyl group. The low field of these resonances suggests its participation in intramolecular hydrogen bonding interactions (observed in the solved X-ray diffraction structure for **HL2**, *vide infra*, or that of **HL3**) [97]. As expected, this resonance disappears upon complex formation. The position of the resonances of ligands and complexes are reflected in Table S1 of SI. No significant differences in the chemical shifts of protons 3′, 4′ and 5′ of ring B of the **L4-M** complexes were observed and small shifts were detected for protons of A and C rings [95,98,108]. In the case of the ruthenium complexes, the lack of symmetry leads to the observation of two different isopropylic methyl groups for the *p*-cymene fragment (see ^1^H and ^13^C{^1^H}NMR spectra and characterization in SI). Interestingly, four aromatic CH groups were observed in the ^13^C{^1^H}NMR spectra, although only two proton resonances for these groups were observed in each complex.

**Figure 3:**
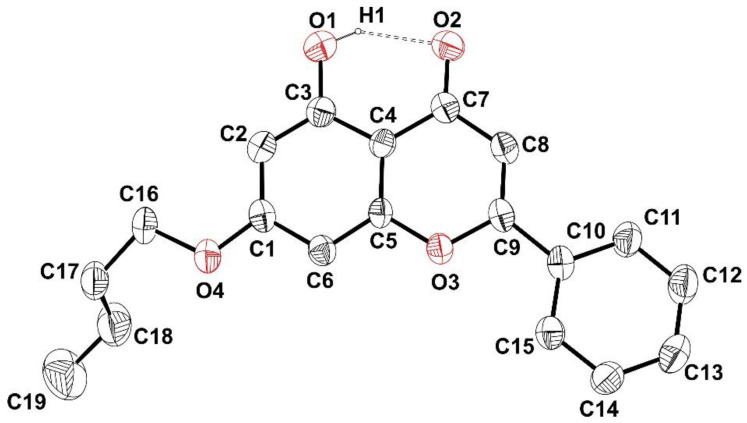
ORTEP representation of **HL2** (ellipsoids at the 30% probability). Hydrogen atoms have been omitted for clarity.

Unsuccessful attempts of crystallization of the metal complexes were performed. Nevertheless, the unknown structure of proligand **HL2** was solved by single crystal X-Ray diffraction. The corresponding ORTEP representation is shown in Figure 3. The crystallographic data and the table with the distances and angles are collected in the Supplementary Materials (Tables S2 and S3, respectively). Rings A and C are coplanar as expected, but ring B deviates slightly from coplanarity relative to the condensed unit AC, showing a dihedral angle of 7.4°. The expected intramolecular hydrogen bond O2⋯H1 was observed with a distance of 1.84 Å. There were also a series of intermolecular hydrogen bonds, and it is worth noting the formation of dimers thanks to double head-to-tail hydrogen bonds involving H11 and O2 (see Figure S1 and Table S4).

### 3.2. Cytotoxic Activity

The antiproliferative activity of the four proligands **HL1**–**HL4** and their metal complexes was firstly evaluated in human colon (SW480) and lung (A549) cancer cells. The calculated half maximal inhibitory concentration, IC_50_ values, are gathered in Table 1. As can be observed, the ligand structure plays an essential role in the activity of the complexes. Among the ligands **HL1**–**HL3**, only **HL3** presents slight activity against A549 cells, whilst **HL4** is cytotoxic to both cell lines, with IC_50_ values lower than those found for the control cisplatin. The observed cytotoxicity of the ligands determines the cytotoxic potential of the corresponding metal complexes to some extent. While **L2**-complexes are inactive towards both cancer cell lines, **L1-Rh** and **L1-Ir** exhibit low or moderate cytotoxicity in the SW480 cell line, respectively. **L3-Rh** and **L3-Ir** are cytotoxic in A549 cells (being **L3-Ir** more active), while no activity against SW480 is observed. On the other hand, as was observed for **HL4**, its complexes present higher cytotoxic activity than cisplatin in both cell lines, and in particular **L4-Ir** is the most cytotoxic derivative among the **L4-M** family and among all the new complexes. Therefore, the results show that the activity of the chrysin derived metal complexes depends not only on the ligand (see Figure 2) but also on the nature of the metal center.

In view of the higher potential of **HL4** and its metal complexes as anticancer agents, an in-depth study was carried out with them. The cytotoxic activity of **HL4** and its metal complexes in a non-malignant lung fibroblast cell line (IMR-90) was evaluated in order to study the selectivity of the complexes under study against tumor cells (Table 2). According to the calculated selectivity index (SI = IC_50_(IMR-90)/IC_50_(A549)), the free ligand is not selective at all, being even more cytotoxic in the tested healthy cells than in tumor cells, while all the complexes display higher selectivity than cisplatin. The cytotoxicity against ovarian cancer cells sensitive (A2780) and resistant to cisplatin (A2780) was also evaluated (Table 2). Thus, **HL4** and its metal complexes are able to circumvent cisplatin resistance. What is more, **L4-Ru** and **L4-Rh** are more cytotoxic against cisplatin resistant cells than against sensitive cells.

### 3.3. NMR Stability Studies of the Complexes with **L4** in DMSO-d_6_ and DMSO-d_6_/D_2_O (3:2)

The study of the behavior of new anticancer agents in aqueous solution is crucial to reveal their stability. Moreover, when M–Cl bonds are present, it is important to determine whether the chloride ligand is exchanged by a water molecule to give the more reactive aqua-complex in aqueous media [76]. In fact, hydrolysis of M–Cl bonds is often an activation step for the reactivity of the transition metal anticancer complexes. Considering the higher cytotoxic activity of the complexes containing **L4**, stability studies were performed for the **L4-M** (M = Ru, Rh and Ir) derivatives. Due to the lack of solubility of these complexes in D_2_O, the biological studies were performed in a D_2_O/DMSO mixture (maximum amount of DMSO, 0.5%). Thus, the stability of freshly prepared solutions of the complexes in DMSO-*d*_6_ was studied first. Afterwards the aquation process was analyzed by addition of D_2_O over the previous solution (see Section 2). In spite of the fact that these NMR studies were necessarily performed in conditions of solvent and concentration different from those of the biological experiments, it is important to notice that they offer valuable information concerning the reactivity of the complexes.

#### 3.3.1. NMR Stability Studies of the Complexes Containing **L4** in DMSO-*d*_6_

The stability of the complexes **L4-M** (M = Ru, Rh and Ir) was studied in DMSO-*d*_6_ solution in order to know the speciation in the stock solution. After 24 h, there were significant differences in the stability of the complexes depending on the metallic center. All the studied compounds presented partial release of the chrysin ligand, as has been previously observed for similar Ru complexes with anionic O^O ligands [110,111]. The fact that, in the aromatic region, only another species is observed, apart from **HL4**, even after 24 h led us to tentatively propose that this species is the DMSO-*d*_6_ adduct, which will be noted as **L4-M**(**S**) ([ArM(L4)(DMSO)]^+^, Ar = *p*-cym or Cp*) [112].

The most stable complex in DMSO-*d*_6_ was **L4-Ru,** with an 8% of **HL4** in solution and 92% of **L4-Ru**(**S**) (see Figure S2 and Figure 2, spectrum a). For **L4-Rh**, although **L4-Rh(S)** is the major species, 39% of **HL4** (free ligand) was observed (see Figure S3). The presence of the **HL4** signals is concomitant with the apparition of a new Cp* resonance (1.62 ppm), which could correspond to species of the type [Cp*RhZ_3_]^n+^ (Z = Cl, H_2_O or DMSO) [113,114]. For complex **L4-Ir**, the ^1^H NMR spectra showed that the signals of the starting product decreased with time, while broad resonances appeared in the aromatic region (especially those for rings A and C, since ring B is less affected by the coordination of **HL4**, *vide supra*), along with a new Cp* signal at 1.63 ppm. The signal for the OH group was also observed (12.8 ppm). We propose that the signals for **HL4** are broad, because the free ligand is interacting with species of the type [Cp*IrZ_3_]^n+^ (Z = H_2_O or DMSO) [115] (see Figure S4).

#### 3.3.2. NMR Stability Studies of the Complexes with **L4** in DMSO-*d*_6_/D_2_O (3:2)

**Figure 4:**
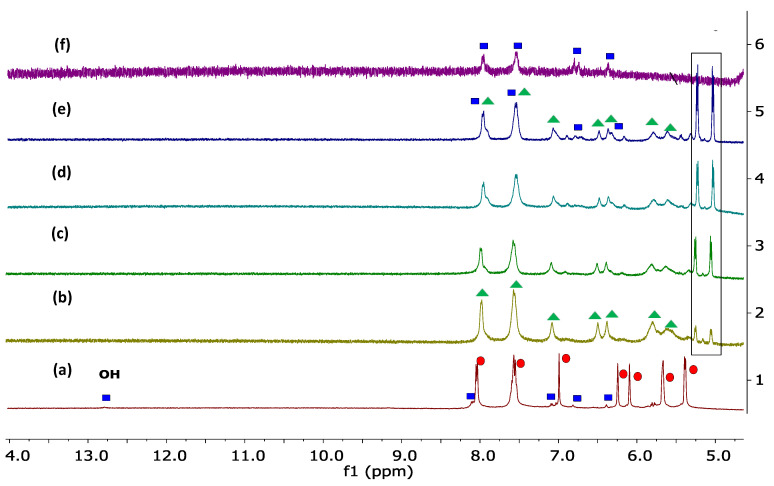
Aromatic area of ^1^H NMR spectra (400 MHz) for **L4-Ru**: in DMSO-*d*_6_ after 24 h; and in DMSO-*d*_6_:D_2_O (3:2) (400 MHz): (**a**) **L4-Ru** after 24 h in DMSO-*d*_6_; (**b**) t = 0; (**c**) t = 3 h; (**d**) t = 7 h; (**e**) t = 24 h; (**f**) free ligand **HL4**. Red circles, L4-Ru(S); green triangles, [(*p*-cym)Ru(L4)(H_2_O)]+; blue squares, **HL4**. The resonances inside the black rectangle correspond to [(*p*-cym)_2_Ru_2_(μ-OH)_3_]. Time values refer to the addition of D_2_O.

After addition of D_2_O over the solution of **L4-Ru(S)** in DMSO-*d*_6_ (see Experimental section and Figure 4), the signal for OH group disappeared from the spectrum by proton-deuterium exchange with D_2_O (compare a and b in Figure 2). The ^1^H NMR spectra showed the instantaneous formation of a new species that is proposed to be the aqua complex [(*p*-cym)Ru(**L4**)(H_2_O)]^+^. It has been reported that the solution of Ru(arene) compounds featuring an O^O-chelating motif and a chloride ligand in water often results in a rapid exchange of this chloride ligand by a water molecule [76,116,117]. The broadening of the aqua complex resonances may be due to interconversion processes, possibly involving protonation of the coordinated **L4** ligand [118]. The evolution with time of this aqua species to the formation of free **HL4** and the hydroxo dimer species [(*p*-cym)Ru(µ-OH)_3_Ru(*p*-cym)] [76,117,119] (two doublet signals at 5.03 and 5.23 ppm and a doublet at 1.10 ppm for the isopropylic methyl group, see Figure S5) is also observed. The formation of this dimer has been previously described for Ru-(*p*-cym)-flavonoids and similar complexes containing other O^O ligands [76,116,118,119]. The formation of the aqua complex should increase the acidity of the water molecule that could be able to protonate **L4,** giving rise to the partial formation of **HL4** and the hydroxo species. This has been previously proposed for relatively similar Ru and Os complexes [118]. The formation of the hydroxo dimer may favor the latter process (this type of behavior may also be possible for Rh or Ir complexes, *vide infra*). Thus, after 24 h, three species are present in solution: **HL4**, [(*p*-cym)_2_Ru_2_(µ-OH)_3_] and [(*p*-cym)Ru(**L4**)(H_2_O)]^+^.

After the addition of D_2_O over the solution of **L4-Rh(S)**, the aromatic region reflects the existence of a new set of single and broad signals (Figure S6). However, the aliphatic region (Figure S7) shows the existence of several Cp* containing species. We propose that the derivative containing ligand **L4** is [Cp*Rh(**L4**)(H_2_O)]^+^ that could be in fast equilibrium with other species resulting from dissociation of **L4** and/or H_2_O. The new Cp* resonances, that are shifted to higher field, should correspond to species with hydroxide groups, such as [(Cp*Rh)_2_(µ-OH)_3_]^+^ [114] or [(Cp*Rh)_2_(µ-OH)_2_Z_2_]^n+^ (Z = H_2_O, Cl) according to literature [113,114].

With respect to the solution of **L4-Ir(S)** in DMSO-*d*_6_, the addition of D_2_O leads to important changes in the ^1^H NMR spectra. In the aromatic region, the disappearance of the **L4-Ir(S)** signals, together with the formation of **HL4** and a new [Cp*Ir(**L4**)(H_2_O)]^+^ complex is observed (Figure S8). The amount of the aqua complex is reduced over time, while that of **HL4** is increased. In the aliphatic region (Figure S9) two main resonances are observed which are assigned to the aqua complex and the hydroxo dimer [(Cp*Ir)_2_(µ-OH)_3_]^+^ [120], whose amount increases with time. A small signal is also detected in the same position assigned to [Cp*Ir(Z)_3_]^n+^ (see above).

Thus, for the three complexes, the aquation process takes place with the concomitant formation of **HL4** and hydroxo derivatives, although in the case of **L4-Rh** a fast equilibrium is established amongst the different species in solution.

### 3.4. UV-Vis Stability Studies of the Complexes with **L4** in DMSO and Aqueous Solution

The stability of the complexes containing **L4** was performed by UV-Vis spectroscopy in DMSO observing that **L4-Ru** and **L4-Rh** present lower cleavage than its iridium analogue (See Figure S10). Additionally, the stability of the **L4-M** complexes was also studied by UV-Vis spectroscopy in the cacodylate (NaCaC) aqueous buffer pH 7. Spectrograms compiled in Figure S11 exhibit isosbestic points indicative of different species in solution. The functions obtained from abs-time data pairs are biexponential and point to an aquation process along with ligand release, confirming NMR observations. From the comparison of the rate constants obtained at pH = 7 and at slightly acidic pH value (pH = 5.5) it can be concluded that all **L4-M** complexes display less reactivity at pH = 5.5 than at pH = 7 (Figure S12).

### 3.5. Determination of the pK_a_ Values of **HL4**, **L4-Ru**, **L4-Rh** and **L4-Ir**

The acid-dissociation constants, pK_a_, were determined by recording the absorbance spectra of the free pro-ligand **HL4** and the **L4-M** complexes at different pH values, ranging from 3 to 11 in aqueous solution. Figure S13A shows the UV−vis absorbance spectra of **HL4**, and Figure S13B, C and D of **L4-Ru**, **L4-Rh** and **L4-Ir**, respectively. The absorbance-pH data pairs were examined and the pK_a_ values compiled in Table 3. Data were analyzed according to the Henderson-Hasselbalch equation [121] for complexes **L4-Ru**, **L4-Ir** and **HL4**, or according to the Ang equation [122] for **L4-Ir** that displayed two determinable equilibria.

**Scheme 2:**
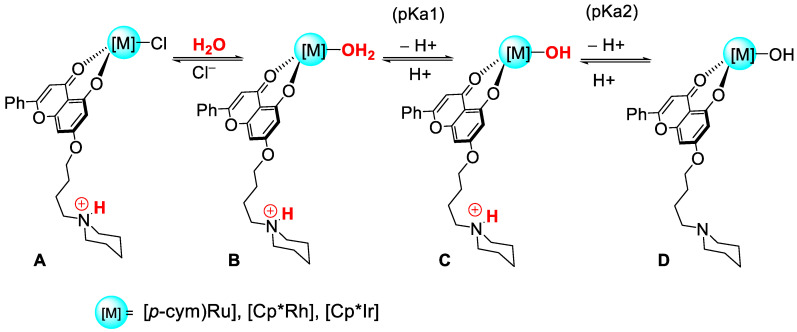
Aquation reaction of **L4-M** complexes and deprotonation processes corresponding to pK_a1_ and pK_a2_.

The ligand shows a single equilibrium (pK_a_ = 8.59) corresponding to the ionization of the chrysin hydroxyl group [123], but new bands observed at pH > 11 could be assigned to the NH^+^ dissociation of the piperidinium group, because the pK_a_ of the free piperidinium cation (protonated piperidine) is 11.22 [124]. Hence, under pH = 7, the proligand is mainly in the form of a monocation. By contrast, the spectrograms of **L4-M** complexes show two acid-base equilibria (Table 3), pK_a1_ and pK_a2_. In all cases, pK_a1_ corresponds to the proton dissociation of the coordinated water molecule to give the hydroxo complex [125,126]. According to these values, the acidity sequence of the coordinated H_2_O molecule is **L4-Ir** > **L4-Ru** > **L4-Rh**. Regarding pK_a2_, we propose that it corresponds to the dissociation of the piperidinium proton (NH^+^). In fact, only the value of pK_a2_ for **L4-Ir** could be accurately determined. Nevertheless, inspection of the spectral curves and Abs-pH data-pairs of **L4-Ru** and **L4-Rh** (Figure S13B,C) reveals that a second acid-base equilibrium is feasible, with pK_a2_ > 12 and pK_a2_ > 11 for **L4-Ru** and **L4-Rh**, respectively (see Scheme 2). From these results, we can conclude that under pH = 7, the three compounds are dicationic H_2_O-complexes, N is in the NH^+^ form and the aqua form predominates over the hydroxo complex (form B in Scheme 2). An interesting aspect that derives from the protonation of the piperidine fragment is that it enhances the amphiphilic character of the neutral chlorido complexes ([LMCl(O^O)]), since the positive charges are combined with hydrophobic motifs of the complexes such as the *p*-cymene or cyclopentadienyl rings and the alkyl groups of the cations.

### 3.6. Interaction with DNA

At this point, we can conclude that the identity of the chrysin derived ligands strongly influences the cytotoxicity of their metal complexes. Nonetheless, the role of the metal cannot be underestimated as far as stability and cytotoxic potential of the **L4**-complexes are concerned. DNA has been very often reported as the biological target for many metal complexes with anticancer activity [127,128]. Consequently, the interaction of the proligand **HL4** or **L4**-complexes with CT-DNA was investigated by different techniques such as melting temperature (T_m_) and viscosity measurement, as well as circular dichroism and agarose gel electrophoresis studies.

#### 3.6.1. Thermal Denaturation by UV-Vis

**Figure 5:**
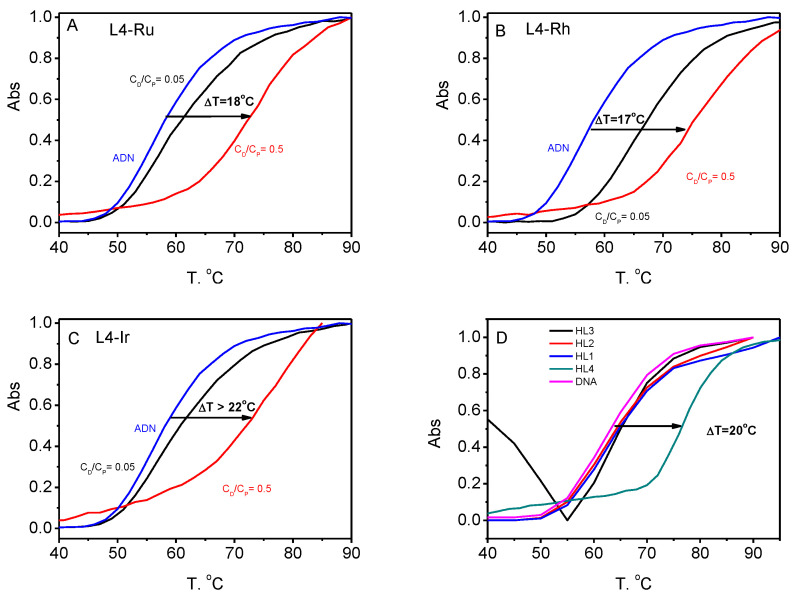
Melting profiles for (**A**) **L4-Ru**/DNA, (**B**) **L4-Rh**/DNA, (**C**) **L4-Ir**/DNA and (**D**) proligands **HL1**–**HL4** with DNA at C_D_/C_P_ = 0.5. C_P_ = 5.7 × 10^−5^ M. C_D_ = analytical concentration of the drug. C_P_ = analytical concentration of DNA.

**Figure 6:**
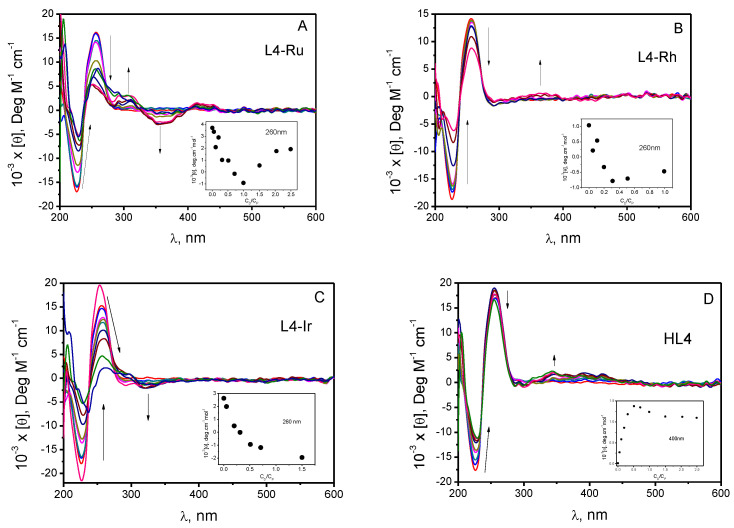
CD spectra for (**A**) **L4-Ru**/DNA, (**B**) **L4-Rh**/DNA, (**C**) **L4-Ir**/DNA and (**D**) **HL4**/DNA recorded with increasing drug concentration (arrow sense). C_P_ = 5.73 × 10^−5^ M, C_D_/C_P_ = 0–2.5. Inset: dependence of the molar ellipticity at 260 nm on the C_D_/C_P_ ratio.

T_m_ measurements were performed for fresh solutions in order to avoid decomposition processes. Figure 3A to Figure 4C revealed a great increase in the melting temperature compared to free DNA, (ΔT_m_ = 18 °C for **L4-Ru** (Figure 5A), ΔT_m_ = 17 °C for **L4-Rh** (Figure 5B) and ΔT_m_ > 22 °C in the case of **L4-Ir** (Figure 5C)). Such an increase in the melting temperature is typical of intercalation processes. Thus, intercalation of the **L4**-complexes between the DNA base pairs probably occurs through the ancillary ligand **L4**. Interestingly, only the cytotoxic proligand (see Table 1), **HL4** (green line–Figure 6D) is able to thermally stabilize the DNA double helix with a similar efficiency than its complexes (ΔT_m_ = 20 °C) [123].

#### 3.6.2. Circular Dichroism (CD)

Dichroism experiments expose the conformational variation in DNA structure upon drug interaction. The CD titrations of DNA with increasing concentrations of **HL4** and its three metal complexes were performed. Figure 6 reflects how each complex modifies the DNA spectrum in a different way.

The CD spectrum of the **L4-Ru**/DNA system showed that the molar ellipticity of both the negative and the positive bands of DNA decreased as the C_D_/C_P_ ratio rose (Figure 6A). Moreover, three induced circular dichroism bands (ICD) appeared with increasing C_D_/C_P_ values; namely, two positive bands centered at 300 nm and 360 nm, and a negative one at 425 nm, revealing the formation of new species. In a similar way, **L4-Rh** induced a decrease in the molar ellipticity of the DNA intrinsic bands along with the appearance of a small ICD band at 380 nm (Figure 6B). In addition, **L4-Ir** caused a diminution of the intensity of the DNA intrinsic bands along with a red shift of 6 nm of the DNA positive band and the appearance of a new negative ICD band at 321 nm (Figure 6C). The proligand **HL4** also induced a decrease in molar ellipticity of the DNA intrinsic CD bands and the appearance of a small ICD band (Figure 6D). These features in DNA titrations with **HL4** and **L4-M** complexes are related to the unwinding of the DNA double helix as a consequence of the drug intercalation between the DNA base pairs [129].

#### 3.6.3. Viscosity

**Figure 7:**
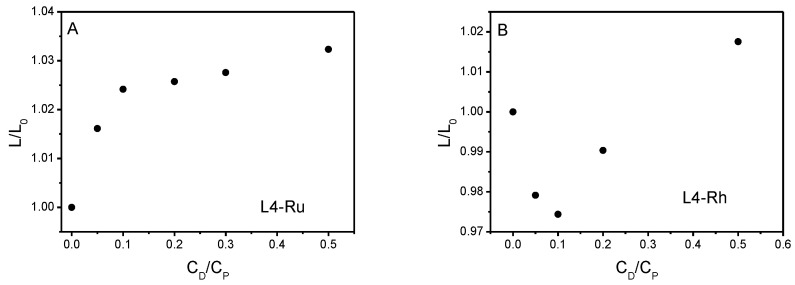

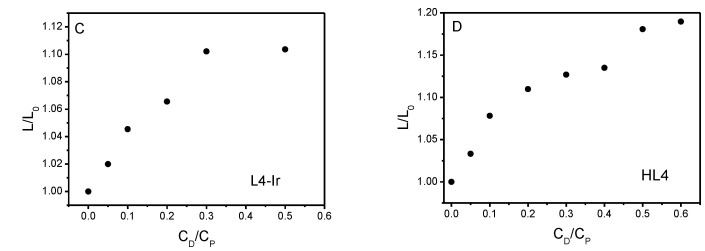
DNA relative contour length (L/L_0_) for (**A**) **L4-Ru**/DNA, (**B**) **L4-Rh**/DNA, (**C**) **L4-Ir**/DNA and (**D**) **HL4**/DNA systems as a function of the C_D_/C_P_ ratio. C_P_^0^ = 2.0 × 10^−4^ M.

Intercalation was corroborated by relative viscosity measurements (Figure 7). To enable the allocation of the drug in the intercalation site, DNA suffers a local unwinding which leads to DNA contour length enhancement. DNA saturation was observed at C_D_/C_P_ = 0.1, C_D_/C_P_ = 0.3 and C_D_/C_P_ = 0.2 for the **L4-Ru**/DNA, **L4-Ir**/DNA and **HL4**/DNA systems, respectively. For the **L4-Rh**/DNA system, a biphasic behavior was observed with a decrease in the DNA relative contour length up to C_D_/C_P_ = 0.1 followed by an increase from that point on (Figure 7B). Unfortunately, saturation was not observed for this system since precipitation occurs at high drug-DNA ratios (C_D_/C_P_ > 0.5). The highest DNA contour length enhancement was observed for the **HL4**/DNA system. These data confirm the influence of both the ligand and the metal center on the intercalation process [129].

#### 3.6.4. Agarose Gel Electrophoresis

**Figure 8:**
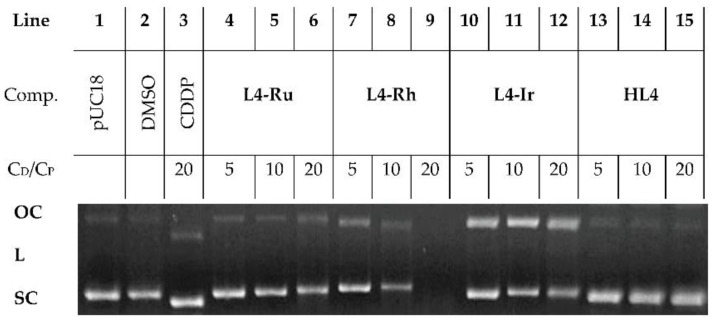
Electrophoresis mobility assay of pUC18 (20 µM) with DMSO (lane 2), cisplatin (lane 3), **L4-Ru** (lanes 4–6), **L4-Rh** (lanes 7–9), **L4-Ir** (lanes 10–12) and **HL4** (lanes 13–15) at different C_D_/C_P_ ratios. SC: supercoiled, OC: open circular and L: linear forms.

The electrophoretic mobility of the plasmid DNA pUC18 in the absence and in the presence of **HL4** and its metal complexes was studied (Figure 8) to enlighten the features of the interaction between DNA and the compound under study. The differences observed in the migration pattern between the plasmid alone (lane 1) or treated with the vehicle, DMSO (lane 2), and the plasmid incubated with different concentrations of **HL4** and **L4-M** confirmed DNA binding. It is clear that the amount of DMSO used in the experiments does not affect the migration pattern of the plasmid. On the other hand, the experiment with cisplatin (lane 3) which was included as a positive control for covalent binding, showed higher mobility of open circular (OC) and supercoiled (SC) forms than those observed for the free plasmid. In the experiments performed with plasmids treated with **L4-Ru** (lanes 4–6) and **L4-Rh** (lanes 7–9), the SC form migrates slower than in the assay with free plasmid and this retardation is dose-dependent. This behavior can be easily related to intercalation processes [130]. A vanishing of the plasmid bands at high drug-DNA concentrations ratio was observed for the **L4-Rh**/pUC18 system. This may occur due to plasmid saturation with the rhodium complex that prevents ethidium bromide staining, or due to precipitation of the large amount of the rhodium complex bound to DNA. The amount of the OC form is significantly increased in the presence of **L4-Ir** (lines 10–12), in contrast with its analogues. Thus, the iridium complex efficiently cleaved supercoiled DNA and this cleavage will be further studied. As expected, **HL4** shows a different trend in SC migration with respect to its complexes, which reinforces the hypothesis that the metal center plays a key role in the interaction with DNA [129].

**Figure 9:**
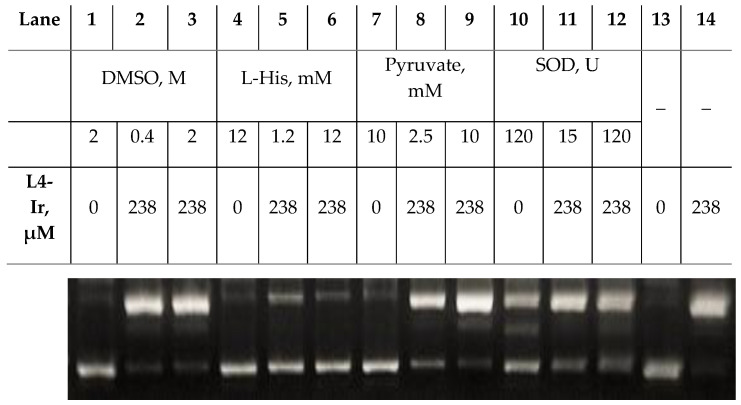
Cleavage electrophoresis of pUC18 (20 µM) incubated with **L4-Ir** or/and with DMSO (0.4 M and 2 M), L- histidine (1.2 mM and 12 mM), sodium pyruvate (2. 5 mM and 10 mM) or SOD (15 U and 120 U). Lane 13 is the control for pUC18 alone, and lane 14 is pUC18 with **L4-Ir**.

Since only the iridium complex display DNA chemical nuclease activity, the underlying mechanism was further investigated by means of a cleavage electrophoresis assay in which several well-known radical scavengers such as DMSO for OH^·^, L-histidine for ^1^O_2_, sodium pyruvate for H_2_O_2_ and superoxide dismutase (SOD) for O_2_^-^ were included (Figure 9) [129]. Treatment of pUC18 with a high concentration of **L4-Ir** (lane 14) causes the vanishing of the SC form and an increase in the amount of the OC and L topologies. In the presence of different amounts of DMSO (lanes 2 and 3) or pyruvate (lanes 8 and 9), DNA cleavage is not inhibited, whereas SOD (lanes 11 and 12) seems to partially circumvent the cleavage activity of **L4-Ir**. However, L-histidine efficiently inhibits the cleavage activity of **L4-Ir** (lanes 5 and 6), even at the lowest concentration. Therefore, ^1^O_2_ turns out to be the reactive oxygen species responsible for the DNA damage induced by **L4-Ir**. At this point, the metal center seems to be the key, since although **HL4** and **L4**-**M** complexes are able to interact with DNA only the iridium derivative cleaves DNA by an O_2_ dependent mechanism. The ability of **L4-Ir** to generate singlet oxygen constitutes an important advantage for its potential as antitumor agent over its analogues, since singlet oxygen is identified as one of the major ROS produced in mitochondria and altering its levels is reported as an efficient strategy for cancer therapy [131].

### 3.7. Intracellular ROS Generation

**Figure 10:**
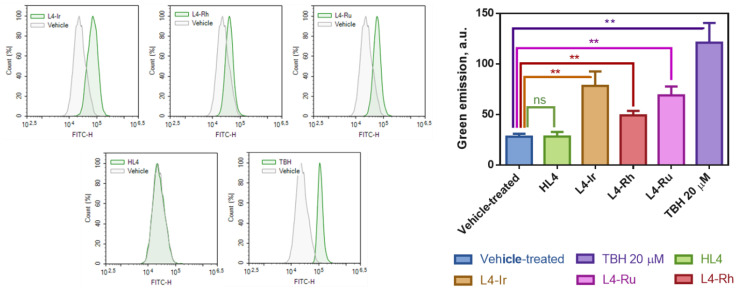
Flow cytometry plots of A549 cells treated with **HL4**, **L4-Ir**, **L4-Rh**, **L4-Ru** at their IC_50_ values during 24 h. Cells treated with 20 µM of TBH (tert-butyl-hydroperoxide) were included as positive control for H_2_O_2_ generation. Results are expressed as the mean ± SD and statistical significance is expressed as **, *p*-value < 0.01 (ANOVA–Dunnett).

Since cytotoxicity is often related to ROS generation in Ru(II) [40,132,133] and Ir(III) [133,134] half-sandwich complexes, the ability of **HL4** and its metal complexes to induce ROS generation in lung adenocarcinoma (A549) cells was studied by flow cytometry (FC) using H_2_DCFDA (dihydrodichlorofluorescein diacetate) as a probe for hydrogen peroxide, nitric oxide and peroxynitrite among other ROS [135]. More deeply, the oxidation of non-fluorescent H_2_DCFDA to highly fluorescent DCF (2′,7′-diclorofluorescein) by ROS produced inside the cells is monitored by FC. From the results compiled in Figure 10, we concluded that, unlike **HL4**, all its metal complexes increase ROS production, with the Ir complex inducing the greatest effect. ROS levels can be modulated by several reported mechanisms such as binding of the metal complex to GSH, thioredoxin reductase inhibition, catalytic oxidation of NADH and NADPH [36,136,137].

### 3.8. Apoptosis

**Figure 11:**
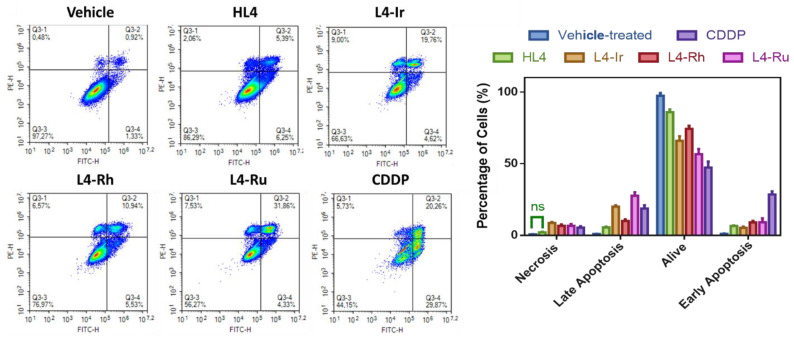
Examples of FC density plots of apoptosis studies in A549 cells treated with **HL4**, **L4-Ir**, **L4-Rh**, **L4-Ru** and **CDDP** at their IC_50_ values during 24 h and staining with annexin V–FITC/PI. The results are expressed as the mean ± SD of at least two different experiments with two replicates. The values achieved are statistically significant results (*p*-value < 0.01–ANOVA–Dunnett) except if no significance (ns) is stated.

ROS generation leads to oxidative stress and can trigger apoptosis. In order to confirm if apoptosis is the mechanism of cell death caused by the compounds under study, apoptosis induction was studied by flow cytometry. As revealed by the respective FC plots (Figure 11), the population of cells in necrosis, late and early apoptosis are significantly increased upon treatment with metal complexes. By contrast, treatment with the ligand only induces apoptosis. These results suggest that the oxidative stress caused upon exposure to our metal complexes is moderate since apoptosis is the preferred pathway for cell death. On the other hand, the differences observed in the ability to induce ROS generation among the metal complexes may be correlated to the percentage of necrotic cells. That is, **Ir-L4** exhibits both the greatest ROS production and the biggest population of necrotic cells pointing out to a higher level of oxidative stress than that observed after the treatment with the Ru or Rh analogues.

### 3.9. Cell Cycle Studies

**Figure 12:**
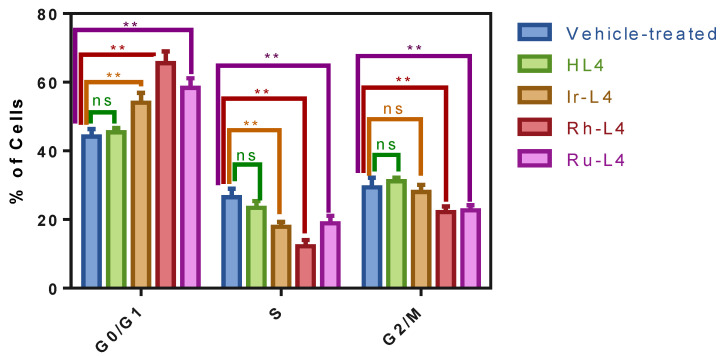
Flow cytometry analysis of cell cycle distribution of A549 cells treated during 24 h with the compounds under study. Data were obtained from duplicates of two different experiments and plot as mean values ± SD. ns No significance and ** Statistical significance, *p*-value < 0.01 (ANOVA-Dunnett).

Cell cycle arrest brings more opportunities for cells to enter apoptosis which is a desirable effect in chemotherapeutic drugs. Cell cycle progression in A549 cells exposed to **HL4** and their metal complexes for 24 h was studied by flow cytometry. In short, treatment with **HL4** does not induce cell cycle arrest, whereas treatment with the metal complexes induces cell cycle arrest in G0/G1 along with a drop in S and G2/M populations (Figure 12). Cell cycle arrest in G0/G1 is a common feature in cells treated with ROS generating chemotherapeutic agents.

### 3.10. Cellular Uptake

**Figure 13:**
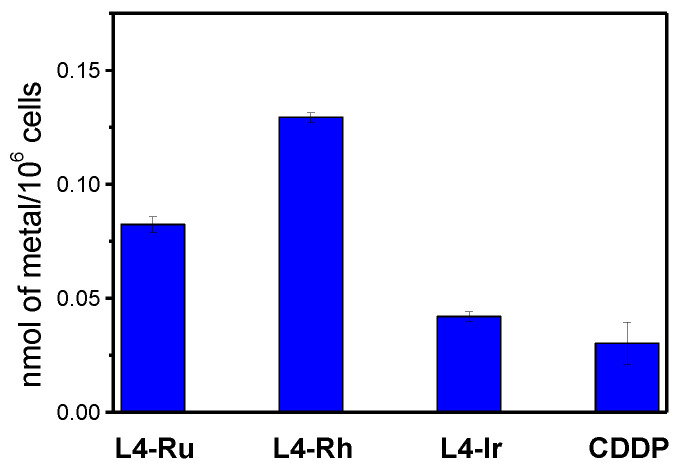
Metal accumulation in A549 cells after 24 h of treatment with 5 µM of the compounds under study. Cisplatin is included for comparison.

In addition to differences in biological behavior, such as the generation of ROS, the differences in the cytotoxicity amongst different compounds may also be related to their success in getting into cells. Hence, inductively coupled plasma mass spectrometry (ICP-MS) was employed to quantify the amount of metal inside A549 cells treated with the **L4**-complexes during 24 h (Figure 13) [129]. The accumulation of all the complexes was higher than that determined for cisplatin. There is no correlation between metal internalization and cytotoxic activity in A549 cells. In fact, the most cytotoxic complex (**L4-Ir**) turned out to be the one with the lowest cellular uptake, which supports the hypothesis of a dual mechanism of action. DNA binding and ROS formation provide this complex with a great effectivity in terms of cytotoxicity in spite of its low accumulation inside the cells. Moreover, although the cellular uptake of **L4-Rh** is quite higher than that of **L4-Ru**, their cytotoxic activities are very similar, with no notable differences in terms of DNA interaction. In order to explain this lack of correlation, the potential sequestration of **L4**-**M** complexes by serum proteins (BSA) was analyzed in the next section.

### 3.11. Interaction with BSA

**Figure 14:**
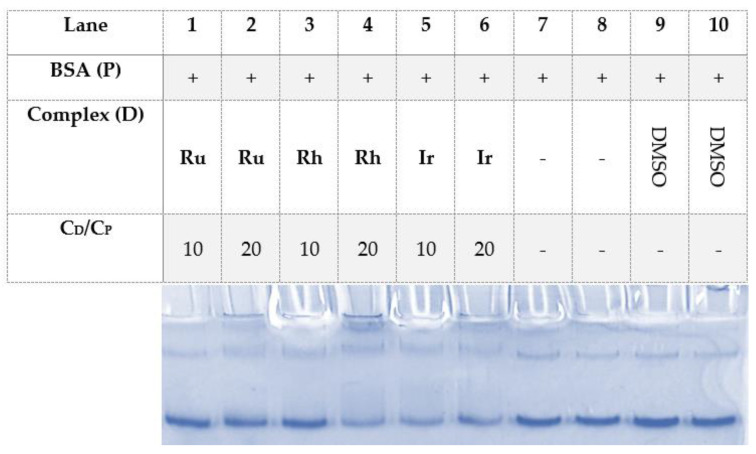
Native PAGE electrophoresis of BSA incubated during 24 h with different concentrations of the **L4-M** complexes. C_P_ = 1.5 µM. C_D_/C_P_ = 10 and 20. C_P_ = BSA concentration; C_D_ = complexes concentration.

Protein binding was found to be a determining factor in the tumor accumulation of some new metal complexes and consequently, in their anticancer activity [138]. By contrast, is has also been described that binding to serum proteins may be a limiting factor for cytotoxicity since serum proteins can sequestrate metal complexes preventing their access to their target and limiting their cytotoxicity [139]. With this in mind, we have studied the interaction of the **L4**-**M** complexes with bovine serum album (BSA) due to its high similarity to human serum albumin (HSA, one of the most abundant proteins present in blood plasma) by native acrylamide gel electrophoresis [129]. Figure 14 shows BSA migration after being incubated with the complexes in the dark for 24 h at two different drug-BSA ratios: 10 and 20. Negative controls of BSA alone (lanes 7 and 8) or vehicle-DMSO (BSA in the presence of the maximum DMSO concentration used in the experiment, lanes 9 and 10) are included. Complex **L4-Ru** is not able to induce conformational changes in BSA (lanes 1 and 2). Complex **L4-Rh** alters BSA structurally only at higher concentrations C_D_/C_P_ = 20 (lane 4). These results do not explain the differences in the cellular uptake of Ru and Rh derivatives. Noteworthy, **L4-Ir,** which is the most cytotoxic complex, affects the protein conformation in a stronger way than its congeners (lanes 5 and 6). Circular Dichroism experiments of BSA in presence of **HL4** or its metal complexes confirm the electrophoresis conclusions. Figure S14 shows higher interaction of BSA with **L4-Ir** compared to **L4-Rh** and **L4-Ru**. This fact can be easily correlated with the low accumulation of **L4-Ir** inside the cells due to its partial sequestration by serum proteins.

## 4. Conclusions

With the aim of obtaining multitargeted metallodrugs including a bioactive ligand, a family of 12 half sandwich iridium, rhodium and ruthenium complexes with a series of chrysin-derived ligands **L1-L4** containing different substituents have been synthesized and characterized. The antiproliferative activity of proligands **HL1-HL4** and their complexes was tested on SW480 and A549 cell lines and clear effects attributed to the metal center and the ligand were found. The most active species in both cell lines were the piperidine pro-ligand **HL4** and its metal complexes **L4-M**, with the following cytotoxic potency order **L4-Ir > L4-Ru ≈ L4-Rh**. All of them had a cytotoxic effect higher than cisplatin. Furthermore, **L4-M** complexes exhibited more selectivity towards cancer cells than **HL4** and cisplatin. **HL4** and **L4-M** were able to circumvent resistance in cisplatin resistant ovarian cancer cells (A2780cis). From the measurements of the pK_a_ values, it is concluded that the piperidine fragment is protonated under biological conditions, a fact that may increase the amphiphilic character of the neutral chlorido-complexes of formula [LMCl(O^O)].

The cellular uptake of the piperidine containing-complexes was evaluated and the following order was found: **L4-Rh** > **L4-Ru** > **L4-Ir**. The low accumulation of **L4-Ir** was explained by the interaction with BSA that reflects a partial sequestration by the blood serum proteins.

The ligand **HL4** and its **L4-M** complexes are able to interact with double stranded DNA by intercalation, which was confirmed by CD, melting and viscosity experiments. Besides, complex **L4-Ir** exhibited DNA chemical nuclease activity through the generation of ROS (Reactive Oxygen Species), more specifically, ^1^O_2_. This supports the hypothesis of a dual mechanism of action and provides this complex with a great effectiveness in terms of cytotoxicity, in spite of its low accumulation inside the cells.

Evidence of the different biological behavior of the proligand **HL4** respecting its **L4-M** complexes were clearly observed “in cellula” experiments. The metal complexes were able to induce ROS generation and block cell cycle progression in G0/G1 phase, whereas no effect was observed for the proligand. In any case, all the piperidine derivatives triggered apoptosis.

An important conclusion of this work is the significant effect that the introduction of the piperidine fragment in the ligand had on the antiproliferative behavior of the metal complexes, a fact that could be applied for future research, even using known metallodrugs.

## Figures and Tables

**Table 1:** IC_50_ values expressed in µM after an exposition period of 24 h for all the synthesized complexes and their ligands in colon (SW480) and lung (A549) cancer cell lines.

Complex	SW480	A549	Complex	SW480	A549
**L1-Ru**	95.7 ± 5.2	159.9 ± 1.2	**L3-Ru**	117.3 ± 3.2	114.9 ± 1.2
**L1-Rh**	33.5 ± 1.1	134.4 ± 1.1	**L3-Rh**	>200	57.3 ± 1.1
**L1-Ir**	51.9 ± 1.6	>200	**L3-Ir**	>200	36.8 ± 1.2
**HL1**	>200	>200	**HL3**	>200	63.7 ± 1.2
**L2-Ru**	134.1 ± 9.2	122.6 ± 1.2	**L4-Ru**	28.5 ± 1.3	31.1 ± 1.6
**L2-Rh**	124.2 ± 1.1	97.4 ± 1.2	**L4-Rh**	31.3 ± 1.3	35.3 ± 1.0
**L2-Ir**	119.8 ± 1.1	>200	**L4-Ir**	15.9 ± 1.3	18.9 ± 1.1
**HL2**	>200	>200	**HL4**	20.8 ± 2.0	23.8 ± 2.1
**CDDP** ^1^	46.7 ± 1.4	37.6 ± 1.3			

^1^ CDDP = Cisplatin.

**Table 2:** IC_50_ values expressed in µM after an exposition period of 24 h for **HL4** and **L4** complexes, in healthy human lung fibroblasts (IMR-90) and ovarian cancer cells (A2780) sensitive and resistant to cisplatin (A2780cis). Calculated selectivity index (SI = IC_50_(IMR-90)/IC_50_(A549)) and resistance factor (RF = IC_50_(A2780cis)/ IC_50_(A2780)).

Complex	IMR-90	SI	A2780	A2780cis	RF
**L4-Ru**	54.5 ± 1.1	1.8	14.9 ± 1.8	8.4 ± 0.4	0.6
**L4-Rh**	55.6 ± 1.0	1.6	23.3 ± 2.1	4.9 ± 0.2	0.2
**L4-Ir**	33.8 ± 1.2	1.8	22.7 ± 3.0	23.5 ± 1.8	1.0
**HL4**	5.1 ± 1.3	0.2	8.8 ± 0.6	13.0 ± 1.6	1.5
**CDDP** ^1^	57.7 ± 1.2	1.5	7.0 ± 0.7	60.1 ± 4.3	8.6

^1^ CDDP = Cisplatin.

**Table 3:** pK_a_ values obtained for **HL4** and **L4-M**.

Complex	pK_a_	pK_a1_	pK_a2_
**L4-Ru**		9.56 ± 0.08	>12
**L4-Rh**		9.67 ± 0.03	>11
**L4-Ir**		9.02 ± 0.01	11.41 ± 0.04
**HL4**	8.59 ± 0.04		>11

## Data Availability

The data presented in this study are available on request from the corresponding author.

## Supplementary Materials

The following are available online at https://www.mdpi.com/article/10.3390/pharmaceutics13101540/s1. Synthesis and characterization of the new complexes: ^1^H and ^13^C{^1^H} NMR spectra of **HL1**-**HL4** and **M-L** (M = Ru, Rh, Ir; L = L1, L2, L3, L4), Table S1: ^1^H NMR data for the chrysin-derived ligands in the **L-M** (L = L1–L4 and M = Ru, Rh, Ir) complexes in CDCl_3_, Table S2: Crystal data and structure refinement for **HL2**, Table S3: Bond lengths [Å] and angles [°] for **HL2**, Figure S1: dimers formed in proligand **HL2** by formation of double head-to-tail hydrogen bonds, Table S4: Data for the hydrogen bonds formed in **HL2**, Figure S2: Aromatic area of ^1^H NMR spectra of **L4-Ru** in DMSO-*d*_6_ (400 MHz), Figure S3: Aromatic area of ^1^H NMR spectra of **L4-Rh** in DMSO-*d*_6_ (400 MHz), Figure S4: Aromatic area of ^1^H NMR spectra of **L4-Ir** in DMSO-*d*_6_ (400 MHz), Figure S5: Aliphatic area of ^1^H NMR spectra of **L4-Ru** in DMSO-*d*_6_:D_2_O (3:2) (400 MHz), Figure S6: Aromatic area of ^1^H NMR spectra of **L4-Rh** in DMSO-*d*_6_:D_2_O (3:2) (400 MHz), Figure S7: Aliphatic area of ^1^H NMR spectra of **L4-Rh** in DMSO-*d*_6_:D_2_O (3:2) (400 MHz), Figure S8: Aromatic area of ^1^H NMR spectra of **L4-Ir** in DMSO-*d*_6_:D_2_O (3:2) (400 MHz), Figure S9: Aliphatic area of ^1^H NMR spectra of **L4-Ir** in DMSO-*d*_6_:D_2_O (3:2) (400 MHz), Figure S10: Absorbance spectra over time of (A) **L4-Ru**, (B) **L4-Rh**, (C) **L4-Ir**, in DMSO. CD = 1.66 × 10^−5^ M, Figure S11: Absorbance spectra over time in buffer solution of (A) **L4-Ru**, (B) **L4-Rh** and (C) **L4-Ir**. CD = 3.30 × 10^−5^ M, Figure S12: Kinetic traces and biexponential fitting results of **L4-Ru**, **L4-Rh** and **L4-Ir** in buffer solution at pH = 5.5 and pH = 7, Figure S13: Absorbance spectra of (A) **HL4**, (B) **L4-Ru**, (C) **L4-Rh**, (D) **L4-Ir**, recorded in the pH 3–11 range. Inset: pH effect on the complexes absorbance at (A) 268 nm, (B) 267 nm, (C) 262 nm, (D) 385 nm. CD = 3.3 × 10^−5^ M, Figure S14: CD spectra of BSA in the absence and in the presence of the **L4-M** complexes (M = Ru, Rh and Ir) at a [**L4-M**]/[BSA] concentration ratio of 5. CBSA = 0.5 µM, CDMSO = 0.002%, I = 2.5 mM sodium cacodylate (NaCac), pH = 7.4 and T = 25 °C.
