# Supplementary material for: Anticancer Activity of Half-Sandwich Ru, Rh and Ir Complexes with Chrysin Derived Ligands: Strong Effect of the Side Chain in the Ligand and Influence of the Metal
Source: Pharmaceutics. 2021 Sep 23;13(10):1540. doi: 10.3390/pharmaceutics13101540 (PMC8537477; doi:10.3390/pharmaceutics13101540)
Supplement: Supplementary file 1 [file pharmaceutics-13-01540-s001.zip › pharmaceutics-1344276-supplementary.pdf]

pharmaceutics

MDPI

Supplementary Materials: Anticancer Activity of Half-sandwich Ru, Rh and Ir Complexes with Chrysin Derived Ligands: Strong Effect of the Side Chain in the Ligand and Influence of the Metal.

Ana Rosa Rubio, Rocío González, Natalia Busto, Mónica Vaquero, Ana L. Iglesias, Félix A. Jalón, Gustavo Espino,

Ana M. Rodríguez, Begoña García, Blanca R. Manzano

Synthesis and characterization of the new complexes

See Chart 1 for the atom numbering scheme

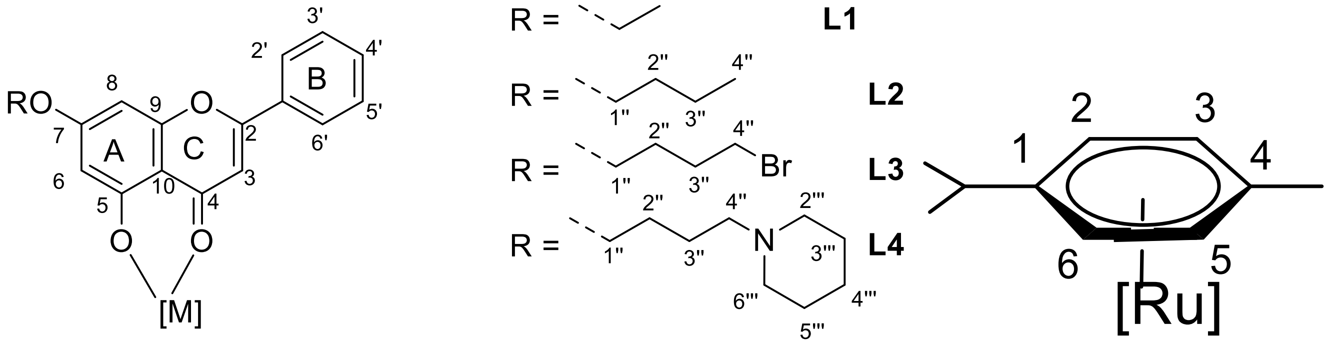

Chart 1

Synthesis of [RuCl(η⁶-*p*-cym)L1], L1-Ru. HL1 (0.097 g, 0.344 mmol) and $NaHCO_3$ (0.029 g, 0.345 mmol) were added to 30 mL of MeOH. The yellow-white suspension was heated under reflux for 2 hours. A yellow solution is obtained that was allowed to get to r.t. and the solvent was carefully removed. The yellow solid obtained was not completely dried. A previously prepared orange solution of $[RuCl_2(\eta^6\text{-}p\text{-cym})]_2$ (0.100 g, 0.163 mmol) in 30 mL of CH_2Cl_2 was added to this solid. The suspension was heated at reflux for 20 hours and a solution, with a small amount of solid, was obtained that was filtered and the solvent was evaporated to dryness. The orange solid obtained was washed with an acetone/hexane (1:1) mixture (10 x 8 mL, until the solution has a very pale orange color). The solid was dried after each washing. The solvents were gathered and evaporated to dryness. **L1-Ru** was obtained as a dark orange microcrystalline solid (0.143 g). Yield = 79 %. Anal. Calcd for $C_{27}H_{27}ClO_4Ru\cdot0.2CH_2Cl_2$ (%): C 57.41, H 4.85 Found: C 57.51, H 4.65. CH_2Cl_2 was observed

in the ^{1}H NMR spectrum. ^{1}H RMN (500 MHz, $CDCl_3$, 298 K): δ 7.82 (m, 2H, $H^{2',6'}$), 7.52 – 7.45 (m, 3H, $H^{3',4',5'}$), 6.72 (s, 1H, H^3), 6.37 (d, $^4J_{HH}$ = 2.4 Hz, 1H, H^6), 6.14 (d, $^4J_{HH}$ = 2.4 Hz, 1H, H^8), 5.54 (td, $^3J_{HH}$ = 6.0 Hz, $^4J_{HH}$ = 1.0 Hz, 2H, $H^{2,6}$ *p*-cym), 5.26 (d, $^3J_{HH}$ = 6.0 Hz, 2H, $H^{3,5}$ *p*-cym), 4.05 (q, $^3J_{HH}$ = 7.0 Hz, 2H, OCH_2), 3.01 (sept, $^3J_{HH}$ = 6.9 Hz, 1H, CH ^{i}Pr *p*-cym), 2.33 (s, 3H, Me *p*-cym), 1.39 (t, $^3J_{HH}$ = 7.0 Hz, 3H, OCH_2CH_3), 1.37 (d, $^3J_{HH}$ = 6.9 Hz, 3H, Me ^{i}Pr *p*-cym), 1.36 (d, $^3J_{HH}$ = 6.9 Hz, 3H, Me ^{i}Pr *p*-cym). ^{13}C{^{1}H} RMN (126 MHz, $CDCl_3$, 298 K): δ 178.10 (C^4), 167.80 (C^5 or C^7 or C^2), 165.34 (C^5 or C^7 or C^2), 161.04 (C^5 or C^7 or C^2), 158.60 (C^9), 131.53 ($C^{1'}$ or $C^{4'}$), 131.37 ($C^{1'}$ or $C^{4'}$), 129.04 ($C^{3',5'}$), 126.23 ($C^{2',6'}$), 106.93 (C^3 or C^{10}), 104.69 (C^3 or C^{10}), 101.14 (C^6), 99.65 (C^1 *p*-cym), 97.11 (C^4 *p*-cym), 90.73 (C^8), 82.51, 82.45 (2 x s, $C^{2,6}$ *p*-cym), 78.96, 78.91 (2 x s, $C^{3,5}$ *p*-cym), 63.69 (OCH_2), 30.95 (s, CH ^{i}Pr *p*-cym), 22.57, 22.52 (2 x s, 2 x Me ^{i}Pr *p*-cym), 18.19 (s, Me *p*-cym), 14.83 (s, OCH_2CH_3). IR: selected band, 1630 cm^{-1}, ν(C=O). MS (FAB+): m/z (%) 517 (89) $[M-Cl]^+$.

Synthesis of [RuCl(η^6-*p*-cym)L2], L2-Ru. The method was similar to that described for **L1-Ru**. Amounts were as follows: 0.213 g (0.686 mmol) of **HL2**, 0.058 g, (0.690 mmol) of $NaHCO_3$ and 60 mL of MeOH, in the first step of the reaction and 0.200 mg (0.327 mmol) of $[RuCl_2(\eta^6\text{-}p\text{-cym})]_2$ and 60 mL of CH_2Cl_2 in the second step. The purification was similar to that of **L1-Ru** but the mixture of solvents for the washings was acetone/hexane 1:2. **L2-Ru** was obtained as a dark orange microcrystalline solid (0.280 g). Yield = 74 %. Anal. Calcd for $C_{29}H_{31}ClO_4Ru\cdot CH_2Cl_2$ (%): C 54.18, H 5.00. Found: C 54.46, H 4.67. CH_2Cl_2 was observed in the ^{1}H NMR spectrum. ^{1}H RMN (500 MHz, $CDCl_3$, 298 K): δ 7.82 (m, 2H, $H^{2',6'}$), 7.52 – 7.45 (m, 3H, $H^{3',4',5'}$), 6.72 (s, 1H, H^3), 6.37 (d, $^4J_{HH}$ = 2.3 Hz, 1H, H^6), 6.14 (d, $^4J_{HH}$ = 2.3 Hz, 1H, H^8), 5.54 (m, 2H, $H^{2,6}$ *p*-cym), 5.26 (m, 2H, $H^{3,5}$ *p*-cym), 3.98 (t, $^3J_{HH}$ = 6.5 Hz, 2H, $H^{1''}$), 3.01 (sept, $^3J_{HH}$ = 6.9 Hz, 1H, CH ^{i}Pr *p*-cym), 2.33 (s, 3H, Me *p*-cym), 1.74 (q, J_{HH} = 6.5, 7.4 Hz, 2H, $H^{2''}$), 1.45 (sex, $^3J_{HH}$ = 7.4, 2H, $H^{3''}$), 1.38 (d, $^3J_{HH}$ = 6.9 Hz, 3H, Me ^{i}Pr *p*-cym), 1.37 (d, $^3J_{HH}$ = 6.9 Hz, 3H, Me ^{i}Pr *p*-cym), 0.96 (t, $^3J_{HH}$ = 7.4 Hz, 3H, $H^{4''}$). ^{1}H RMN (500 MHz, acetone-d^6, 298 K): δ 8.02 (m, 2H, $H^{2',6'}$), 7.61 – 7.54 (m, 3H, $H^{3',4',5'}$), 6.82 (s, 1H, H^3), 6.18 (d, $^3J_{HH}$ = 2.4 Hz, 1H, H^6), 6.15 (d, $^3J_{HH}$ = 2.4 Hz, 1H, H^8), 5.60 (m, 2 x H, $H^{2,6}$ *p*-cym), 5.28 (m, 2 x H, $H^{3,5}$ *p*-cym), 4.03 (m, 2H, $H^{1''}$), 2.96 (sept, $^3J_{HH}$ = 7.0 Hz, 1H, CH ^{i}Pr *p*-cym), 2.24 (s, 3H, Me *p*-cym), 1.75 (m, 2H, $H^{2''}$), 1.49 (m 2H, $H^{3''}$), 1.39 (d, $^3J_{HH}$ = 7.0 Hz, 3H, Me ^{i}Pr *p*-cym), 1.37 (d, $^3J_{HH}$ = 7.0 Hz, 3H, Me ^{i}Pr *p*-cym), 0.97 (t, $^3J_{HH}$ = 7.4 Hz, 3H, $H^{4''}$). ^{13}C{^{1}H} RMN (126 MHz, $CDCl_3$, 298 K): δ 178.14 (C^4), 167.84 (C^5 or C^7 or C^2), 165.62 (C^5 or C^7 or C^2), 161.07 (C^5 or C^7 or C^2), 158.64 (C^9), 131.55 ($C^{1'}$ or $C^{4'}$), 131.45 ($C^{1'}$ or $C^{4'}$), 129.08 ($C^{3',5'}$), 126.28 ($C^{2',6'}$), 106.96 (C^3 or C^{10}), 104.72 (C^3 or C^{10}), 101.23 (C^6), 99.71 (C^1 *p*-cym), 97.15 (C^4 *p*-cym), 90.80 (C^8), 82.56, 82.49 (2 x s, $C^{2,6}$ *p*-cym), 79.01, 78.97 (2 x s, $C^{3,5}$ *p*-cym), 67.91 ($C^{1''}$), 31.24 ($C^{2''}$), 31.00 (CH ^{i}Pr *p*-cym), 22.62, 22.58 (2 x s, 2 x Me ^{i}Pr *p*-cym), 19.32 ($C^{3''}$), 18.23 (Me *p*-cym), 13.91 ($C^{4''}$). IR: selected band, 1634 cm^{-1} (C=O). MS (FAB+): m/z (%) 545 (100) $[M-Cl]^+$.

Synthesis of [RuCl(η⁶-*p*-cym)L3], L3-Ru. The method was similar to that described for **L1-Ru**. Amounts were as follows: 0.133 g (0.342 mmol) of **HL3**, 0.029 g (0.345 mmol) of $NaHCO_3$ and 30 mL of MeOH, in the first step of the reaction and 0.100 mg (0.163 mmol) of $[RuCl_2(\eta^6\text{-}p\text{-cym})]_2$ and 30 mL of CH_2Cl_2 in the second step. The purification was similar to that of **L1-Ru** but the mixture of solvents for the washings was acetone/hexane 1:4. **L3-Ru** was obtained as a dark orange microcrystalline solid (0.164 g). Yield = 76 %. Anal. Calcd for $C_{29}H_{30}BrClO_4Ru$ (%): C 52.86, H 4.59 Found: C 52.55, H 4.37. ^{1}H RMN (500 MHz, $CDCl_3$, 298 K): δ 7.82 (m, 2H, $H^{2',6'}$), 7.53 – 7.45 (m, 3H $H^{3',4',5'}$), 6.73 (s, 1H, H^3), 6.36 (d, $^4J_{HH}$ = 2.4 Hz, 1H, H^6), 6.14 (d, $^4J_{HH}$ = 2.4 Hz, 1H, H^8), 5.54 (m, 2H, $H^{2,6}$ *p*-cym), 5.26 (m, 2H, $H^{3,5}$ *p*-cym), 4.03 (t, $^3J_{HH}$ = 6.0 Hz, 2H, $H^{1''}$), 3.47 (t, $^3J_{HH}$ = 6.5 Hz, 2H, $H^{4''}$), 3.01 (sept, $^3J_{HH}$ = 6.9 Hz, 1H, CH ^{i}Pr *p*-cym), 2.33 (s, 3H, Me *p*-cym), 2.03 (m, 2H, $H^{3''}$), 1.45 (m, 2H, $H^{2''}$), 1.38 (d, $^3J_{HH}$ = 6.9 Hz, 3H, Me ^{i}Pr *p*-cym), 1.37 (d, $^3J_{HH}$ = 6.9 Hz, 3H, Me ^{i}Pr *p*-cym). ^{13}C{^{1}H} RMN (126 MHz, $CDCl_3$, 298 K): δ 178.14 (C^4), 167.85 (C^5 or C^7 or C^2), 165.13 (C^5 or C^7 or C^2), 161.08 (C^5 or C^7 or C^2), 158.58 (C^9), 131.56 ($C^{1'}$ or $C^{4'}$), 131.29 ($C^{1'}$ or $C^{4'}$), 129.04 ($C^{3',5'}$), 126.21 ($C^{2',6'}$), 107.05 (C^3 or C^{10}), 104.70 (C^3 or C^{10}), 101.11 (C^6), 99.63 (C^1 *p*-cym), 97.08 (C^4 *p*-cym), 90.61 (C^8), 82.51, 82.44 (2 x s, $C^{2,6}$ *p*-cym), 78.95, 78.89 (2 x s, $C^{3,5}$ *p*-cym), 66.95 ($C^{1''}$), 33.46 ($C^{4''}$), 30.93 (CH ^{i}Pr *p*-cym), 29.47 ($H^{3''}$), 27.75 ($C^{2''}$), 22.56, 22.51 (2 x s, 2 x Me ^{i}Pr *p*-cym), 18.17 (s, Me *p*-cym). IR: selected band, 1636 cm^{-1} ν(C=O). MS (FAB+): m/z (%) 623 (96) $[M\text{-}Cl]^+$.

Synthesis of [RuCl(η⁶-*p*-cym)L4], L4-Ru. A mixture of ligand **HL4** (0.254 g, 0.267 mmol) and $NaHCO_3$ (0.0224 g, 0.254 mmol), was dissolved in 40 mL of methanol and refluxed for 2h; after cooling to room temperature, the solvent was removed *in vacuo* forming a yellowish residue (flavonate salt). A solution of $[Ru(p\text{-cymene})Cl_2]_2$ (0.078 g, 0.127 mmol) in dichloromethane (30 mL), was added dropwise to the yellow residue and refluxed overnight. The mixture was allowed to cool to room temperature and filtered *via* cannula to eliminate the NaCl formed. The red solution was evaporated *in-vacuo* to yield a red solid that was washed (3 x 5 mL) with hexane. Recrystallization from THF/hexane vapor diffusion system, gave red-orange crystals of **L4-Ru**. Yield (0.1391g 82.6%). Anal. Calcd for $C_{34}H_{40}ClNO_4Ru\cdot1.25CH_2Cl_2$ (%): C 55.03, H 5.57, N 1.82. Found: C 54.88, H 5.24, N, 1.70. CH_2Cl_2 was observed in the ^{1}H NMR spectrum.^{1}H RMN (500 MHz, $CDCl_3$, 298 K): δ 7.82 (m, 2H, $H^{2',6'}$), 7.52 – 7.46 (m, 3H, $H^{3',4',5'}$), 6.72 (s, 1H, H^3), 6.36 (d, J_{HH} = 2.4 Hz, 1H, H^6), 6.14 (d, J_{HH} = 2.4 Hz, 1H, H^8), 5.54 (m, 2H, $H^{2,6}$ *p*-cym), 5.26 (m, 2H, $H^{3,5}$ *p*-cym), 3.99 (t, $^3J_{HH}$ = 6.4 Hz, 2H, $H^{1''}$), 3.01 (sept, $^3J_{HH}$ = 6.9 Hz, 1H, CH ^{i}Pr *p*-cym), 2.42-2.32 (m, 6H, $H^{4''}$ + $H^{2''',6'''}$), 2.33 (m, 3H, Me *p*-cym), 1.80-1.74 (m, 2H, $H^{2''}$), 1.67-1.52 (m, 6H, $H^{3''}$ + $H^{3''',5'''}$), 1.41-1.47 (m, 2H, $H^{4'''}$), 1.38 (d J_{HH} = 6.9 Hz, 3H, Me ^{i}Pr *p*-cym), 1.36 (d, J_{HH} = 6.9 Hz, 3H, Me ^{i}Pr *p*-cym). ^{13}C{^{1}H} NMR (101 MHz, $CDCl_3$): δ 177.97 (C^4), 167.66 (C^5 or C^7 or C^2), 165.32 (C^5 or C^7 or C^2), 160.92 (C^5 or C^7 or C^2), 158.47 (C^9), 131.43 ($C^{1'}$ or $C^{4'}$), 131.16 ($C^{1'}$ or $C^{4'}$), 128.92 ($C^{3',5'}$), 126.06 ($C^{2',6'}$), 106.79 (C^3 or C^{10}), 104.52 (C^3 or C^{10}), 101.04 (C^6), 99.48 (C^1 *p*-cym), 96.82 (C^4 *p*-cym), 90.43 (C^8), 82.38, 82.32 (2 x s, $C^{2,6}$

p-cym), 78.81,78.77 (2 x s, $C^{3,5}$ *p*-cym), 67.66 ($C^{1''}$), 58.77 ($C^{4''}$), 54.39 ($C^{2''',6'''}$), 30.81 (CH ^{i}Pr *p*-cym), 27.19 ($C^{2''}$), 25.87 ($C^{3''}$), 24.39 ($C^{3''',5'''}$), 23.29 ($C^{4'''}$), 22.43, 22.39 (2 x s, 2 x Me ^{i}Pr *p*-cym), 18.05 (CH_3, *p*-cim). IR: selected band, 1634 cm^{-1} ν(C=O). MS (FAB+): m/z (%) 628 (7) $[M-Cl]^+$.

Synthesis of [RhCl(η⁵-Cp*)L1], L1-Rh. The method was similar to that described for **L1-Ru**. Amounts were as follows: 0.096 g (0.340 mmol) of **HL1**, 0.029 g (0.345 mmol) of $NaHCO_3$ and 30 mL of MeOH, in the first step of the reaction and 0.100 mg (0.162 mmol) of $[RhCl_2(\eta^5\text{-Cp*})]_2$ and 30 mL of CH_2Cl_2 in the second step. The purification was similar to that of **L1-Ru**. **L1-Rh** was obtained as a dark orange-garnet microcrystalline solid (0.142 g). Yield = 79 %. Anal. Calcd for $C_{27}H_{28}ClO_4Rh{\cdot}0.2CH_2Cl_2$ (%): C 57.13, H 5.01, Found: C 57.33, H 4.96. CH_2Cl_2 was observed in the ^{1}H NMR spectrum. ^{1}H RMN (500 MHz, $CDCl_3$, 298 K): δ 7.84 (m, 2H, $H^{2',6'}$), 7.52 – 7.45 (m, 3H, $H^{3',4',5'}$), 6.74 (s, 1H, H^3), 6.40 (d, $^4J_{HH}$ = 2.5 Hz, 1H, H^6), 6.14 (d, $^4J_{HH}$ = 2.5 Hz, 1H, H^8), 4.07 (m, 2H, O*CH*₂), 1.69 (s, 15 H, 5 x Me Cp*), 1.40 (t, $^3J_{HH}$ = 7.0 Hz, 3H, OCH_2CH_3). $^{13}C\{^1H\}$ RMN (126 MHz, $CDCl_3$, 298 K): δ 178.16 (C^4), 168.63 (C^5 or C^7 or C^2), 165.24 (C^5 or C^7 or C^2), 160.86 (C^5 or C^7 or C^2), 158.92 (C^9), 131.52 ($C^{1'}$ or $C^{4'}$), 131.40 ($C^{1'}$ or $C^{4'}$), 129.03 ($C^{3',5'}$), 126.16 ($C^{2',6'}$), 107.57 (C^3 or C^{10}), 105.72 (C^3 or C^{10}), 101.93 (C^6), 91.79 (d, $J_{Rh\text{-}C}$ = 9.5 Hz, C (Cp*)), 90.30 (C^8), 63.61 (OCH_2), 14.85 (OCH_2CH_3), 8.68 (CH_3 Cp*). IR: selected band, 1632 cm^{-1} (C=O). MS (FAB+): m/z (%) 519 (100) $[M-Cl]^+$.

Synthesis of [RhCl(η⁵-Cp*)L2], L2-Rh. The method was similar to that described for **L1-Ru**. Amounts were as follows: 0.211g (0.680 mmol) of **HL2**, 0.057 g (0.678 mmol) of $NaHCO_3$ and 60 mL of MeOH, in the first step of the reaction and 0.200 mg (0.324 mmol) of $[RhCl_2(\eta^5\text{-Cp*})]_2$ and 60 mL of CH_2Cl_2 in the second step. The purification is similar to that of **L1-Ru** but the mixture of solvents for the washings is acetone/hexane 1:4. **L2-Rh** is obtained as a dark orange- granet microcrystalline solid (0.287 g). Yield = 76 %. Anal. Calcd for $C_{29}H_{32}ClO_4Rh{\cdot}0.2CH_2Cl_2$ (%): C 58.46, H 5.44. Found: C 58.71, H 5.24. ^{1}H RMN (500 MHz, $CDCl_3$, 298 K): δ 7.84 (m, 2H, $H^{2',6'}$), 7.52 – 7.44 (m, 3H, $H^{3',4',5'}$), 6.73 (s, 1H, H^3), 6.40 (d, $^4J_{HH}$ = 2.4 Hz, 1H, H^6), 6.14 (d, $^4J_{HH}$ = 2.4 Hz, 1H, H^8), 3.99 (t, $^3J_{HH}$ = 6.5 Hz 2H, $H^{1''}$), 1.75 (q, J_{HH} = 6.5, 7.4 Hz, 2H, $H^{2''}$), 1.47 (sex, $^3J_{HH}$ = 7.4, 2H, $H^{3''}$), 1.68 (s, *15* H, 5 x Me Cp*), 0.97 (t, $^3J_{HH}$ = 7.4 Hz, 3H, $H^{4''}$). $^{13}C\{^1H\}$ RMN (126 MHz, $CDCl_3$, 298 K): δ 178.16 (C^4), 168.65 (C^5 or C^7 or C^2), 165.48 (C^5 or C^7 or C^2), 160.84 (C^5 or C^7 or C^2), 158.93 (C^9), 131.56 ($C^{1'}$ or $C^{4'}$), 131.40 ($C^{1'}$ or $C^{4'}$), 129.05 ($C^{3',5'}$), 126.18 ($C^{2',6'}$), 107.55 (C^3 or C^{10}), 105.73 (C^3 or C^{10}), 101.97 (C^6), 91.79 (d, $J_{Rh\text{-}C}$ = 9.5 Hz, C Cp*), 90.34 (C^8), 67.81 ($C^{1''}$), 31.71 ($C^{2''}$), 19.32 ($C^{3''}$), 13.91 ($C^{4''}$), 8.70 (CH_3 Cp*). IR: selected band, 1628 cm^{-1} ν(C=O). MS (FAB+): m/z (%) 547 (100) $[M-Cl]^+$.

Synthesis of [RhCl(η⁵-Cp*)L3], L3-Rh. The method was similar to that described for **L1-Ru**. Amounts were as follows: 0.132 g (0.340 mmol) of **HL3**, 0.029 g (0.345 mmol) of $NaHCO_3$ and 30 mL of MeOH, in the first

step of the reaction and 0.100 mg (0.162 mmol) of [$RhCl_2$(η^5-Cp*)]$_2$ and 30 mL of CH_2Cl_2 in the second step. The purification was similar to that of **L1-Ru** but the mixture of solvents for the washings is acetone/hexane 1:2. **L3-Rh** was obtained as a dark orange- granet microcrystalline solid (0.148 g). Yield = 69 %. Anal. Calcd. for $C_{29}H_{31}BrClO_4Rh{\cdot}0.8CH_2Cl_2$ (%): C 49.05, H 4.50 Found: C 49.16, H 4.26. CH_2Cl_2 was observed in the ^{1}H NMR spectrum. ^{1}H RMN (500 MHz, $CDCl_3$, 298 K): δ 7.83 (m, 2H, $H^{2',6'}$), 7.53 – 7.45 (m, 3H, $H^{3',4',5'}$), 6.74 (s, 1H, H^3), 6.39 (d, $^4J_{HH}$ = 2.4 Hz, 1H, H^6), 6.14 (d, $^4J_{HH}$ = 2.4 Hz, 1H, H^8), 4.03 (d, $^3J_{HH}$ = 6.0 Hz 2H, $H^{1''}$), 3.48 (d, $^3J_{HH}$ = 6.6 Hz 2H, $H^{4''}$), 2.05 (m, 2H, $H^{3''}$), 1.94 (m 2H, $H^{2''}$),1.69 (s, 15 H, 5 x Me Cp*). ^{13}C{^{1}H} RMN (126 MHz, $CDCl_3$, 298 K): δ 178.25 (C^4), 168.73 (C^5 or C^7 or C^2), 165.09 (C^5 or C^7 or C^2), 160.93 (C^5 or C^7 or C^2), 158.96 (C^9), 131.51 ($C^{1'}$ or $C^{4'}$), 131.45 ($C^{1'}$ or $C^{4'}$), 129.06 ($C^{3',5'}$), 126.19 ($C^{2',6'}$), 107.72 (C^3 or C^{10}), 105.78 (C^3 or C^{10}), 101.90 (C^6), 91.83 (d, $J_{Rh\text{-}C}$ = 9.6 Hz, C Cp*), 90.25 (C^8), 66.92 ($C^{1''}$), 33.53 ($C^{4''}$), 29.55 ($C^{3''}$), 27.83 ($C^{2''}$), 8.71 (CH_3 Cp*). IR: selected band, 1634 cm^{-1} (C=O). MS (FAB+): m/z (%) 625 (100) [M-Cl]$^+$.

Synthesis of [RhCl(η^5-Cp*)L4], L4-Rh. The method was similar to that described for **L4-Ru**. Amounts are as follows: Ligand **HL4** (0.050 g, 0.127 mmol) and $NaHCO_3$ (0.012 g, 0.1334 mmol) in 20 mL of MeOH and [Rh(η^5-C_5Me_5)Cl_2]$_2$ (0.039 g, 0.06342 mmol) in 15 mL of CH_2Cl_2. **L4-Rh** was obtained as an orange microcrystalline solid (0.051 g). Yield: 60.7%. Anal. Calcd. for $C_{34}H_{41}ClNO_4Rh$: C, 61.31; H, 6.20; N, 2.10. Found: C, 60.89, H, 5.95; N, 1.88. ^{1}H NMR (400 MHz, $CDCl_3$): δ 7.83 (d, *J* = 8.0 Hz, 2H, $H^{2',6'}$), 7.63 – 7.39 (m, 3H, $H^{3',4',5'}$), 6.73 (s, 1H, H^3), 6.39 (d, *J* = 2.4 Hz, 1H, H^6), 6.13 (d, *J* = 2.4 Hz, 1H, H^8), 4.00 (t, *J* = 6.4 Hz, 2H, $H^{1''}$), 2.35 (m, 6H, m, 6H, $H^{4''}$ + $H^{2''',6'''}$), 1.77 (m, 2H, $H^{2''}$), 1.71 (s, 15H, 5 x Me Cp*), 1.70-1.55 (m, 6H, $H^{3''}$ + $H^{3''',5'''}$), 1.44 (d, *J* = 5.3 Hz, 2H, $H^{4'''}$). ^{13}C{^{1}H} NMR (101 MHz, $CDCl_3$): δ 178.16 ($C^{4'}$), 168.63 (C^5 or C^7 or C^2), 165.38 (C^5 or C^7 or C^2), 160.86 (C^5 or C^7 or C^2), 158.92 (C^9), 131.54 ($C^{1'}$ or $C^{4'}$), 131.41 ($C^{1'}$ or $C^{4'}$), 129.05 ($C^{3',5'}$), 126.18 ($C^{2',6'}$), 107.56 (C^3 or C^{10}), 105.72 (C^3 or C^{10}), 101.94 (C^6), 91.62 (d, $J_{C\text{-}Rh}$ = 9.3 Hz, C Cp*), 90.33 (C^8), 67.91($C^{1''}$), 59.16 ($C^{4''}$), 54.74 ($C^{2''',6'''}$), 27.43 ($C^{2''}$), 26.18 ($C^{3''}$), 24.65 ($C^{3''',5'''}$), 23.60 ($C^{4'''}$), 8.70 (Me Cp*). IR: selected band, 1629 cm^{-1} ν(C=O).

Synthesis of [IrCl(η^5-Cp*)L1], L1-Ir. The method was similar to that described for **L1-Ru**. Amounts were as follows: 0.075 g (0.266 mmol) of **HL1**, 0.022 g (0.261 mmol) of $NaHCO_3$ and 30 mL of MeOH, in the first step of the reaction and 0.100 mg (0.126 mmol) of [$IrCl_2$(η^5-Cp*)]$_2$ and 30 mL of CH_2Cl_2 in the second step. The purification was similar to that of **L1-Ru. L1-Ir** was obtained as an orange microcrystalline solid (0.112 g). Yield = 76 %. Anal. Calcd for $C_{27}H_{28}ClO_4Ir{\cdot}0.2(CH_2Cl_2)$ (%): C 49.41, H 4.33 Found: C 49.22, H 4.09. CH_2Cl_2 was observed in the ^{1}H NMR spectrum. ^{1}H RMN (500 MHz, $CDCl_3$, 298 K): δ 7.86 (m, 2H, $H^{2',6'}$), 7.53 – 7.45 (m, 3H, $H^{3',4',5'}$), 6.75 (s, 1H, H^3), 6.41 (d, $^4J_{HH}$ = 2.3 Hz, 1H, H^6), 6.19 (d, $^4J_{HH}$ = 2.3 Hz, 1H, H^8), 4.07 (m, 2H, OCH_2), 1.65 (s, 15 H, 5 x Me Cp*), 1.41 (t, $^3J_{HH}$ = 7.0 Hz, 3H, OCH_2CH_3). ^{13}C{^{1}H} RMN (126 MHz, $CDCl_3$, 298

K): δ 176.54 (C^4), 167.05 (C^5 or C^7 or C^2), 165.43 (C^5 or C^7 or C^2), 161.20 (C^5 or C^7 or C^2), 158.50 (C^9), 131.54 ($C^{1'}$ or $C^{4'}$), 131.51 ($C^{1'}$ or $C^{4'}$), 129.13 ($C^{3',5'}$), 126.19 ($C^{2'6'}$), 108.01 (C^3 or C^{10}), 105.29 (C^3 or C^{10}), 101.36 (C^6), 90.94 (C^8), 83.02 (C Cp*), 63.74 (OCH_2), 14.85 (OCH_2CH_3), 8.90 (CH_3 Cp*). IR: selected band, 1630 cm^{-1} (C=O). MS (FAB+): m/z (%) 609 (100) $[M-Cl]^+$.

Synthesis of [IrCl(η⁵-Cp*)L2], L2-Ir. The method was similar to that described for **L1-Ru**. Amounts were as follows: 0.164 g (0.528 mmol) of **HL2**, 0.044 g (0.524 mmol) of $NaHCO_3$ and 60 mL of MeOH, in the first step of the reaction and 0.200 mg (0.251 mmol) of $[IrCl_2(\eta^5\text{-}Cp^*)]_2$ and 60 mL of CH_2Cl_2 in the second step. The purification was similar to that of **L1-Ru. L2-Ir** was obtained as an orange microcrystalline solid (0.236 g). Yield = 70 %. Anal. Calcd. for $C_{29}H_{32}ClO_4Ir{\cdot}0.8CH_2Cl_2$ (%): C 48.36, H 4.58 Found: C 47.96, H 4.14. CH_2Cl_2 was observed in the ^1H NMR spectrum. ^1H RMN (500 MHz, $CDCl_3$, 298 K): δ 7.85 (m, 2H, $H^{2',6'}$), 7.53 – 7.45 (m, 3H, $H^{3',4',5'}$), 6.75 (s, 1H, H^3), 6.41 (d, $^4J_{HH}$ = 2.3 Hz, 1H, H^6), 6.20 (d, $^4J_{HH}$ = 2.3 Hz, 1H, H^8), 4.00 (d, $^3J_{HH}$ = 6.5 Hz 2H, $H^{1''}$), 1.77 (m, 2H, $H^{2''}$), 1.65 (s, 15 H, 5 x Me Cp*), 1.47 (m 2H, $H^{3''}$), 0.97 (t, $^3J_{HH}$ = 7.4 Hz, 3H, $H^{4''}$). $^{13}C\{^1H\}$ RMN (126 MHz, $CDCl_3$, 298 K): δ 176.50 (C^4), 167.01 (C^5 or C^7 or C^2), 165.63 (C^5 or C^7 or C^2), 161.15 (C^5 or C^7 or C^2), 158.46 (C^9), 131.51 ($C^{1'}$ or $C^{4'}$), 131.48 ($C^{1'}$ or $C^{4'}$), 129.10 ($C^{3',5'}$), 126.15 ($C^{2',6'}$), 107.95 (C^3 or C^{10}), 105.25 (C^3 or C^{10}), 101.38 (C^6), 90.93 (C^8), 82.99 (C Cp*), 67.89 (s, $C^{1''}$), 31.20 ($C^{2''}$), 19.29 ($C^{3''}$), 13.88 (s, $C^{4''}$), 8.88 (s, CH_3 Cp*). IR: selected band, 1636 cm^{-1} ν(C=O). MS (FAB+): m/z (%) 637 (100) $[M-Cl]^+$.

Synthesis of [IrCl(η⁵-Cp*)L3], L3-Ir. The method is similar to that described for **L1-Ru**. Amounts are as follows: 0.103 g (0.265 mmol) of HL3, 0.022 g (0.262 mmol) of $NaHCO_3$ and 30 mL of MeOH, in the first step of the reaction and 0.100 mg (0.126 mmol) of $[IrCl_2(\eta^5\text{-}Cp^*)]_2$ and 30 mL of CH_2Cl_2 in the second step. The purification is similar to that of **L1-Ru** but the mixture of solvents for the washings is acetone/hexane 1:4. **L3-Ir** is obtained as an orange microcrystalline solid (0.137 g). Yield = 72 %. Anal. Calcd. for $C_{29}H_{31}BrClO_4Ir{\cdot}$· 1.25$CH_2Cl_2$ (%): C 42.38, H 3.94 Found: C 42.16, H 3.65. CH_2Cl_2 was observed in the ^1H NMR spectrum. ^1H RMN (500 MHz, $CDCl_3$, 298 K): δ 7.85 (m, 2H, $H^{2',6'}$), 7.54 – 7.46 (m, 3H, $H^{3',4',5'}$), 6.76 (s, 1H, H^3), 6.41 (d, $^4J_{HH}$ = 2.4 Hz, 1H, H^6), 6.20 (d, $^4J_{HH}$ = 2.4 Hz, 1H, H^8), 4.05 (d, $^3J_{HH}$ = 6.0 Hz 2H, $H^{1''}$), 3.48 (d, $^3J_{HH}$ = 6.5 Hz 2H, $H^{4''}$), 2.05 (m, 2H, $H^{3''}$), 1.95 (m 2H, $H^{2''}$),1.66 (s, 15 H, 5 x Me Cp*). $^{13}C\{^1H\}$ RMN (126 MHz, $CDCl_3$, 298 K): δ 176.61 (C^4), 167.11 (C^5 or C^7 or C^2), 165.24 (C^5 or C^7 or C^2), 161.24 (C^5 or C^7 or C^2), 158.49 (C^9), 131.57 ($C^{1'}$ or $C^{4'}$), 131.44 ($C^{1'}$ or $C^{4'}$), 129.13 ($C^{3',5'}$), 126.17 ($C^{2',6'}$), 108.12 (C^3 or C^{10}), 105.32 (C^3 or C^{10}), 101.34 (C^6), 90.84 (C^8), 83.03 (C Cp*), 67.02 ($C^{1''}$), 33.47 ($C^{4''}$), 29.52 ($C^{3''}$), 27.80 ($C^{2''}$), 8.89 (CH_3 Cp*). IR: selected band, 1628 cm^{-1} ν(C=O). MS (FAB+): m/z (%) 715 (43) $[M-Cl]^+$.

Synthesis of [IrCl(η^5-Cp*)L4], L4-Ir. The method was similar to that described for **L4-Ru**. Amounts were as follows: Ligand **HL4** (0.100 g, 0.254 mmol) and $NaHCO_3$ (0.022 g, 0.267 mmol) in 30 mL of MeOH and [Ir(η^5-C_5Me_5)Cl_2]$_2$ (0.1012 g, 0.1270 mmol) in 20 mL of CH_2Cl_2. **L4-Ir** was obtained as an orange microcrystalline solid (0.103 g). Yield: 54%. Anal. Calcd. for $C_{34}H_{41}ClNO_4Ir{\cdot}0.5CH_2Cl_2$ C, 51.94, H 5.31, N, 1.76. Found: C, 52.03; H, 4.84; N, 1.60. CH_2Cl_2 was observed in the ^{1}H NMR spectrum. ^{1}H NMR (400 MHz, $CDCl_3$): δ 7.94 – 7.75 (m, 2H$^{2',6'}$), 7.60 – 7.41 (m, 3H, H$^{3',4',5'}$), 6.75 (s, 1H, H^3), 6.40 (d, J = 2.4 Hz, 1H, H^6), 6.19 (d, J = 5.1 Hz, 1H, H^8), 4.01 (t, J = 6.5 Hz, 2H, H$^{1''}$), 2.46 – 2.29 (m, 6H, H$^{4''}$+ H$^{2''',6'''}$), 1.82 – 1.72 (m, 4H, H$^{2'',3''}$), 1.67 (s, 15H, Me Cp*), 1.58 (m, 4H, H$^{3''',5'''}$), 1.43 (m, 2H, H$^{4'''}$). ^{13}C{^{1}H} NMR (101 MHz, $CDCl_3$): δ 176.50 (C^4), 167.0 (C^5 or C^7 or C^2), 165.51 (C^5 or C^7 or C^2), 161.16 (C^5 or C^7 or C^2), 158.45 (C^9), 131.38 (C$^{1'}$ or C$^{4'}$), 131.46 (C$^{1'}$ or C$^{4'}$), 129.18 (C$^{3',5'}$), 126.37 (C$^{2',6'}$), 107.97 (C^3 or C^{10}), 105.25 (C^3 or C^{10}), 101.36 (C^6), 90.91 (C^8) , 82.99 (C Cp*), 67.97(C$^{1''}$), 59.07 (C$^{4''}$), 54.68 (C$^{2''',6'''}$), 27.35 (C$^{2''}$), 26.07 (C$^{3''}$), 24.56 (C$^{3''',5'''}$), 23.49 (C$^{4'''}$), 8.87 (Me Cp*). IR: selected band, 1627 cm^{-1} ν(C=O). MS (FAB+): m/z (%) 720 (37) [M-Cl]$^+$.

^{1}H and ^{13}C{^{1}H} NMR spectra of **HL1-HL4** and **M-L** (M = Ru, Rh, Ir; **L** = **L1**, **L2**, **L3**, **L4**) complexes. Some bidimensional spectra are also included.

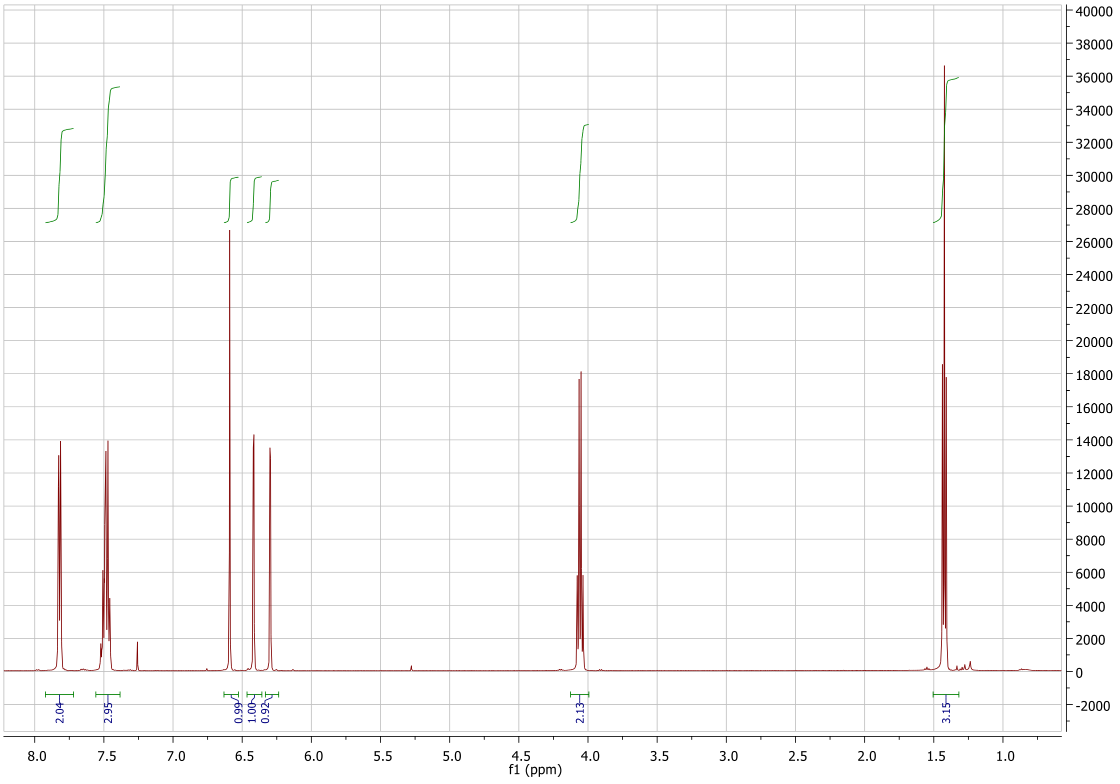

^{1}H-NMR of **HL1** in $CDCl_3$ (298 K, 500 MHz)

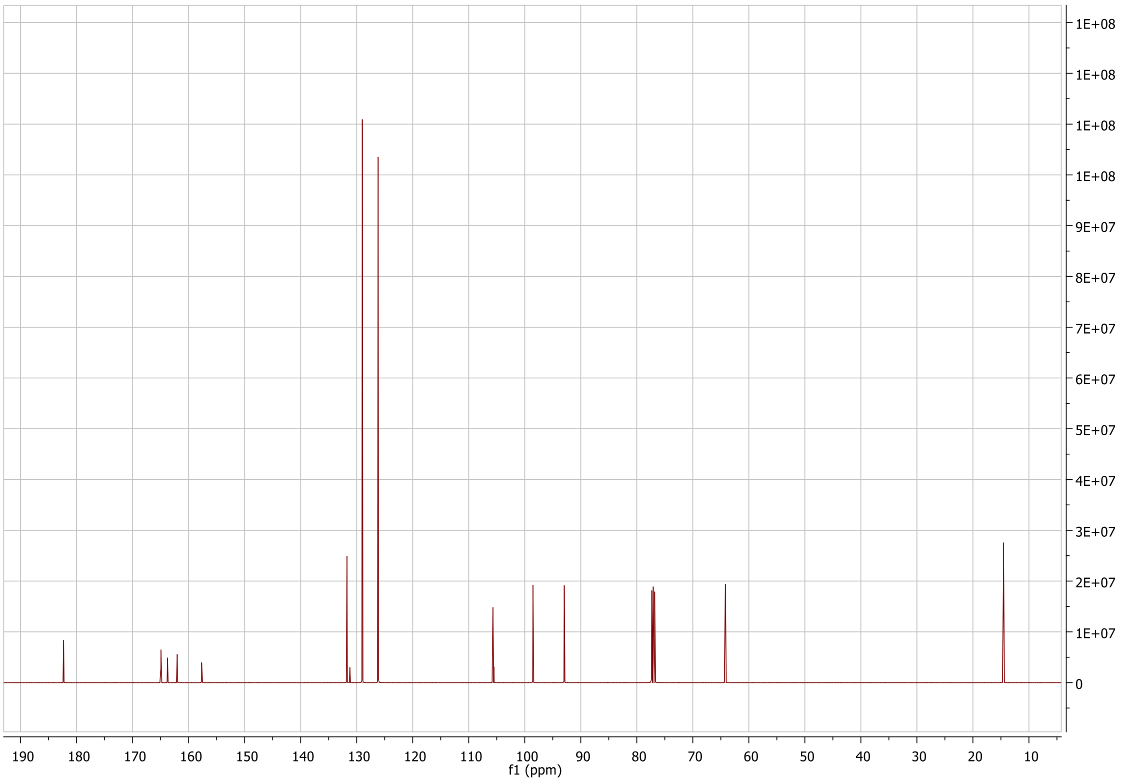

$^{13}C\{^{1}H\}$-NMR of **HL1** in $CDCl_3$ (298 K, 126 MHz)

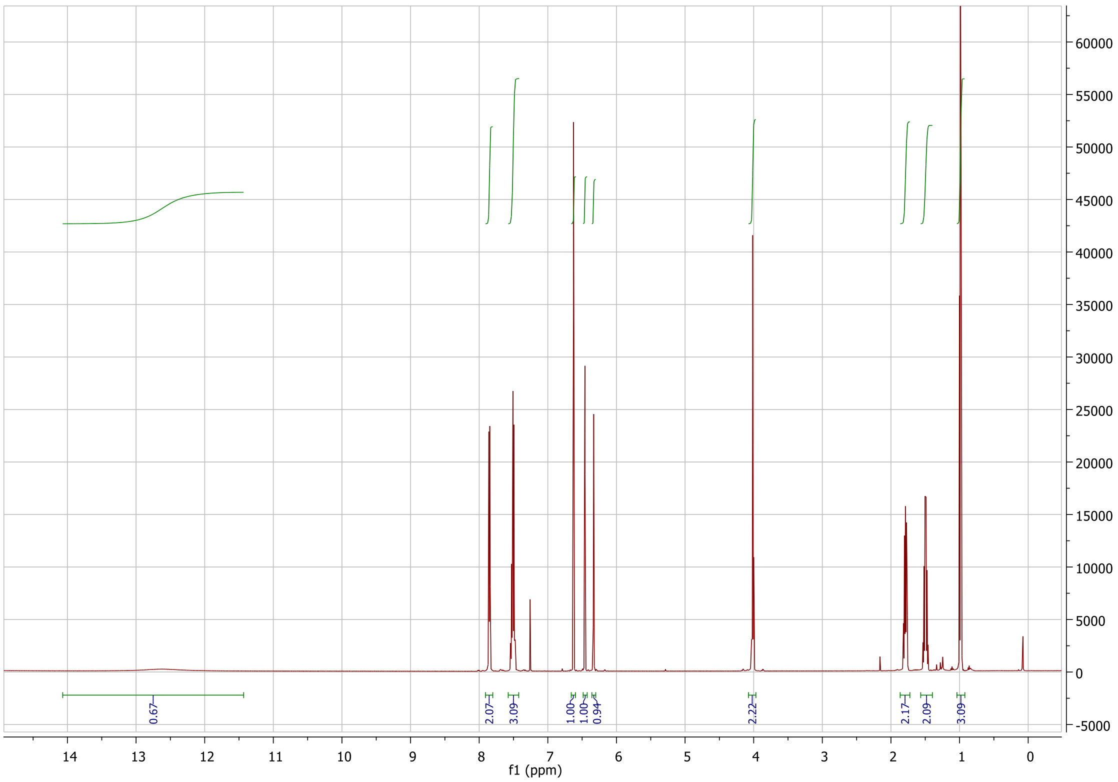

^{1}H-NMR of **HL2** in $CDCl_3$ (298 K, 500 MHz)

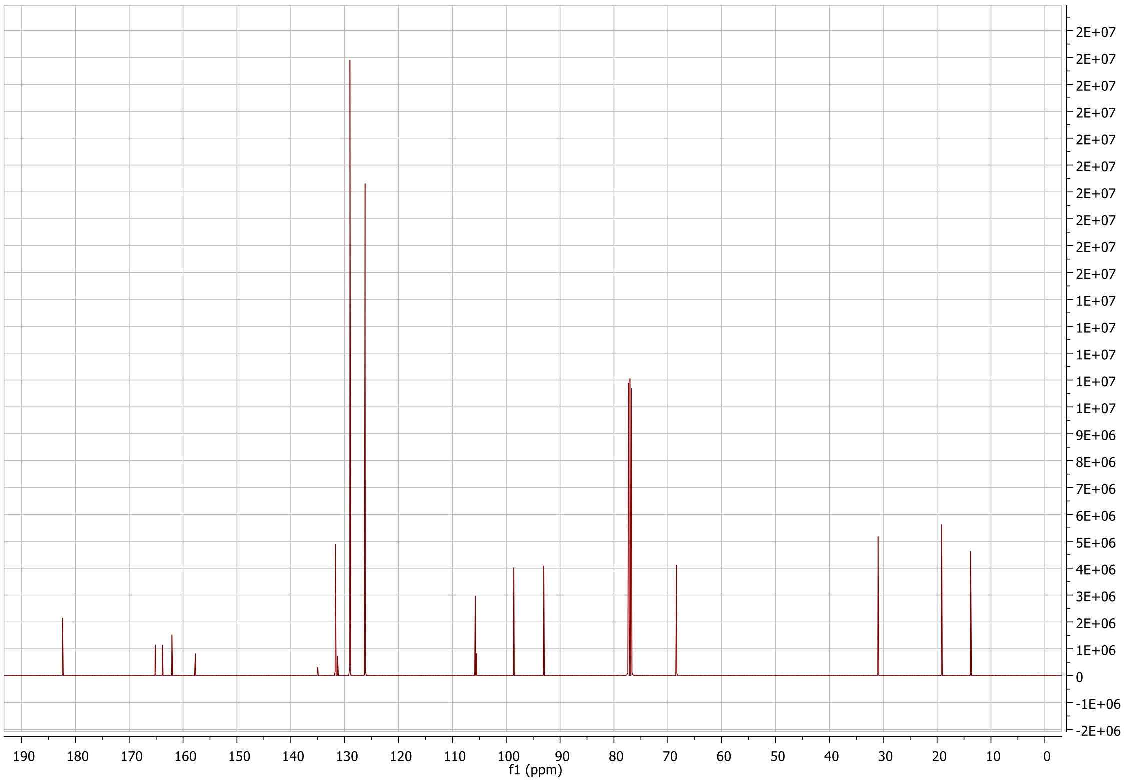

^{13}C{^{1}H}-NMR of **HL2** in $CDCl_3$ (298 K, 126 MHz)

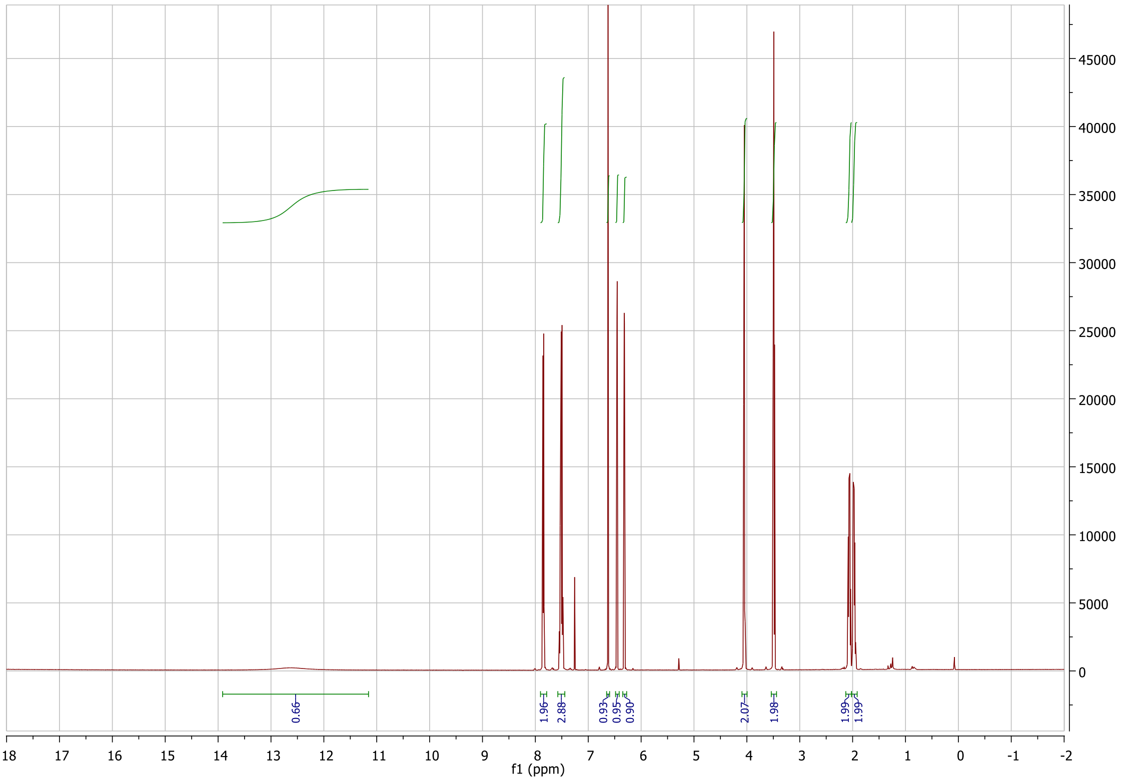

^{1}H-NMR of **HL3** in $CDCl_3$ (298 K, 500 MHz)

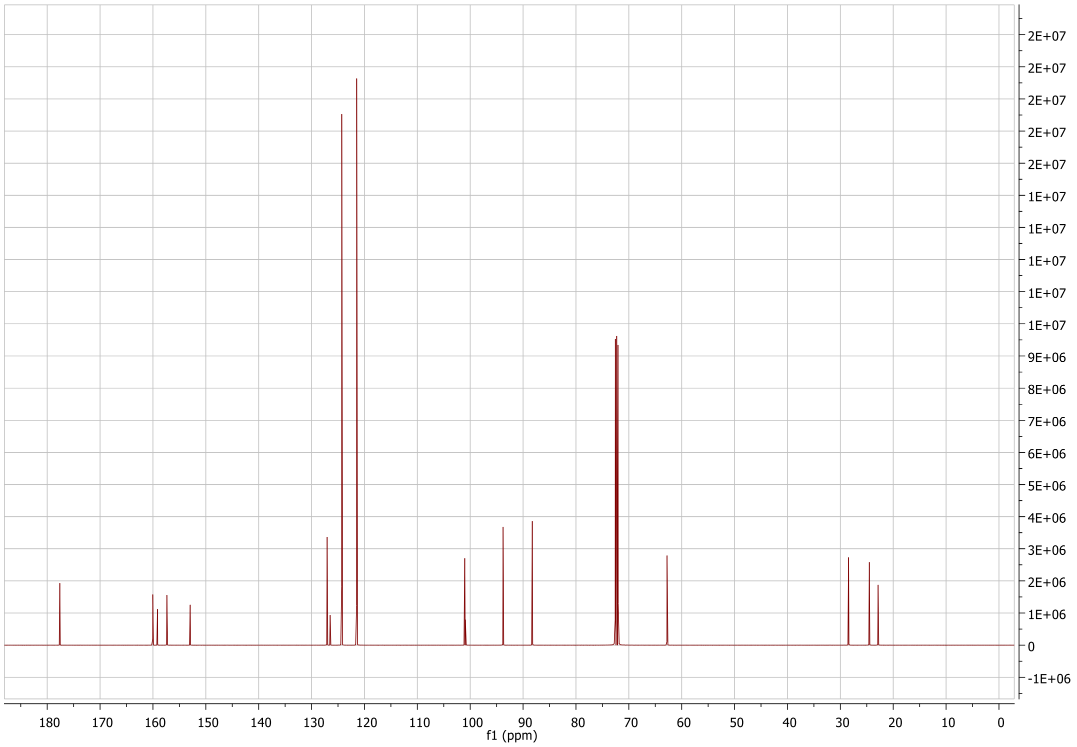

$^{13}C\{^{1}H\}$-NMR of **HL3** in $CDCl_3$ (298 K, 126 MHz)

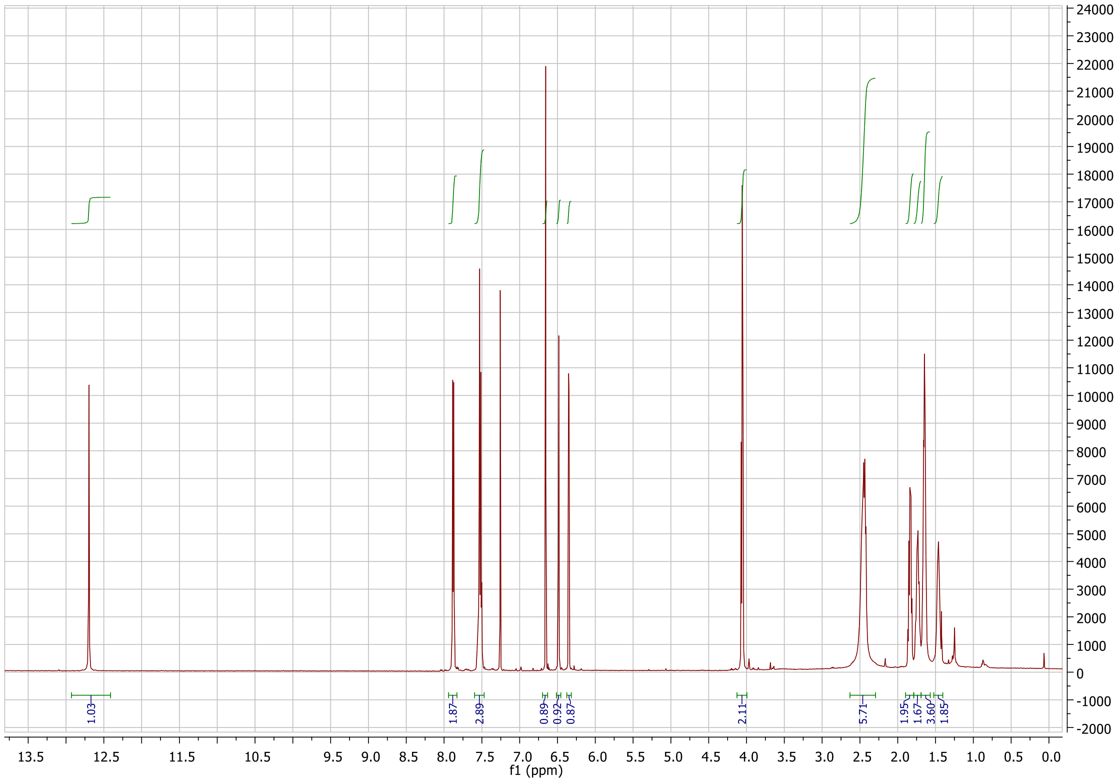

^{1}H-NMR of **HL4** in $CDCl_3$ (298 K, 500 MHz)

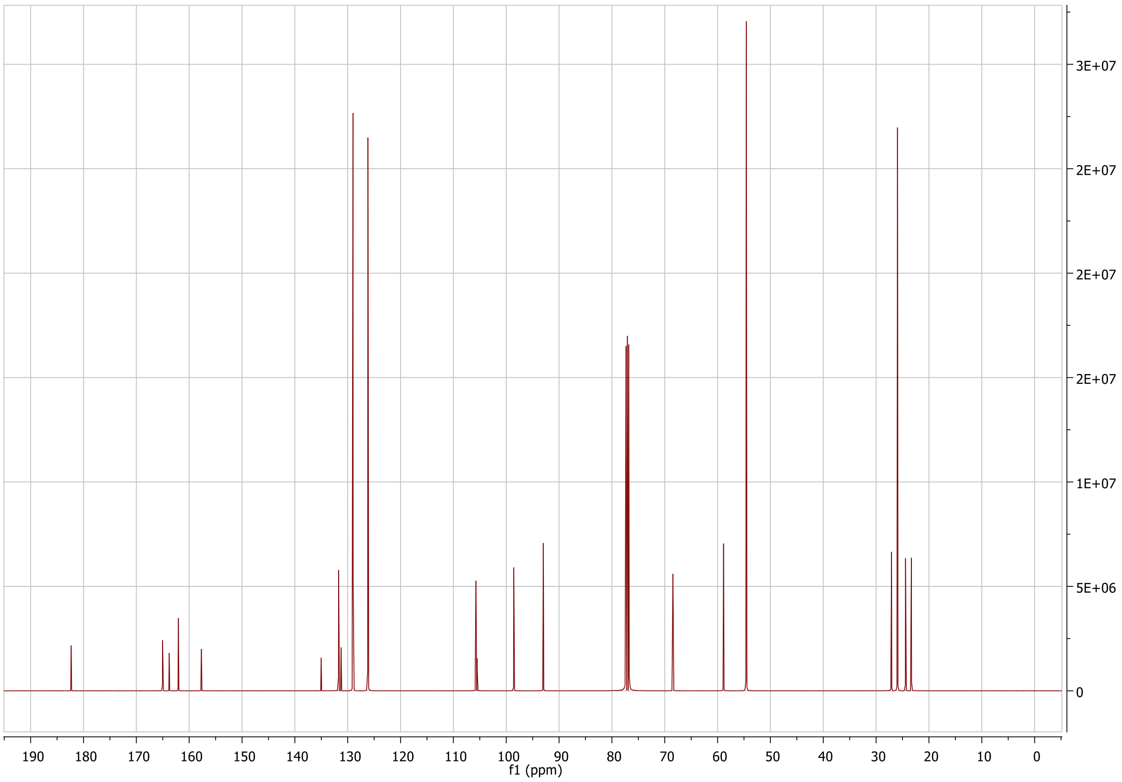

$^{13}C\{^{1}H\}$-NMR of **HL4** in $CDCl_3$ (298 K, 126 MHz)

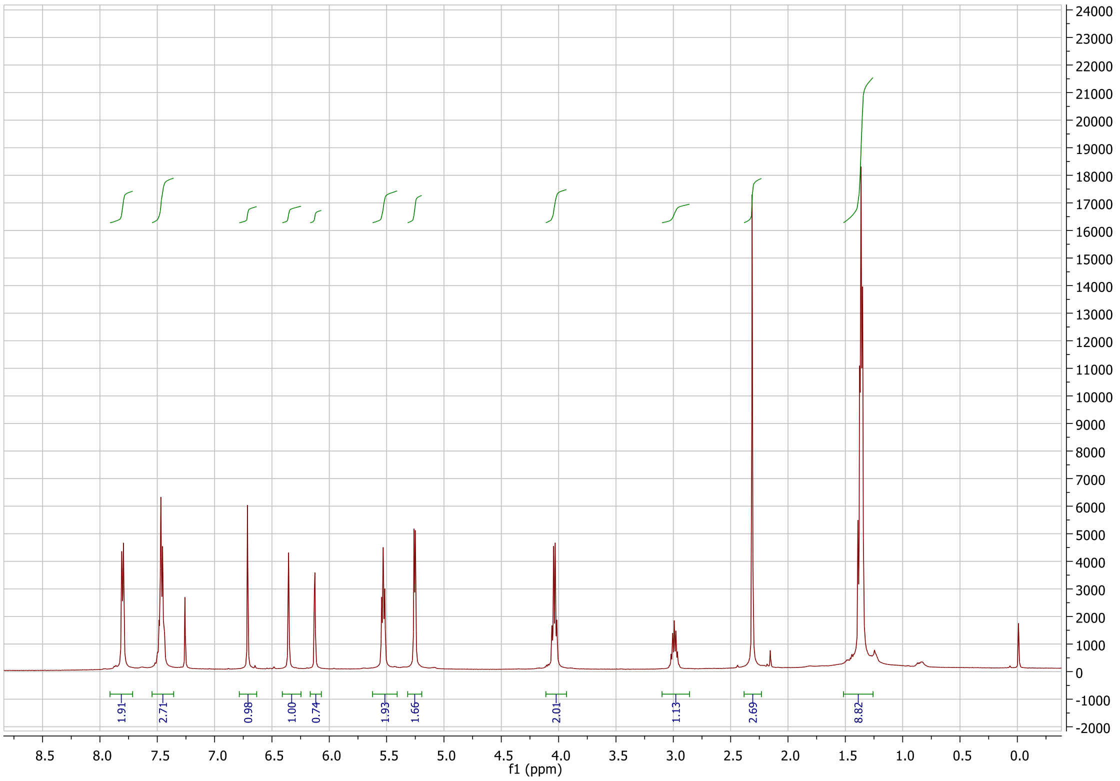

^{1}H-NMR of **L1-Ru** in $CDCl_3$ (298 K, 500 MHz)

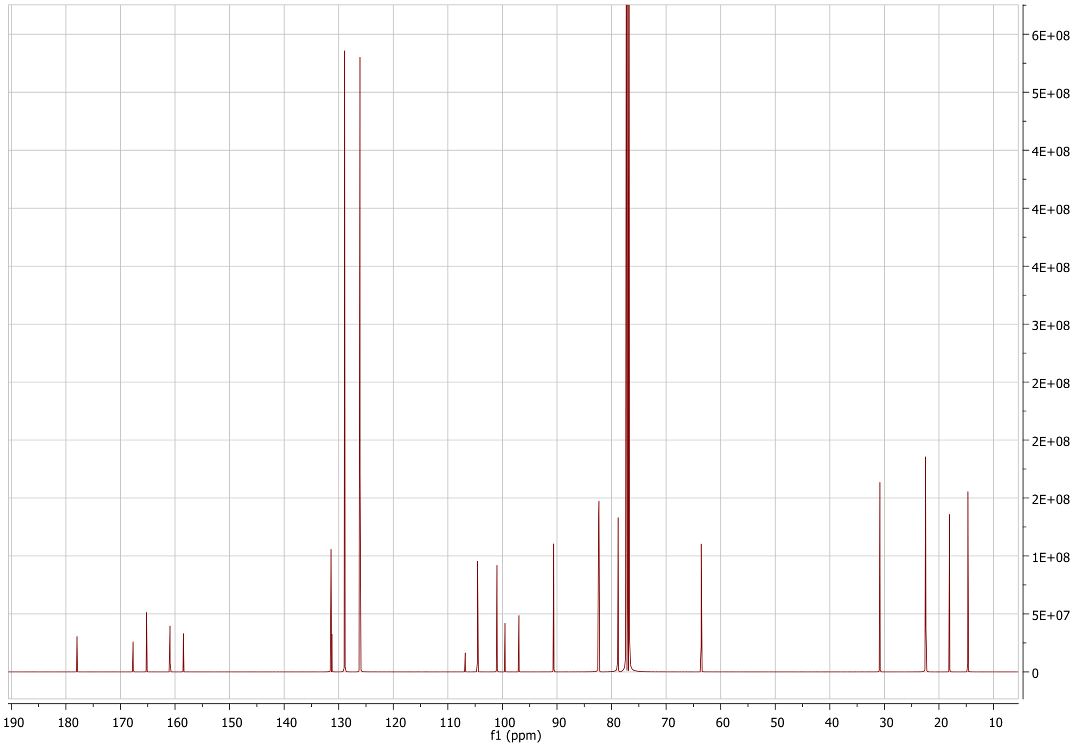

$^{13}C\{^{1}H\}$-NMR of **L1-Ru** in $CDCl_3$ (298 K, 126 MHz)

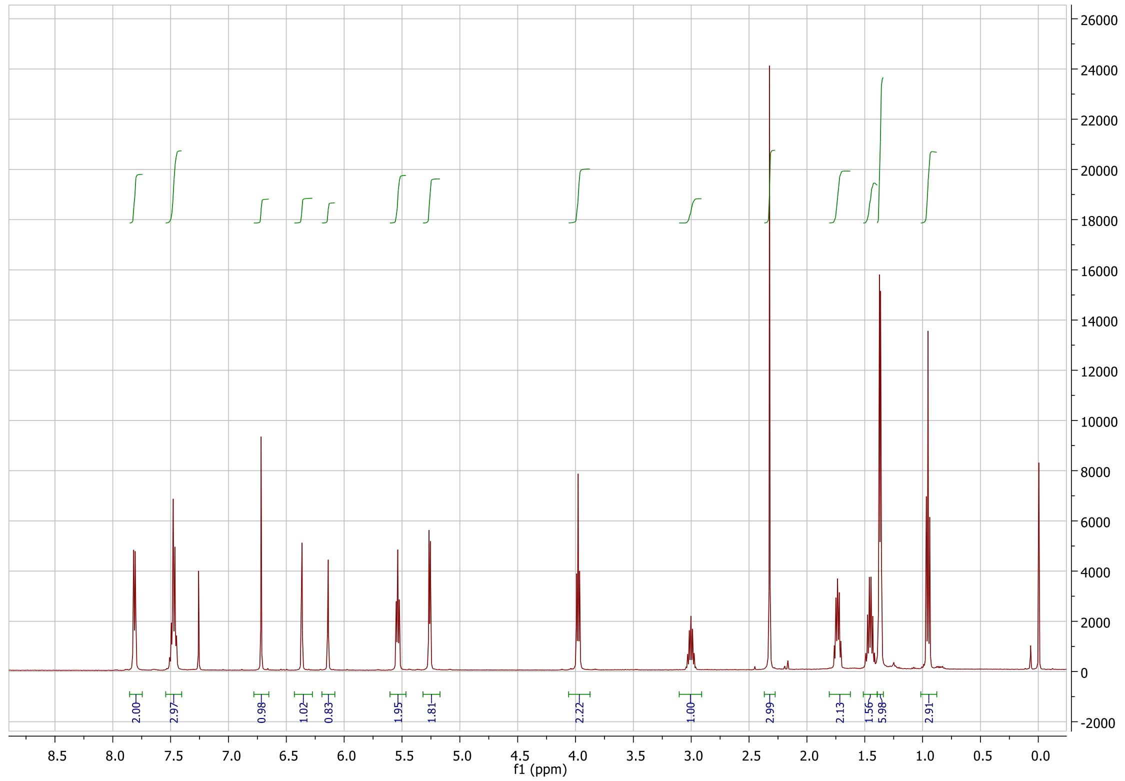

^{1}H-NMR of **L2-Ru** in $CDCl_3$ (298 K, 500 MHz)

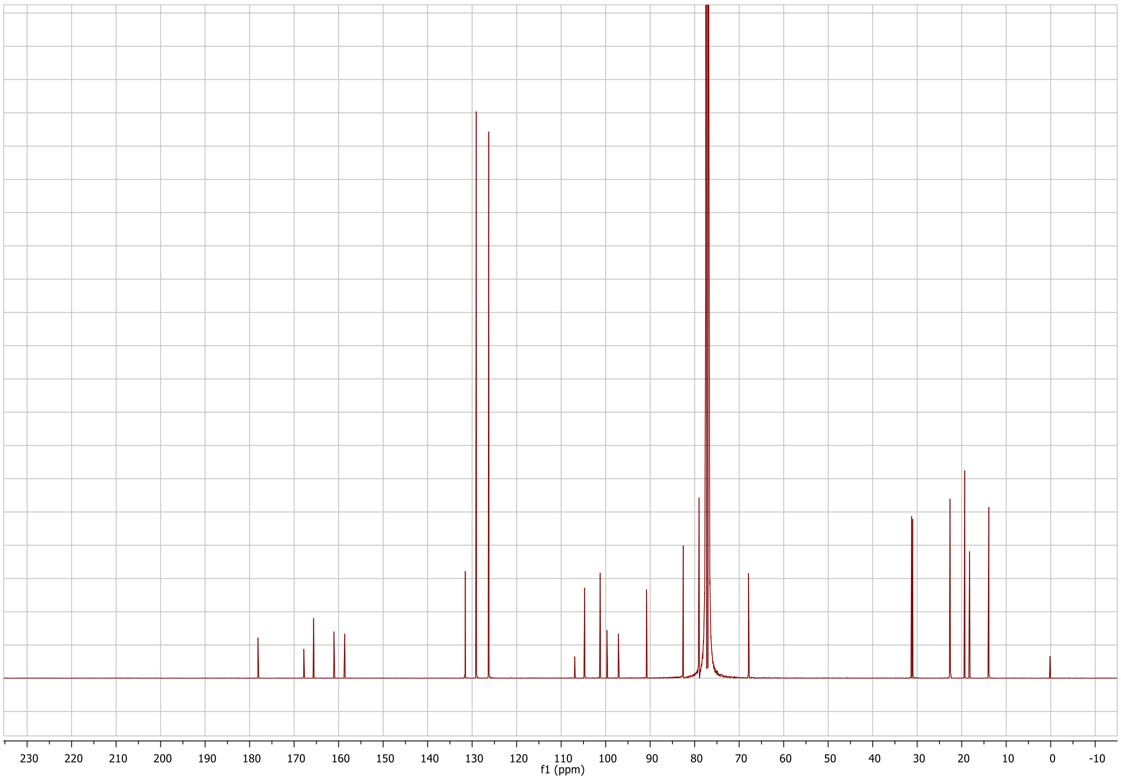

$^{13}C\{^{1}H\}$-NMR of **L2-Ru** in $CDCl_3$ (298 K, 126 MHz)

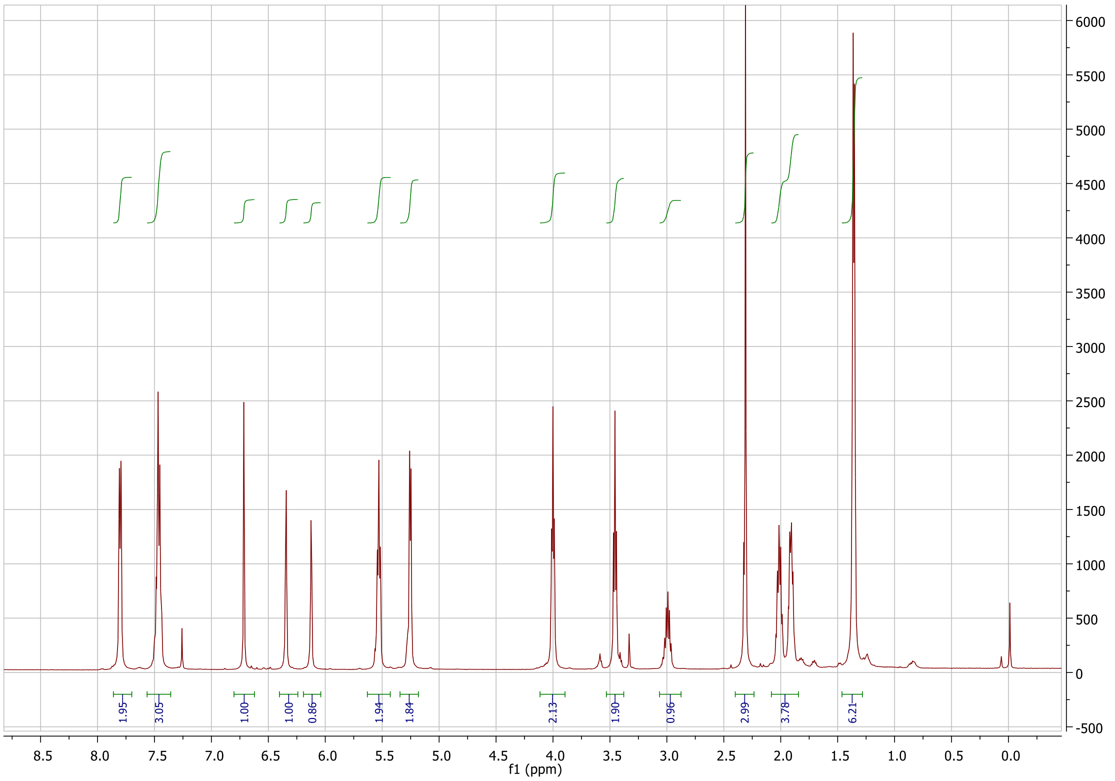

^{1}H-NMR of **L3-Ru** in $CDCl_3$ (298 K, 500 MHz)

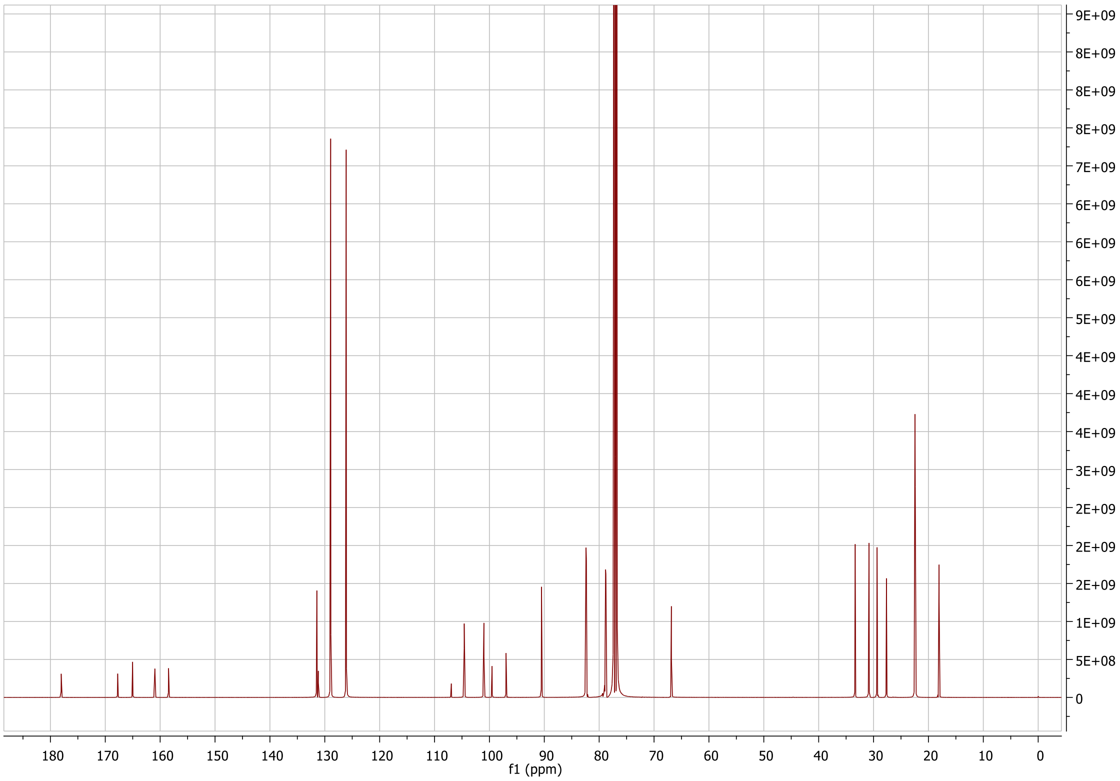

$^{13}C\{^{1}H\}$-NMR of **L3-Ru** in $CDCl_3$ (298 K, 126 MHz)

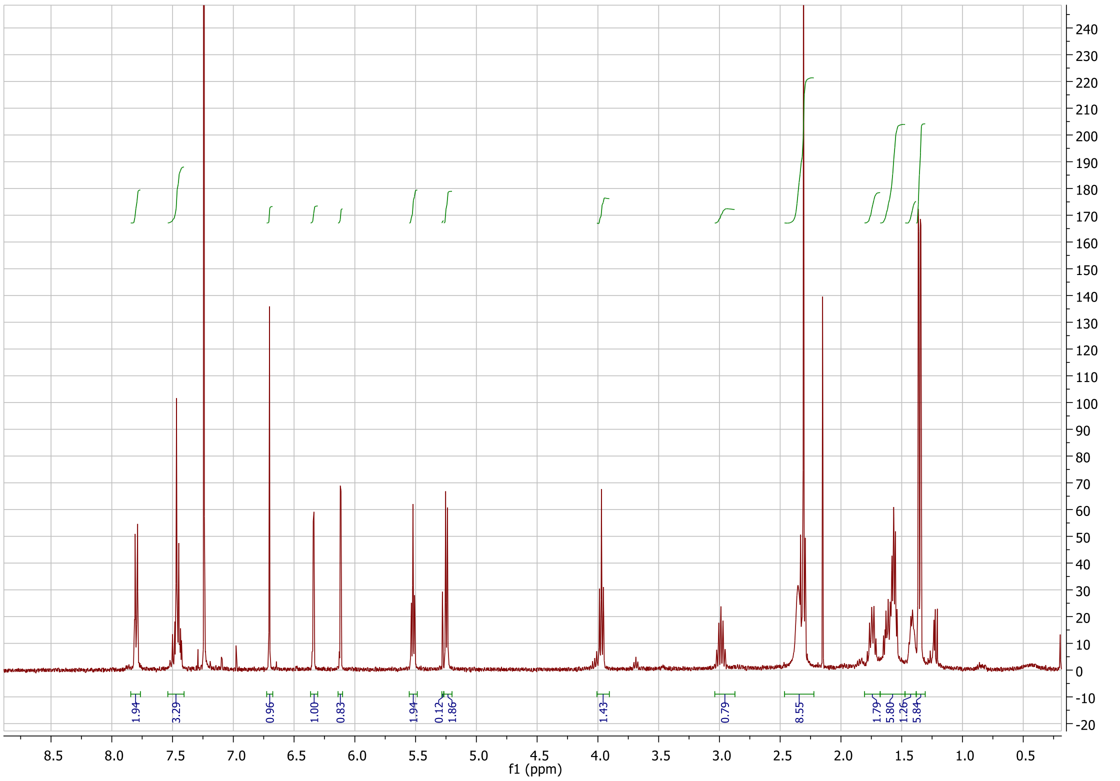

^{1}H-NMR of **L4-Ru** in $CDCl_3$ (298 K, 500 MHz)

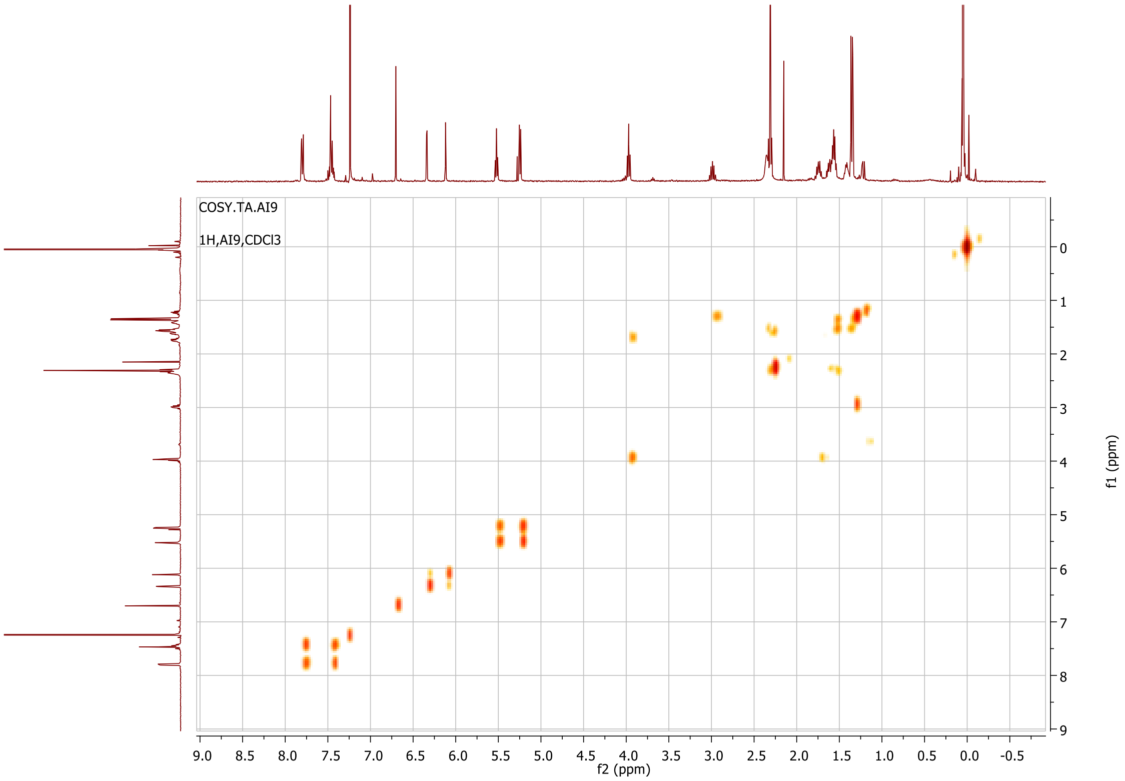

^{1}H-^{1}H COSY of **L4-Ru** in $CDCl_3$ (298 K, 500 MHz)

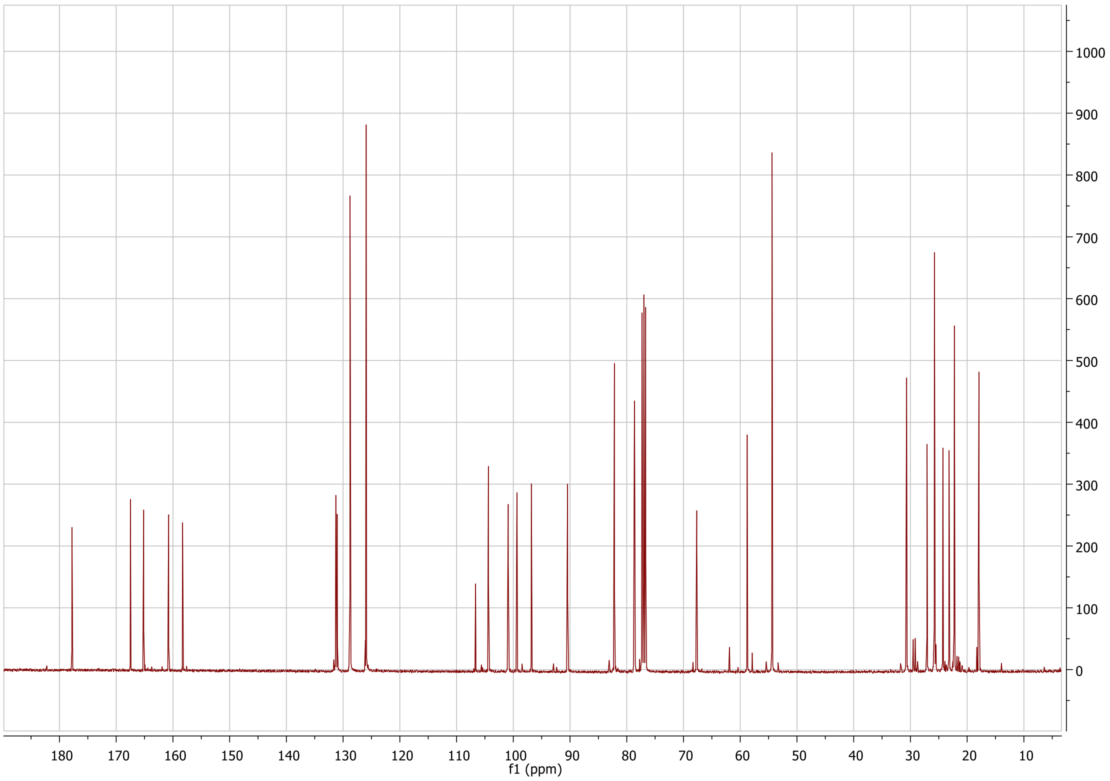

^{13}C{^{1}H}-NMR of **L4-Ru** in $CDCl_3$ (298 K, 126 MHz)

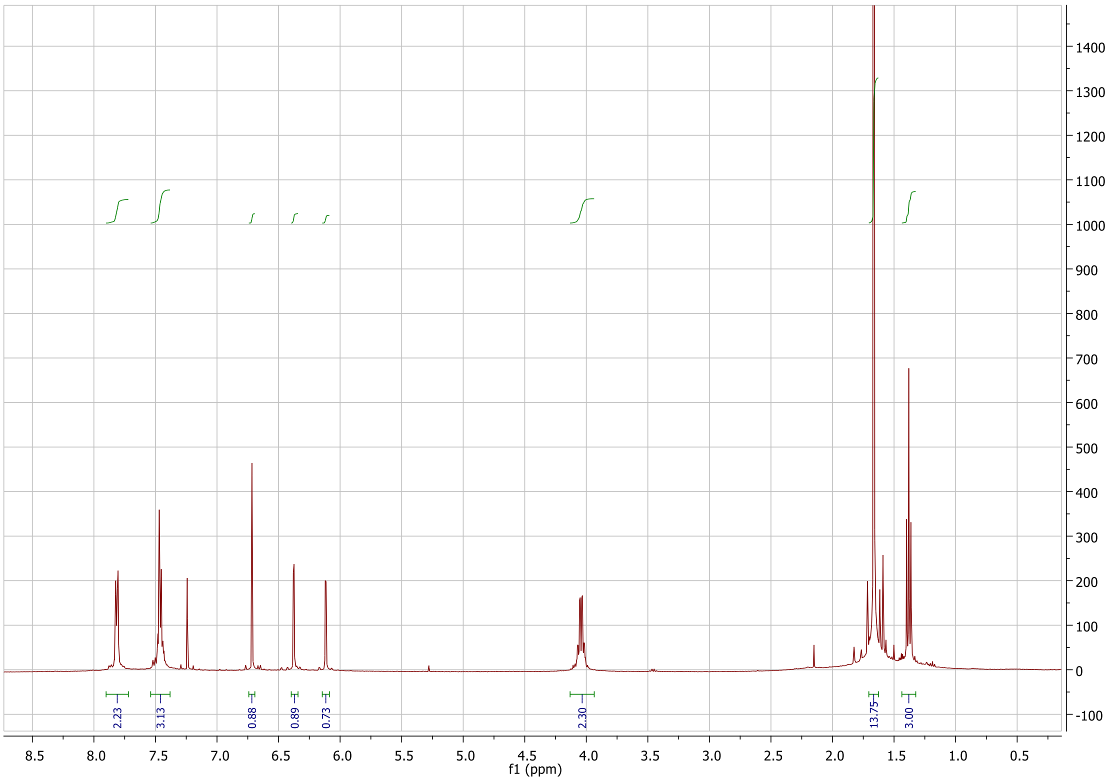

^{1}H-NMR of **L1-Rh** in $CDCl_3$ (298 K, 500 MHz)

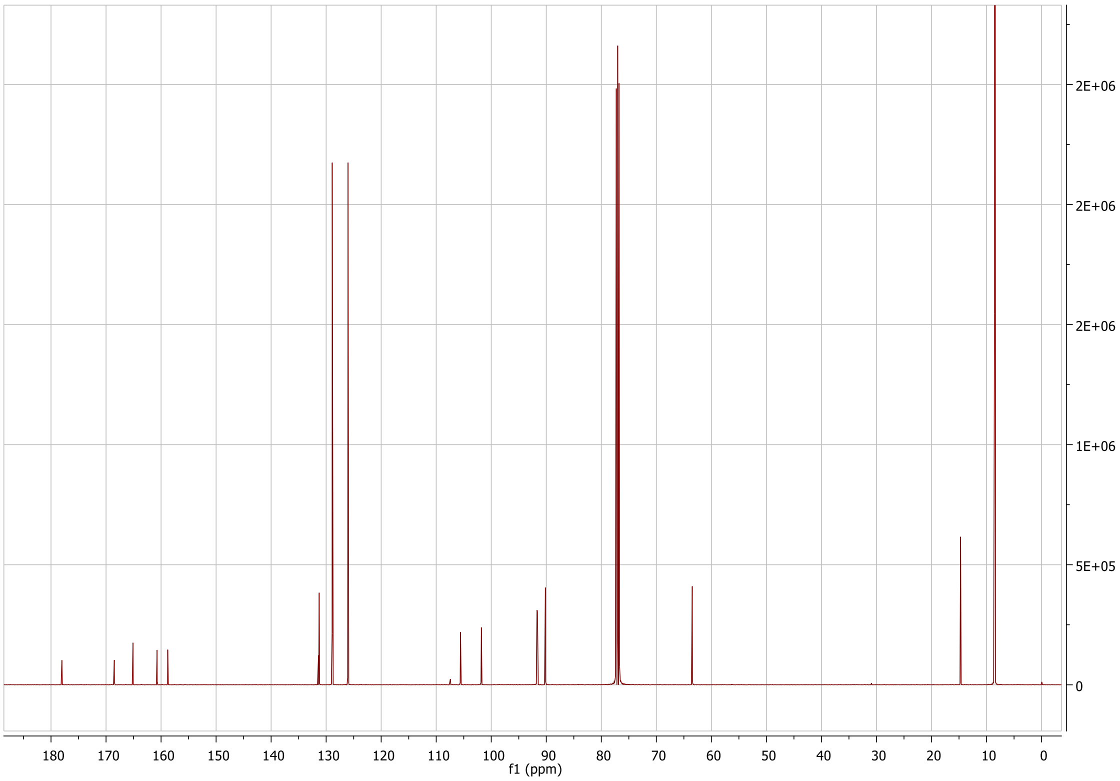

^{13}C{^{1}H}-NMR of **L1-Rh** in $CDCl_3$ (298 K, 126 MHz)

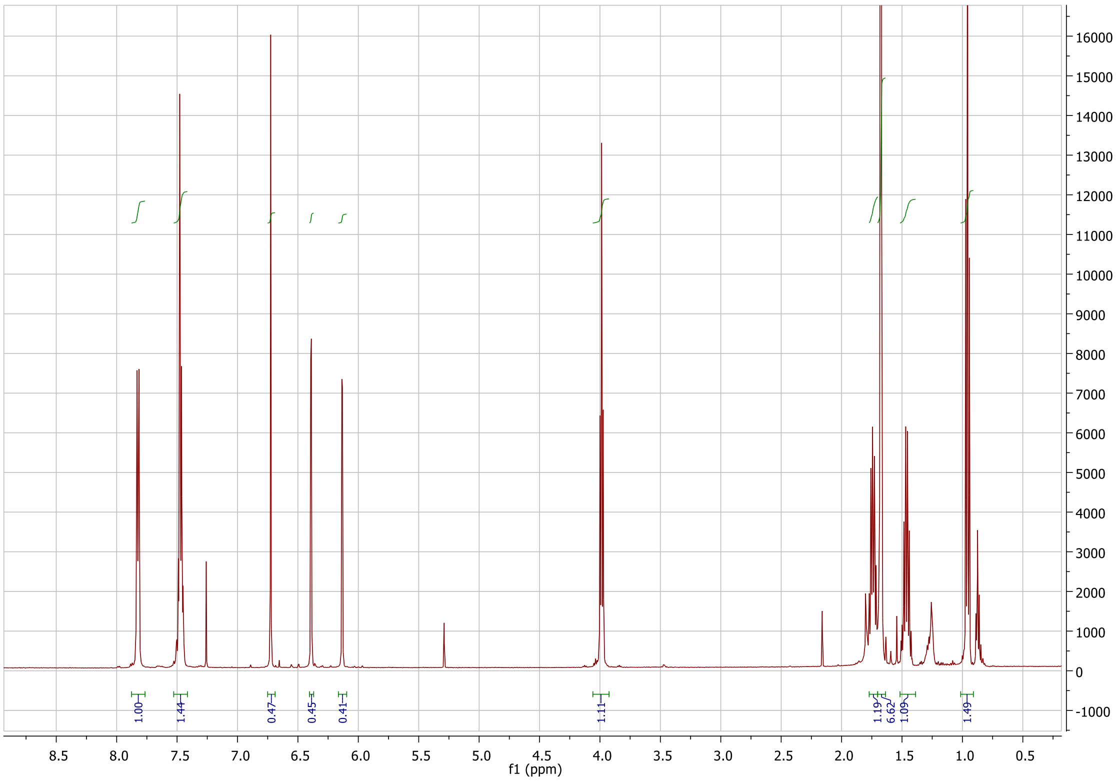

^{1}H-NMR of **L2-Rh** in $CDCl_3$ (298 K, 500 MHz)

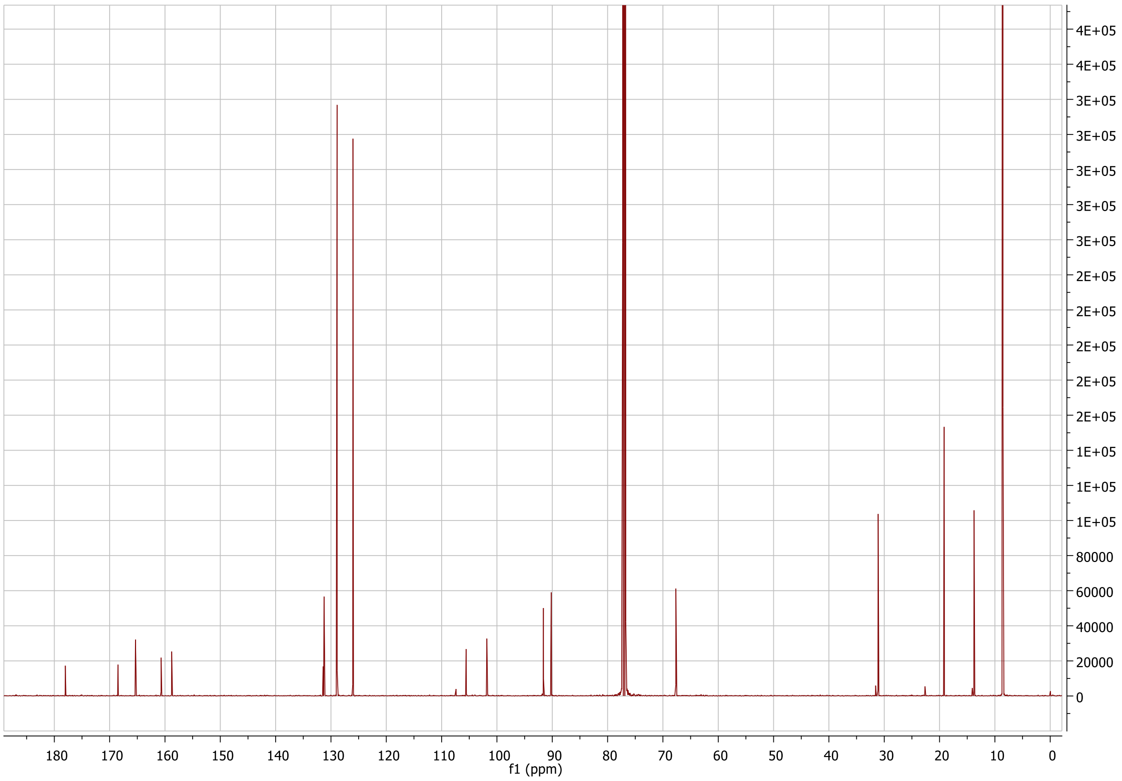

^{13}C{^{1}H}-NMR of **L2-Rh** in $CDCl_3$ (298 K, 126 MHz)

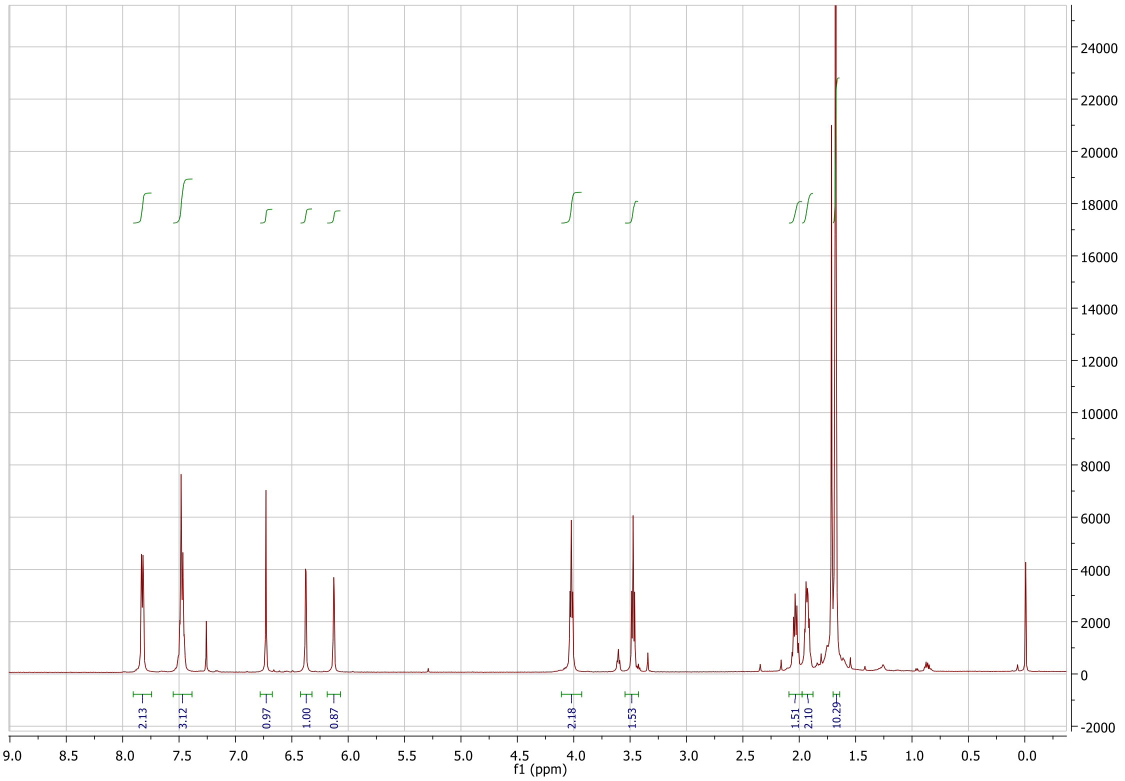

^{1}H-NMR of **L3-Rh** in $CDCl_3$ (298 K, 500 MHz)

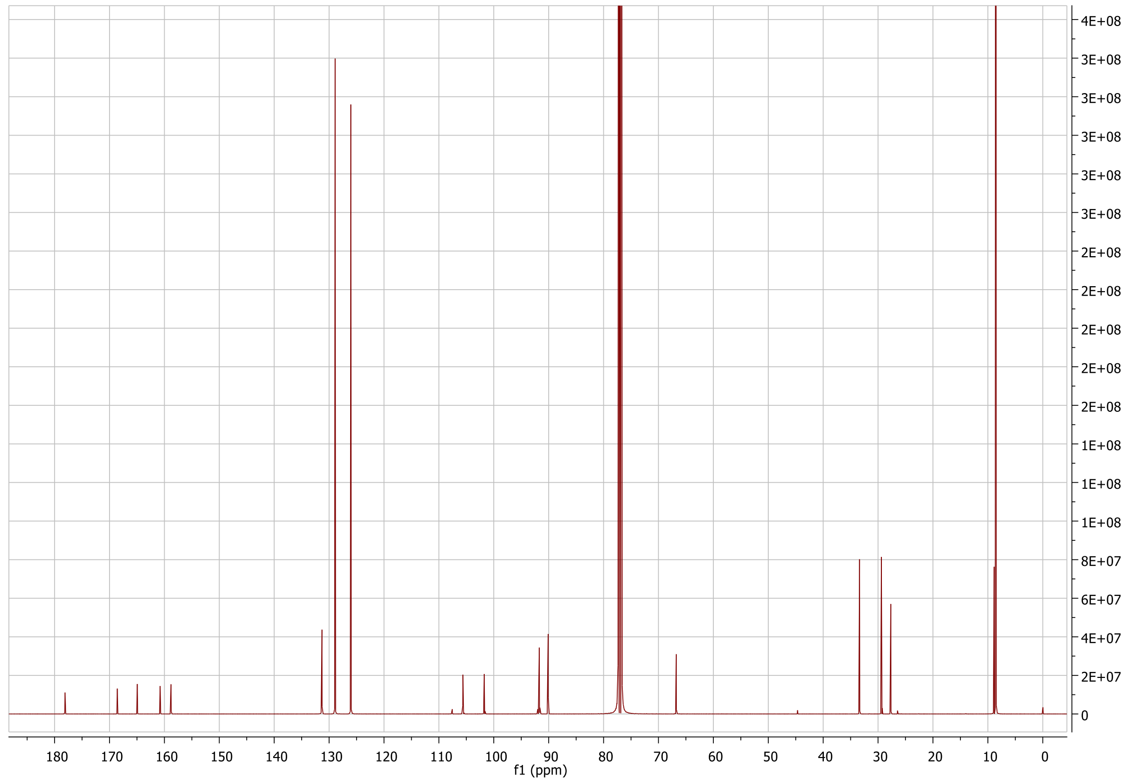

^{13}C{^{1}H}-NMR of **L3-Rh** in $CDCl_3$ (298 K, 126 MHz)

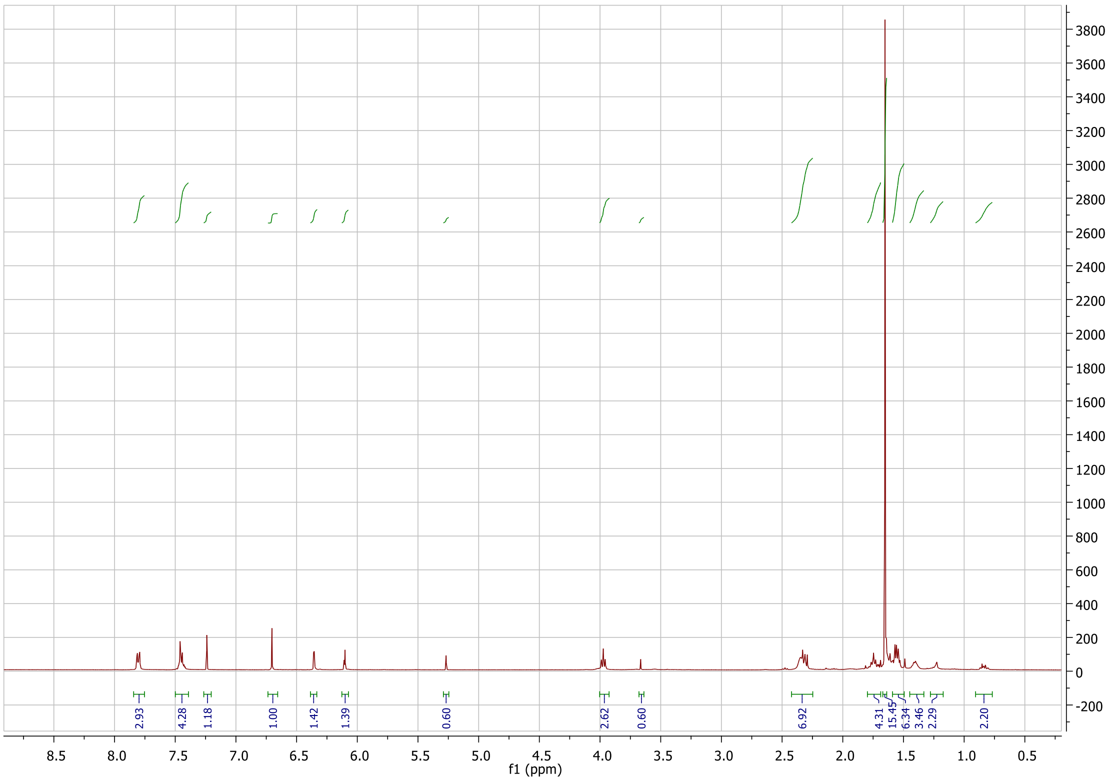

^{1}H-NMR of **L4-Rh** in $CDCl_3$ (298 K, 400 MHz)

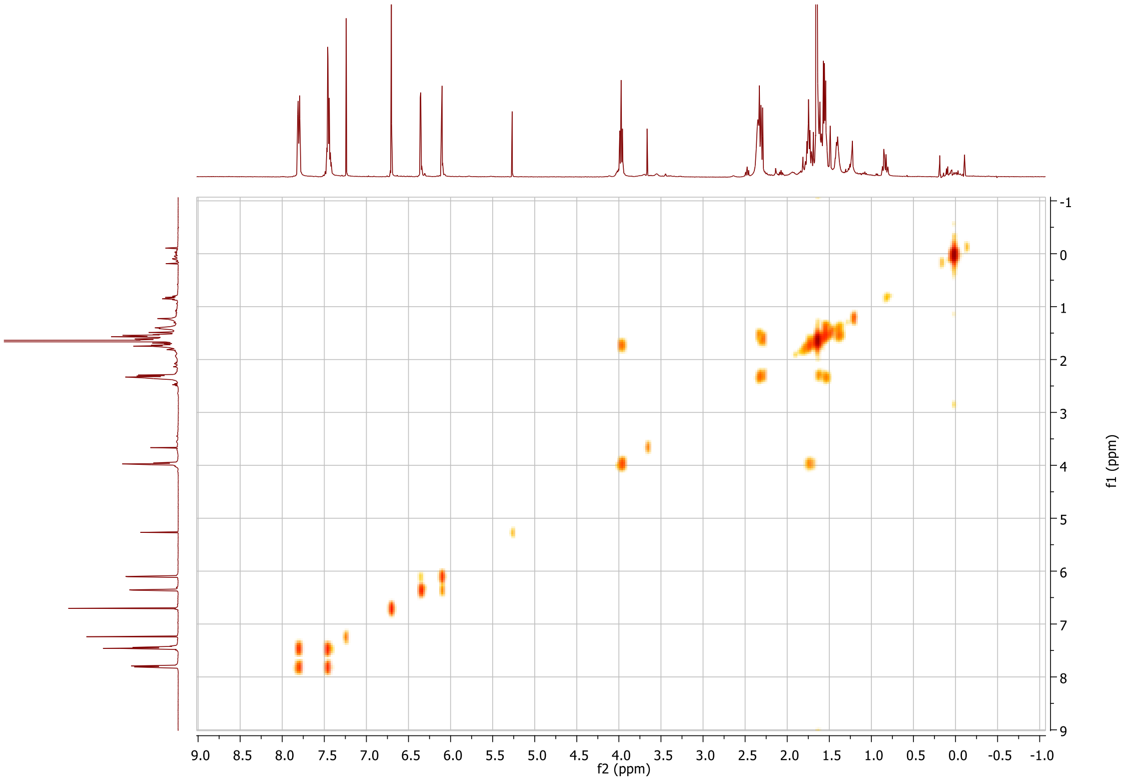

^{1}H-^{1}H COSY of **L4-Rh** in $CDCl_3$ (298 K, 400 MHz)

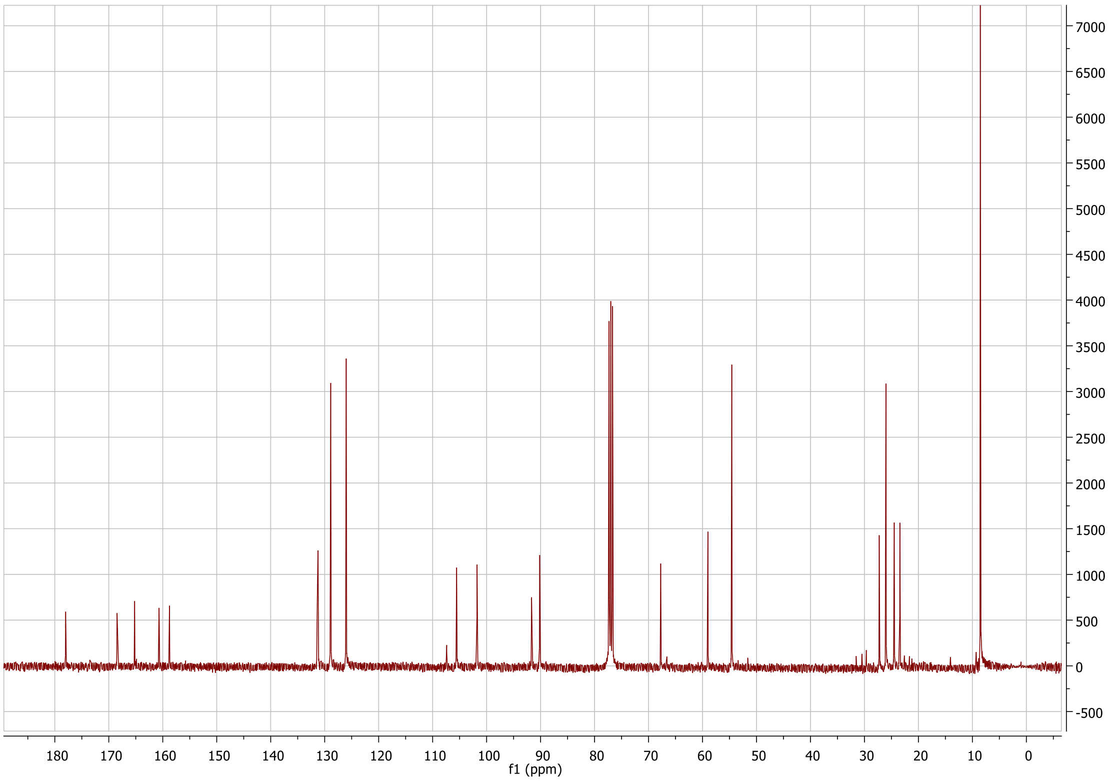

$^{13}C\{^1H\}$-NMR of **L4-Rh** in $CDCl_3$ (298 K, 101 MHz)

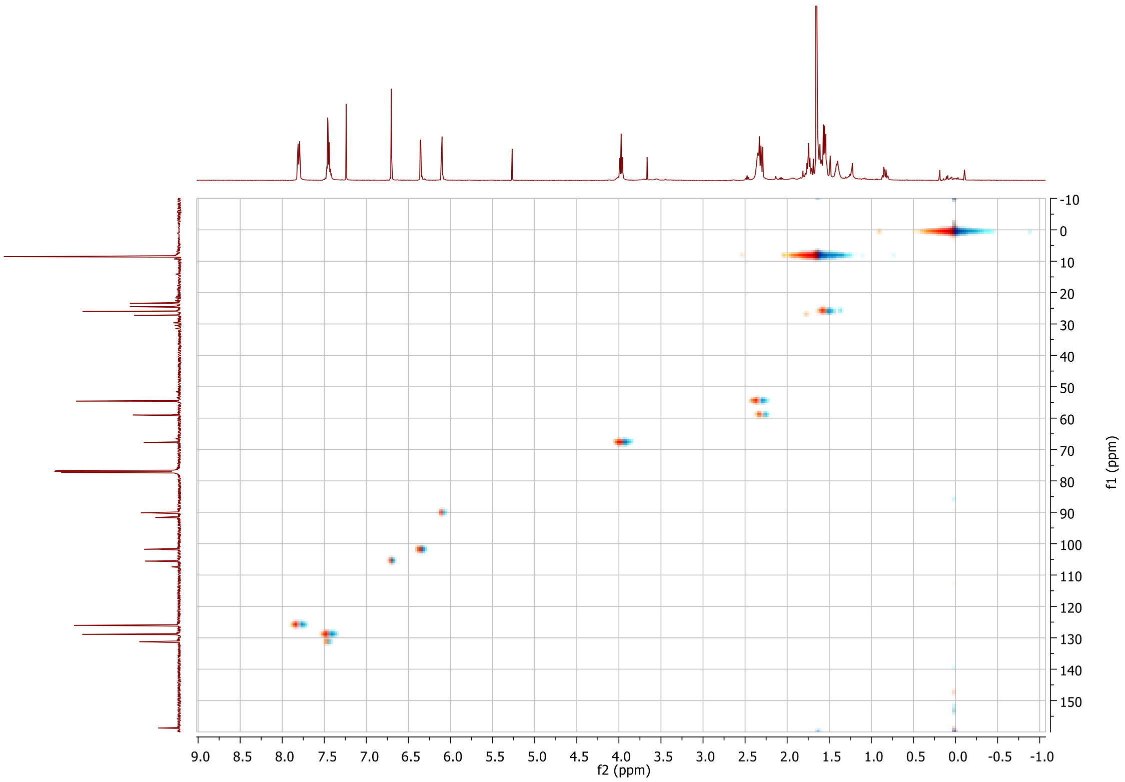

HSQC of **L4-Rh** in $CDCl_3$ (298 K, 400 MHz for ^1H)

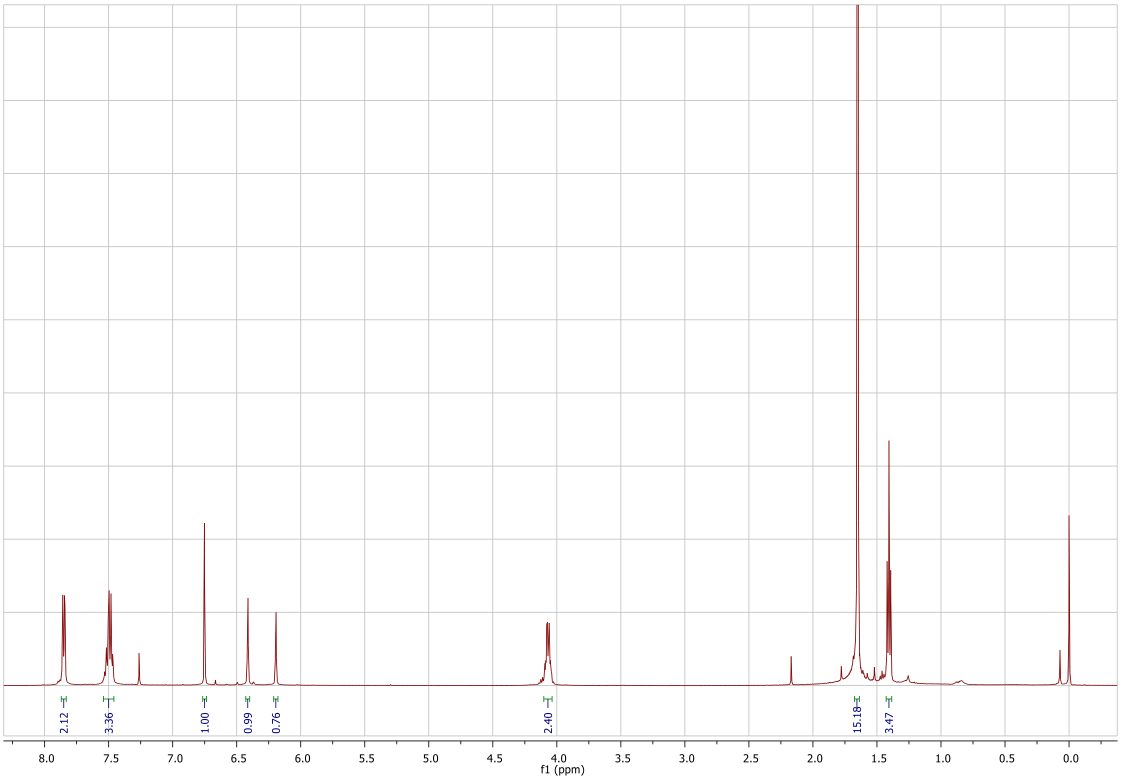

^{1}H-NMR of **L1-Ir** in $CDCl_3$ (298 K, 500 MHz)

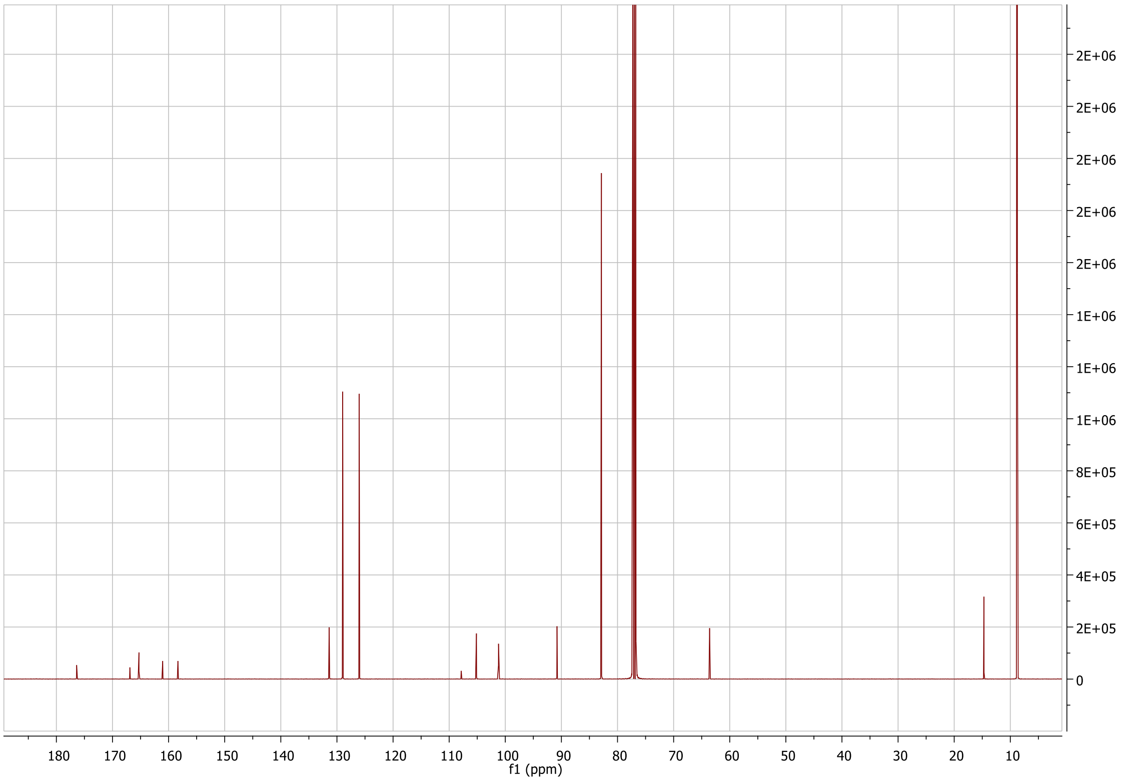

^{13}C{^{1}H}-NMR of **L1-Ir** in $CDCl_3$ (298 K, 126 MHz)

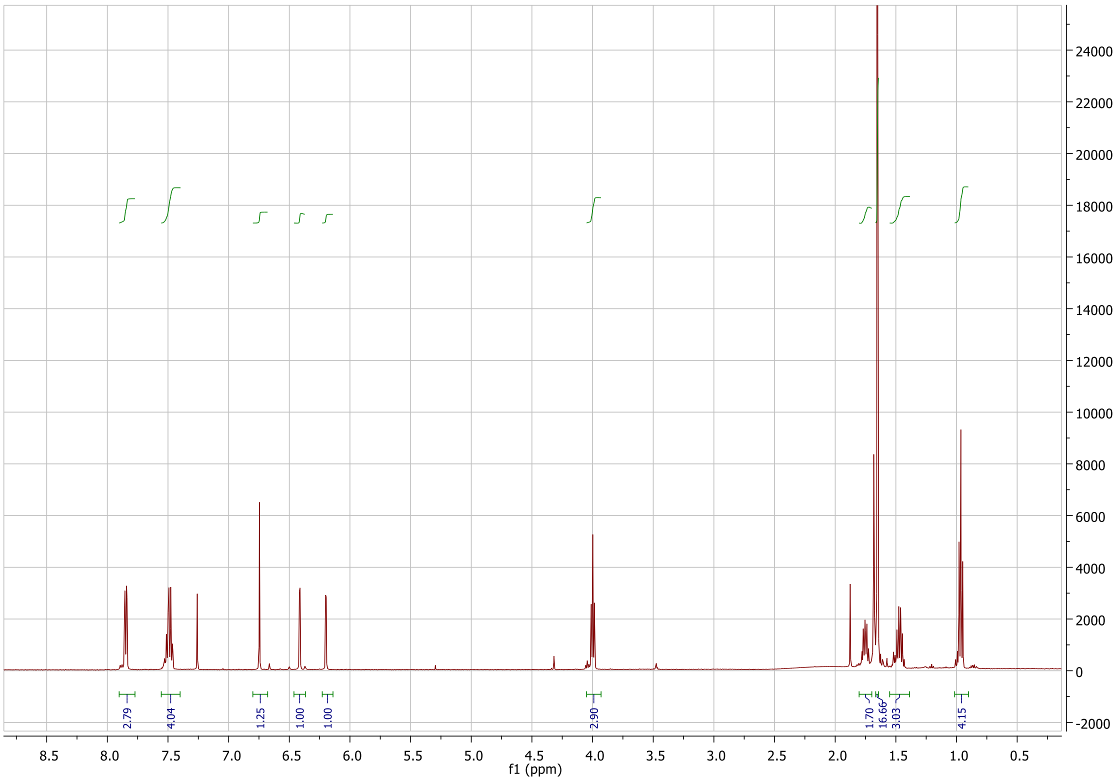

^{1}H-NMR of **L2-Ir** in $CDCl_3$ (298 K, 500 MHz)

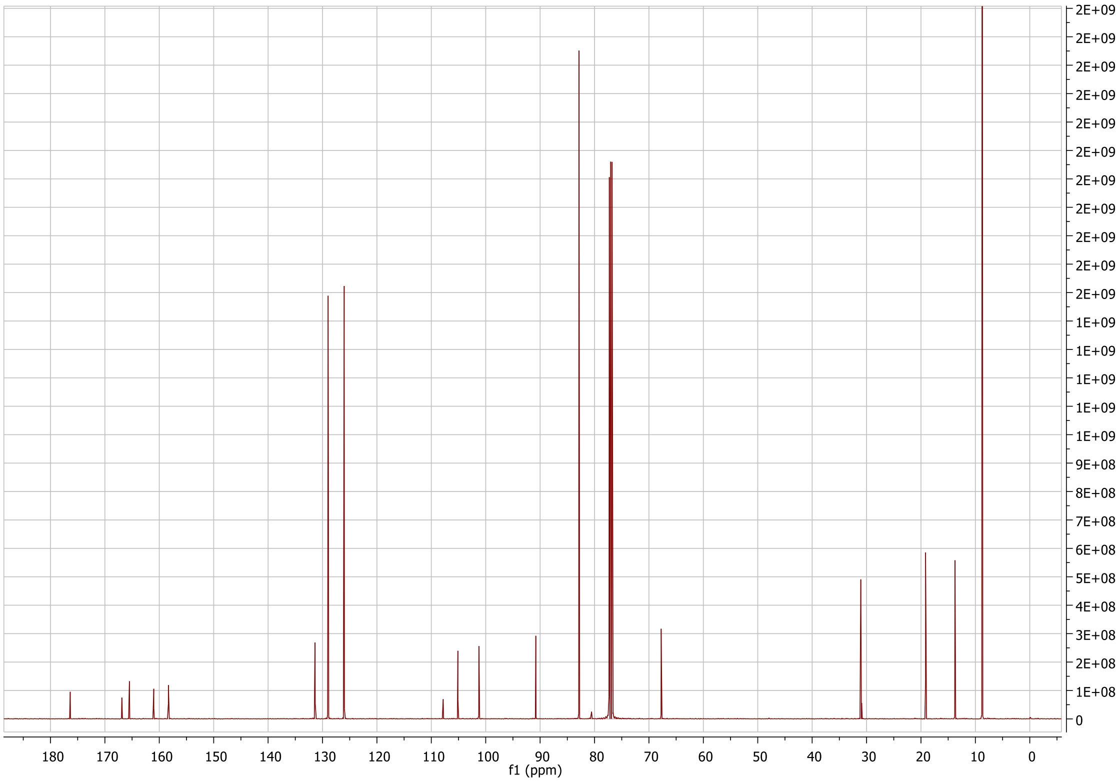

^{13}C{^{1}H}-NMR of **L2-Ir** in $CDCl_3$ (298 K, 126 MHz)

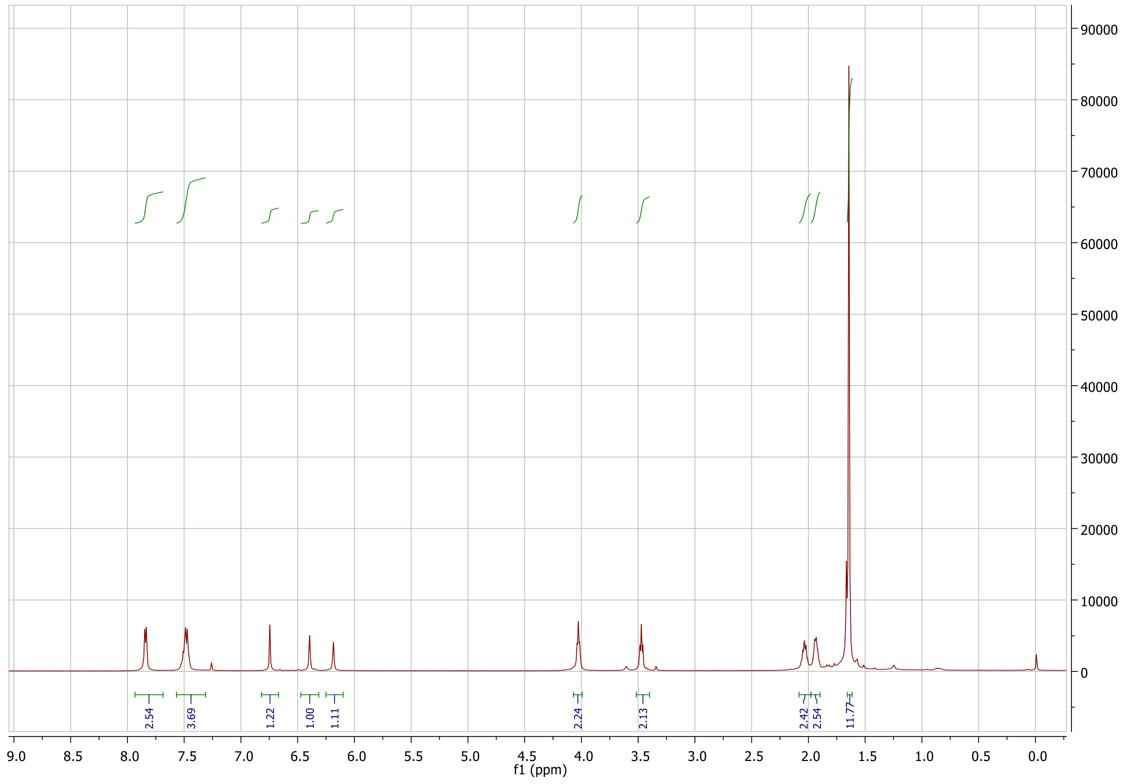

^{1}H-NMR of **L3-Ir** in $CDCl_3$ (298 K, 500 MHz)

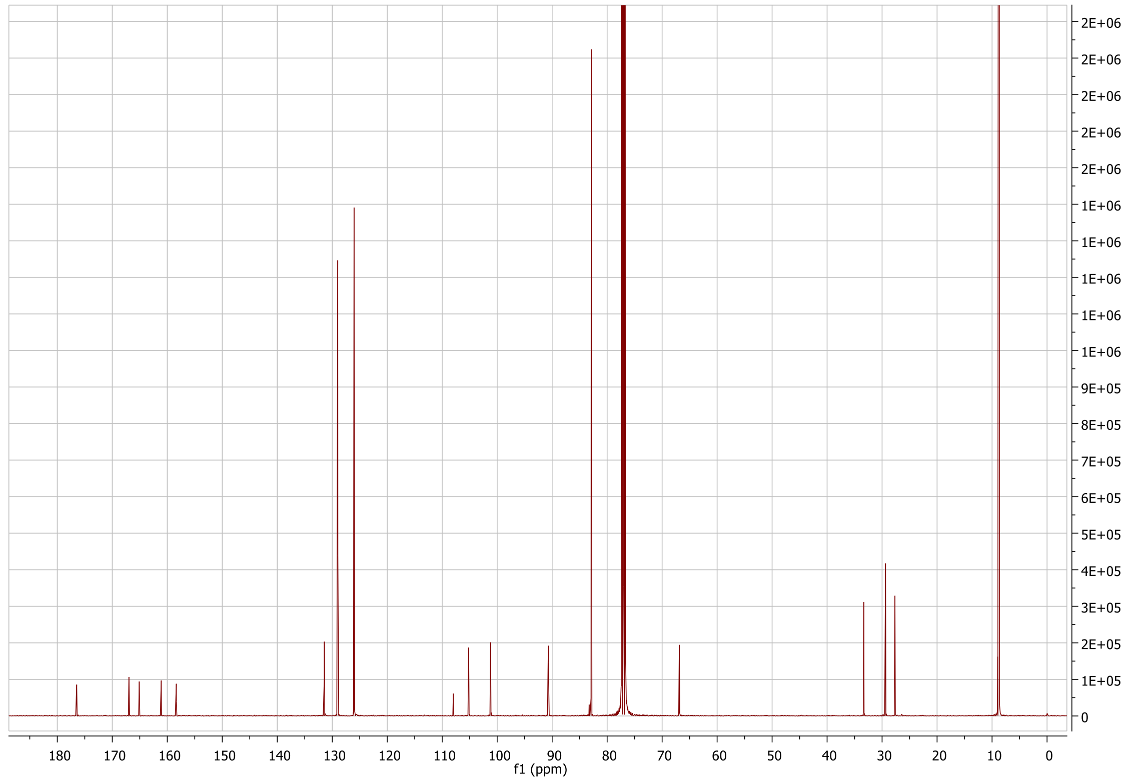

$^{13}C\{^1H\}$-NMR of **L3-Ir** in $CDCl_3$ (298 K, 126 MHz)

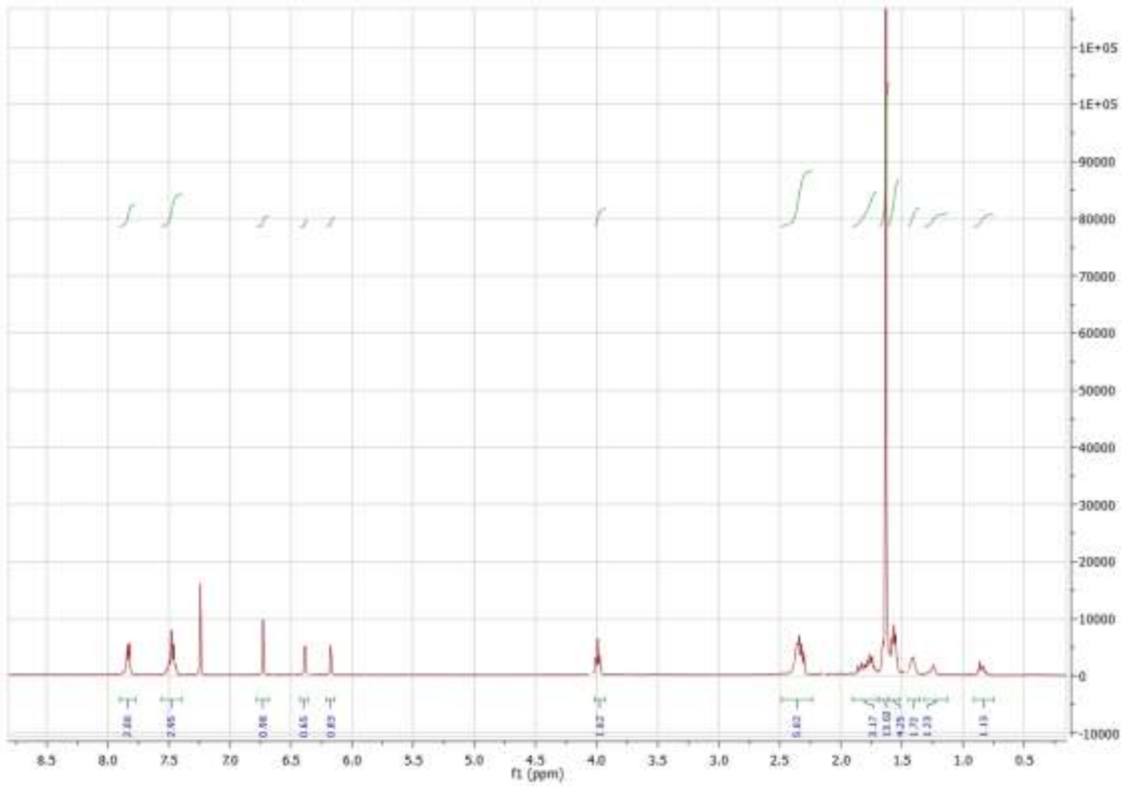

^{1}H-NMR of **L4-Ir** in CDCl3 (298 K, 400 MHz)

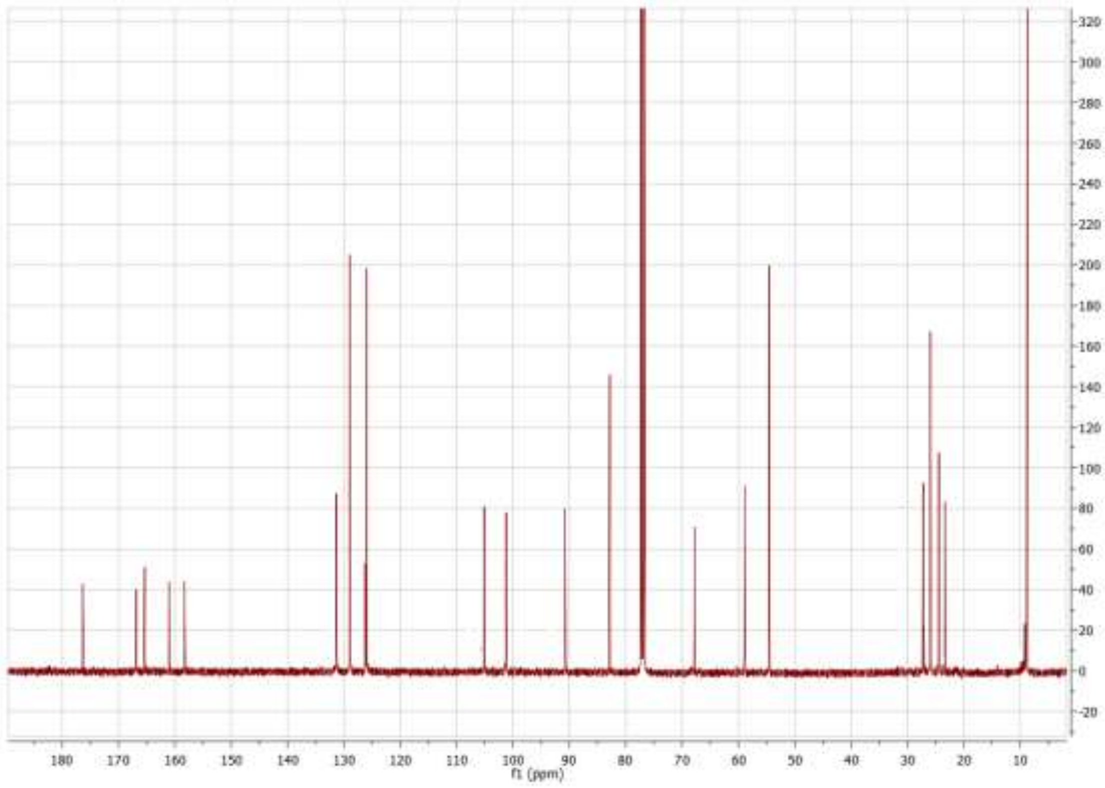

$^{13}C\{^1H\}$-NMR of **L4-Ir** in $CDCl_3$ (298 K, 101 MHz)

Table S1. ^1H NMR data for the chrysin-derived ligands in the **L-M** (L = **L1–L4** and M = Ru, Rh, Ir) complexes in $CDCl_3$.

Comp.	H^3	H^6	H^8	$H^{2'}$, $H^{6'}$	$H^{3'}$, $H^{4'}$, $H^{5'}$
**L1-Ru**	6.72	6.37	6.14	7.82	7.52-7.45
**L1-Rh**	6.74	6.40	6.14	7.84	7.52-7.45
**L1-Ir**	6.75	6.41	6.19	7.86	7.53-7.45
**L2-Ru**	6.72	6.37	6.14	7.82	7.52-7.45
**L2-Rh**	6.73	6.40	6.14	7.84	7.52-7.44
**L2-Ir**	6.75	6.41	6.20	7.85	7.53-7.45
**L3-Ru**	6.73	6.36	6.14	7.82	7.53-7.45
**L3-Rh**	6.74	6.39	6.14	7.83	7.53-7.45
**L3-Ir**	6.76	6.41	6.20	7.85	7.54-7.46
**L4-Ru**	6.72	6.36	6.14	7.82	7.52-7.46
**L4-Rh**	6.73	6.39	6.13	7.83	7.63-7.39
**L4-Ir**	6.75	6.40	6.19	7.94-7.75	7.60-7.41

Table S2. Crystal data and structure refinement for **HL2**.

Identification code	MEX_RGA55	
Empirical formula	$C_{19} H_{18} O_4$	
Formula weight	310.33	
Temperature	290(2) K	
Wavelength	0.71073 Å	
Crystal system	Monoclinic	
Space group	P 2_1/n	
Unit cell dimensions	a = 17.667(10) Å	α= 90°.
	b = 4.767(3) Å	β= 98.015(8)°.
	c = 19.306(11) Å	γ = 90°.
Volume	1610.3(17) $Å^3$	
Z	4	
Density (calculated)	1.280 Mg/m^3	
Absorption coefficient	0.089 mm^{-1}	
F(000)	656	
Crystal size	0.260 x 0.130 x 0.090 mm^3	
Theta range for data collection	2.328 to 29.600°.	
Index ranges	-23≤h≤23, -6≤k≤4, -25≤l≤26	
Reflections collected	12997	
Independent reflections	4190 [R(int) = 0.1545]	
Completeness to theta = 25.242°	99.8 %	
Absorption correction	Semi-empirical from equivalents	
Max. and min. transmission	0.746 and 0.567	
Refinement method	Full-matrix least-squares on F^2	
Data / restraints / parameters	4190 / 0 / 210	
Goodness-of-fit on F^2	0.871	
Final R indices [I>2sigma(I)]	R1 = 0.0733, wR2 = 0.1494	
R indices (all data)	R1 = 0.3157, wR2 = 0.2346	
Extinction coefficient	n/a	
Largest diff. peak and hole	0.195 and -0.219 e.$Å^{-3}$	

Table S3. Bond lengths [Å] and angles [°] for **HL2**.

C(1)-O(4)	1.354(4)
C(1)-C(2)	1.382(5)
C(1)-C(6)	1.404(6)
C(2)-C(3)	1.377(5)
C(3)-O(1)	1.345(5)
C(3)-C(4)	1.408(6)
C(4)-C(5)	1.389(5)
C(4)-C(7)	1.423(5)
C(5)-C(6)	1.368(5)
C(5)-O(3)	1.376(4)
C(7)-O(2)	1.253(5)
C(7)-C(8)	1.432(6)
C(8)-C(9)	1.336(5)
C(9)-O(3)	1.368(4)
C(9)-C(10)	1.479(5)
C(10)-C(11)	1.377(5)
C(10)-C(15)	1.385(5)
C(11)-C(12)	1.373(6)
C(12)-C(13)	1.360(6)
C(13)-C(14)	1.373(6)
C(14)-C(15)	1.374(6)
C(16)-O(4)	1.450(5)
C(16)-C(17)	1.494(5)
C(17)-C(18)	1.478(6)
C(18)-C(19)	1.486(6)
O(4)-C(1)-C(2)	124.9(4)
O(4)-C(1)-C(6)	113.7(4)
C(2)-C(1)-C(6)	121.4(4)
C(3)-C(2)-C(1)	119.1(4)
O(1)-C(3)-C(2)	118.8(4)
O(1)-C(3)-C(4)	119.6(4)
C(2)-C(3)-C(4)	121.7(4)
C(5)-C(4)-C(3)	116.6(4)
C(5)-C(4)-C(7)	121.0(4)
C(3)-C(4)-C(7)	122.4(4)
C(6)-C(5)-O(3)	116.1(4)

C(6)-C(5)-C(4)	123.9(4)
O(3)-C(5)-C(4)	120.1(3)
C(5)-C(6)-C(1)	117.3(4)
O(2)-C(7)-C(4)	122.1(4)
O(2)-C(7)-C(8)	122.6(4)
C(4)-C(7)-C(8)	115.3(4)
C(9)-C(8)-C(7)	122.4(4)
C(8)-C(9)-O(3)	121.1(4)
C(8)-C(9)-C(10)	126.7(4)
O(3)-C(9)-C(10)	112.2(4)
C(11)-C(10)-C(15)	119.4(4)
C(11)-C(10)-C(9)	120.2(4)
C(15)-C(10)-C(9)	120.5(4)
C(12)-C(11)-C(10)	119.8(5)
C(13)-C(12)-C(11)	121.2(5)
C(12)-C(13)-C(14)	119.1(5)
C(13)-C(14)-C(15)	120.8(5)
C(14)-C(15)-C(10)	119.7(4)
O(4)-C(16)-C(17)	107.2(4)
C(18)-C(17)-C(16)	113.6(4)
C(17)-C(18)-C(19)	113.9(5)
C(9)-O(3)-C(5)	120.1(3)
C(1)-O(4)-C(16)	118.1(3)

Scheme 1. dimers formed in proligand **HL2** by formation of double head-to-tail hydrogen bonds.

Table S4. Data for the hydrogen bonds formed in **HL2**.

DH···A	d(H···A) Å	d(D···A) Å	α(DHA) °	Observations
O1H1···O2	1.84	2.57	148.2	Intramolecular
C11H11···O2	2.45	3.34	160.6	Formation of dimers
C16H16B···O1	2.63	3.37	133.3	Intermolecular

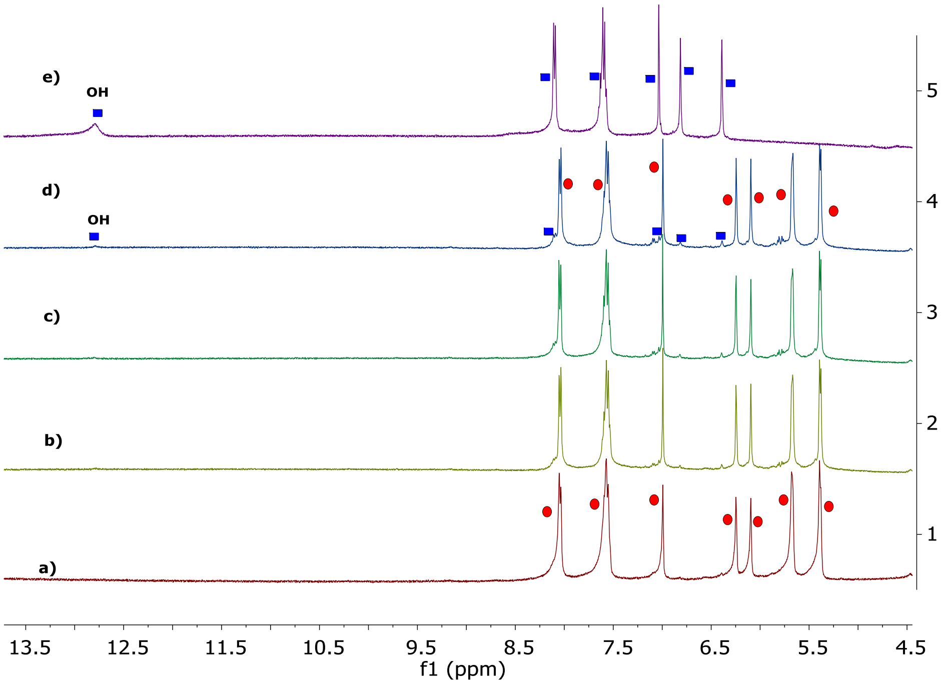

Figure S2. Aromatic area of ^{1}H NMR spectra of **L4-Ru** in DMSO-d_6 (400 MHz): a) t = 0; b) t = 3 h; c) t = 7h; d) t = 24h; e) free ligand **HL4**. Red circles: **L4-Ru**; blue squares, **HL4**.

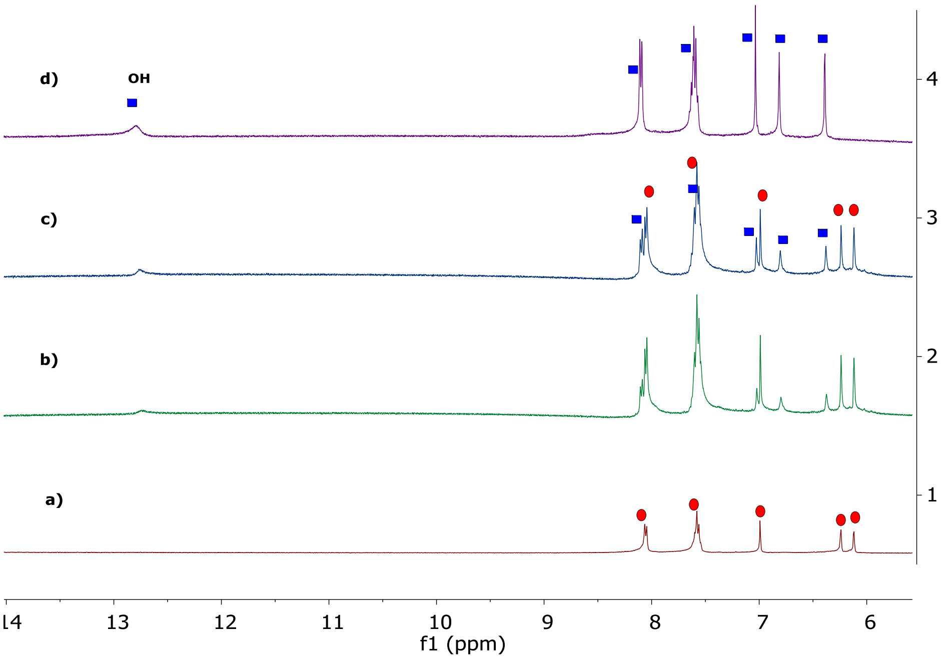

Figure S3. Aromatic area of ^{1}H NMR spectra of **L4-Rh** in DMSO-d_6 (400 MHz): a) t = 0; b) t = 7h; c) t = 24h; d) free ligand **HL4**. Red circles: **L4-Rh**; blue squares, **HL4**.

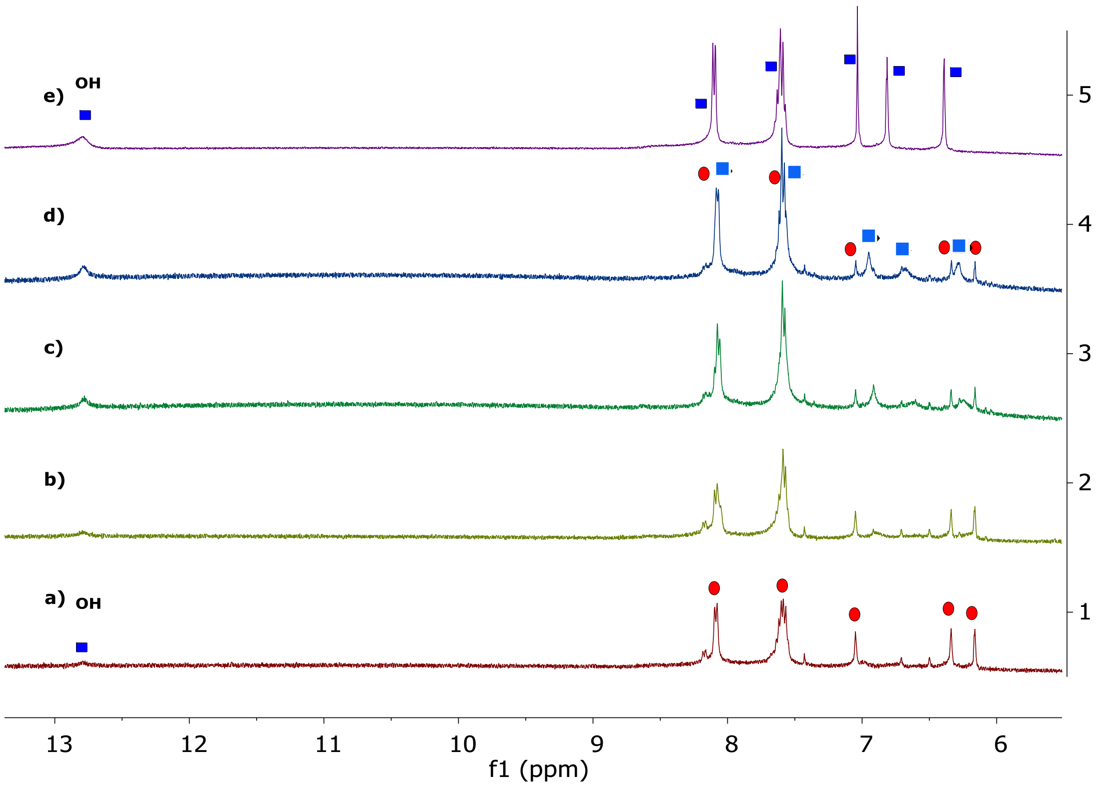

Figure S4. Aromatic area of ^{1}H NMR spectra of **L4-Ir** in DMSO-d_6 (400 MHz): a) t = 0; b) t = 30min; c) t = 7h; d) t = 24h; e) free ligand **HL4**. Red circles: **L4-Ir**; blue squares, **HL4**. In the case of d, it is proposed that the signals of **HL4** are broad because **HL4** is interacting with species of the type $[Cp^*IrZ_3]^{n+}$ (Z = H_2O or DMSO).

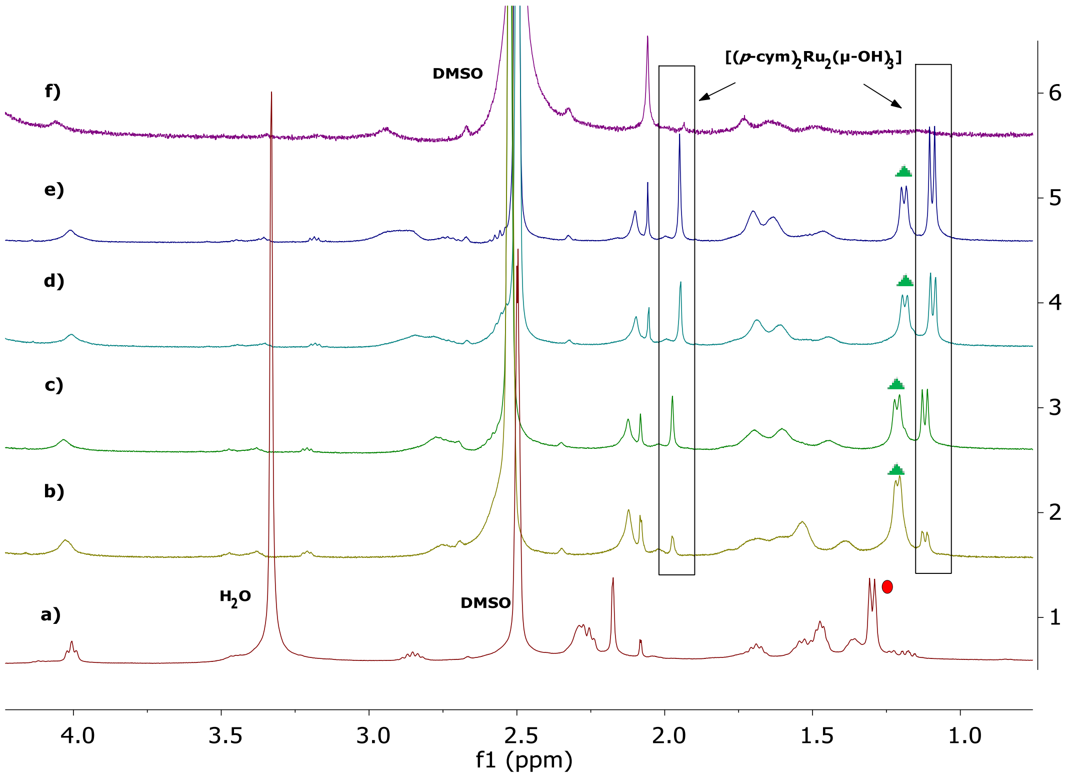

Figure S5. Aliphatic area of ^{1}H NMR spectra of **L4-Ru** in DMSO-*d*6:D2O (3:2) (400 MHz): a) **L4-Ru** after 24 h in DMSO-*d*6; b) t = 0; c) t = 3h; d) t = 7h; e) t = 24h; f) free ligand **HL4**. Red circle, **L4-Ru**; green triangles, [(p-cym)Ru(**L4**)(H_2O)]$^+$. Time values refer to the addition of D_2O.

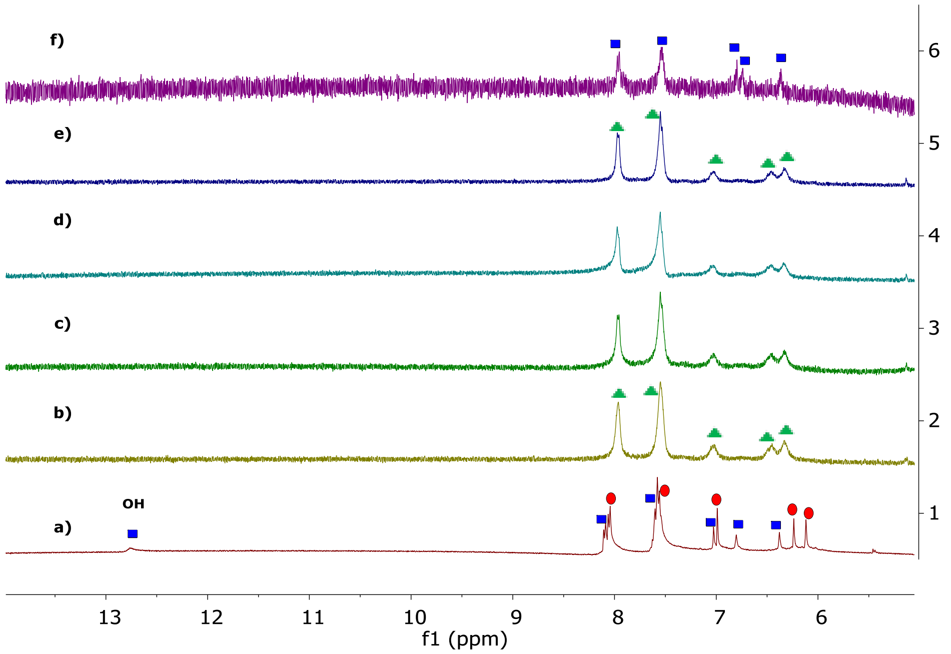

Figure S6. Aromatic area of ^{1}H NMR spectra of **L4-Rh** in DMSO-*d*6:D2O (3:2) (400 MHz): a) **L4-Rh** after 24 h in DMSO-*d*6 ;b) t = 0; c) t = 3h; d) t = 7h; e) t = 24h; f) free ligand **HL4**. Red circles: **L4-Rh**; blue squares, **HL4**; green triangles, [Cp*Rh(**L4**)(H_2O)]$^+$. Time values refer to the addition of D_2O.

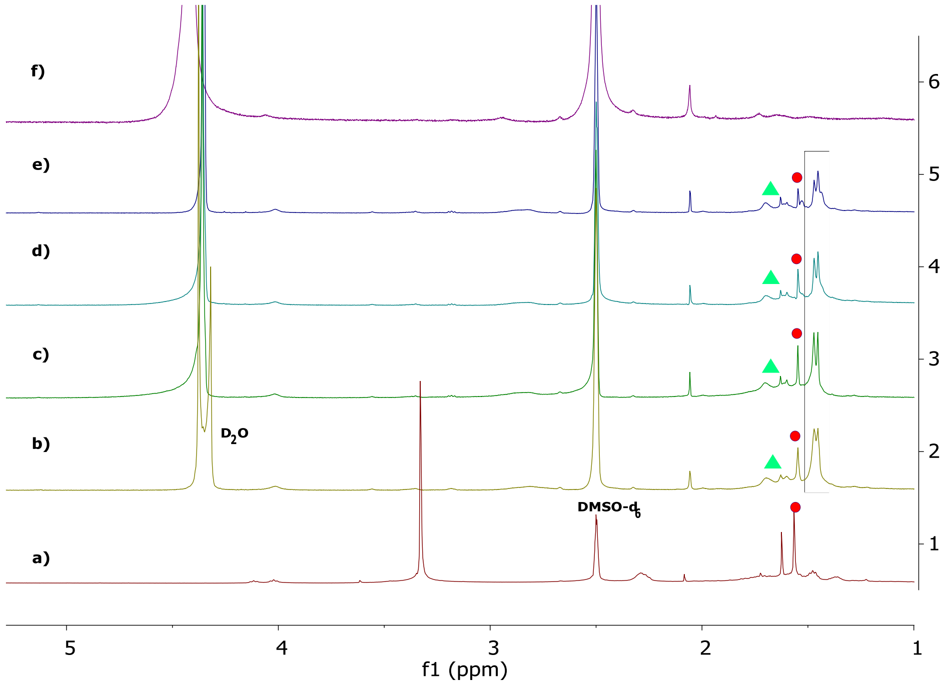

Figure S7. Aliphatic area of ^{1}H NMR spectra of **L4-Rh** in DMSO-*d*6:D2O (3:2) (400 MHz): a) **L4-Rh** after 24 h in DMSO-*d*6 ;b) t = 0; c) t = 3h; d) t = 7h; e) t = 24h; f) free ligand HL4. Red circles: **L4-Rh**; green triangles, [Cp*Rh(**L4**)(H_2O)]$^+$. The signals inside the black rectangle are proposed to be due to

$[(Cp^*Rh)_2(\mu\text{-}OH)_3]^+$ [82] or $[(Cp^*Rh)_2(\mu\text{-}OH)_2Z_2]^{n+}$ (Z = H_2O or Cl). Time values refer to the addition of D_2O.

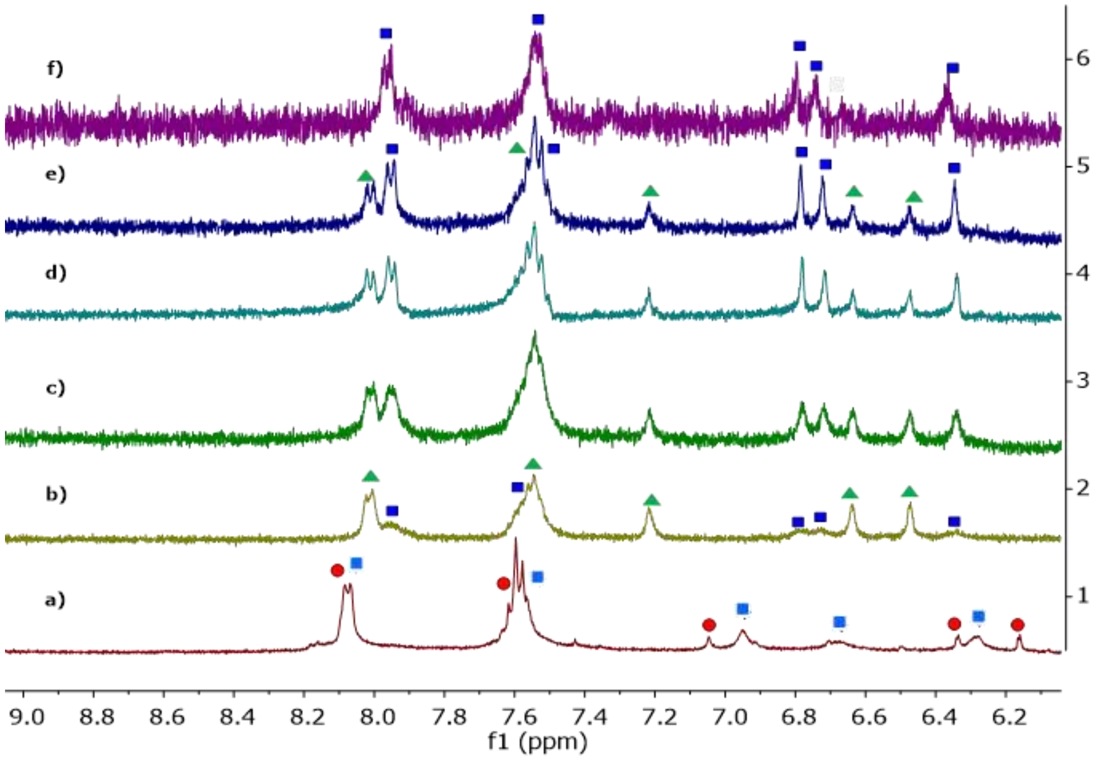

Figure S8. Aromatic area of ^{1}H NMR spectra of **L4-Ir** in DMSO-*d*$_6$:D_2O (3:2) (400 MHz): a) **L4-Ir** after 24 h in DMSO-*d*$_6$; b) t = 0; c) t = 3 h; d) t = 7h; e) t = 24h; f) free ligand **HL4**. Red circles: **L4-Ir**; blue squares, **HL4**; green triangles, [Cp*Ir(**L4**)(H_2O)]. Time values refer to the addition of D_2O.

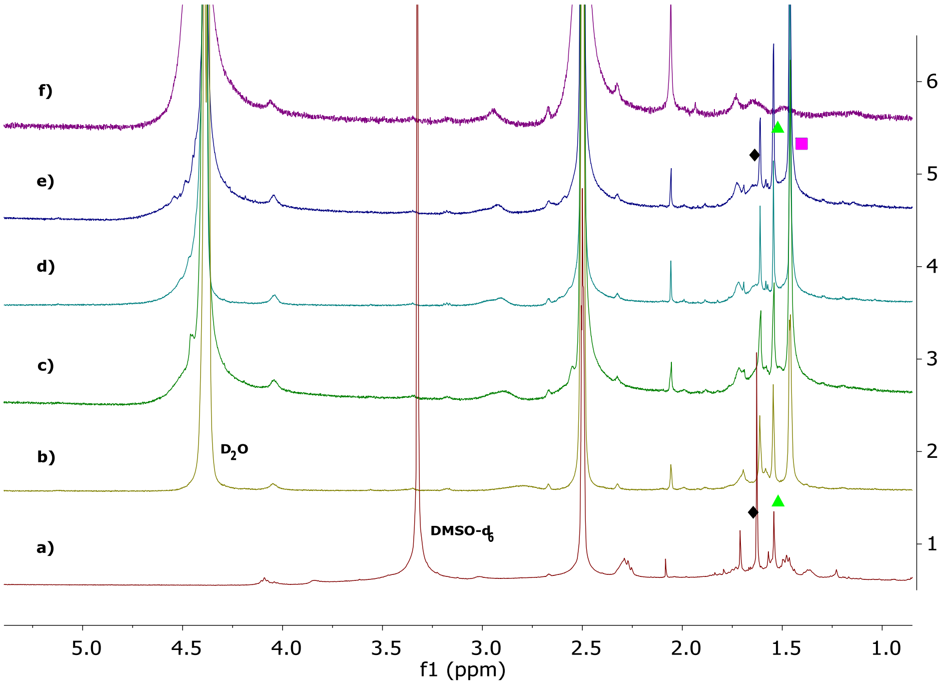

Figure S9. Aliphatic area of ^{1}H NMR spectra of **L4-Ir** in DMSO-*d*$_6$:D_2O (3:2) (400 MHz): a) **L4-Ir** after 24 h in DMSO-*d*$_6$; b) t = 0; c) t = 3 h; d) t = 7h; e) t = 24h; f) free ligand **HL4**. Green triangles, $[Cp^*Ir(\mathbf{L4})(H_2O)]^+$; black diamonds, $[Cp^*IrZ_3]^{n+}$(Z = H_2O, Cl or DMSO); pink square, $[(Cp^*Ir)_2(\mu\text{-}OH)_3]^+$. Time values refer to the addition of D_2O.

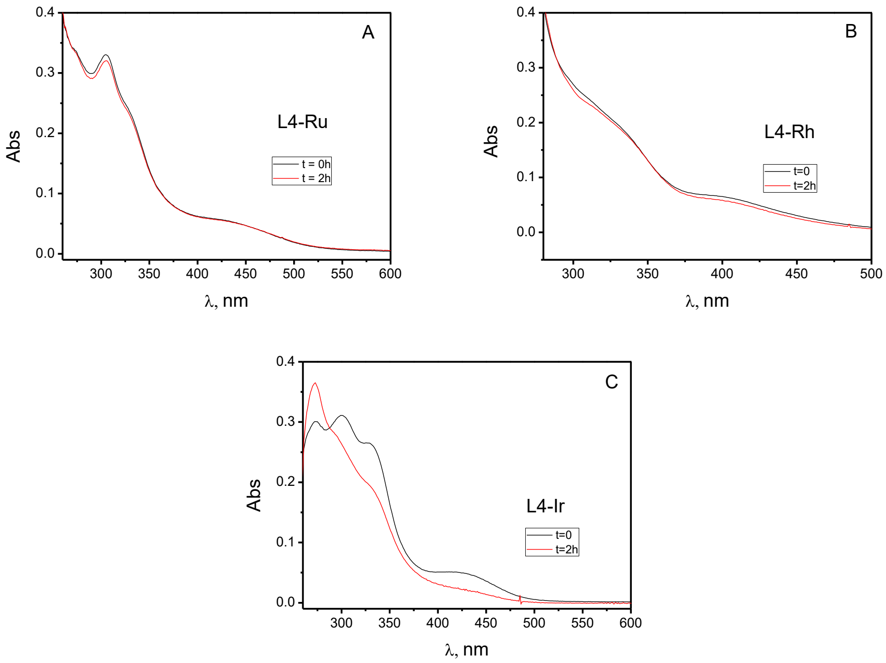

Figure S10. Absorbance spectra over time of A) **L4-Ru**, B) **L4-Rh**, C) **L4-Ir**, in DMSO. $C_D = 1.66 \times 10^{-5}$ M.

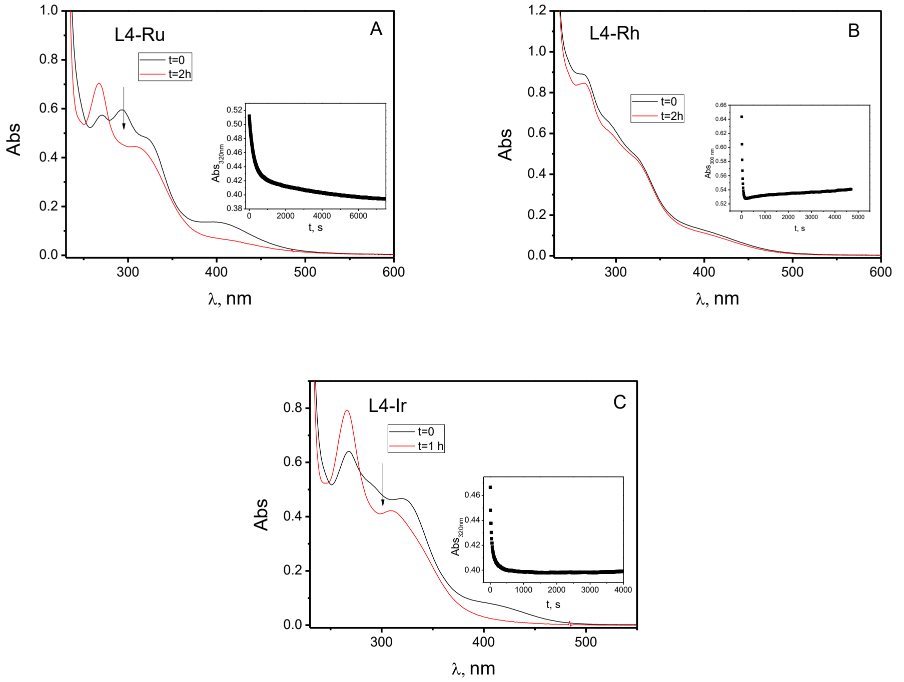

Figure S11. Absorbance spectra over time in buffer solution of A) **L4-Ru**, B) **L4-Rh** and C) **L4-Ir**. $C_D = 3.30 \times 10^{-5}$ M.

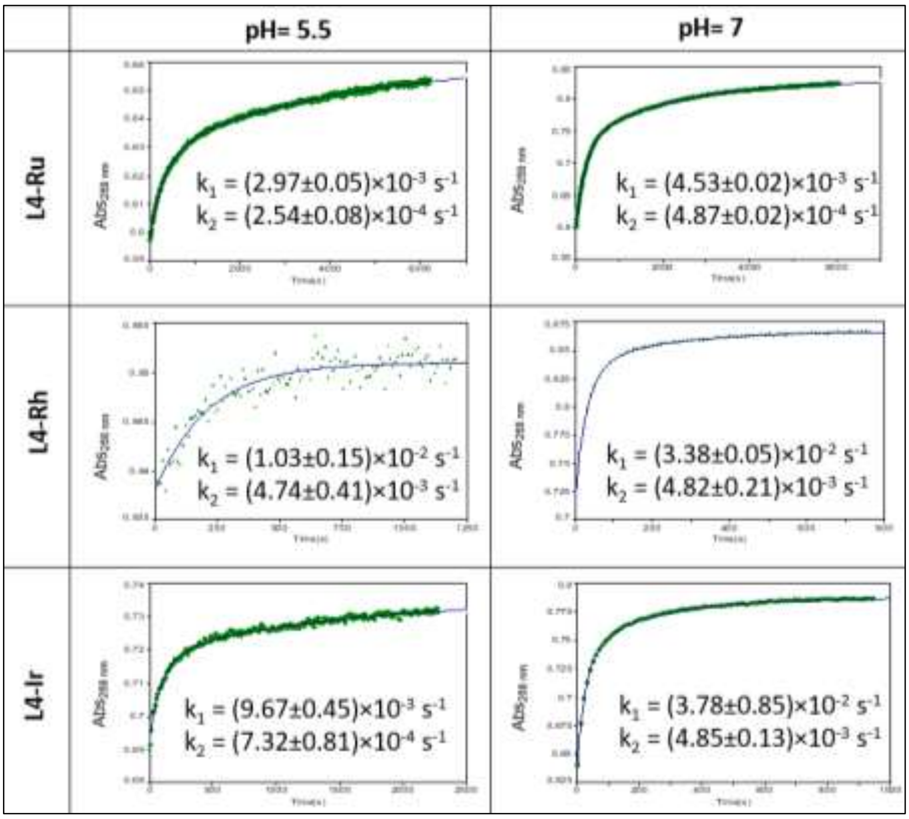

Figure S12. Kinetic traces and biexponential fitting results of **L4-Ru**, **L4-Rh** and **L4-Ir** in buffer solution at pH =5.5 and pH =7.

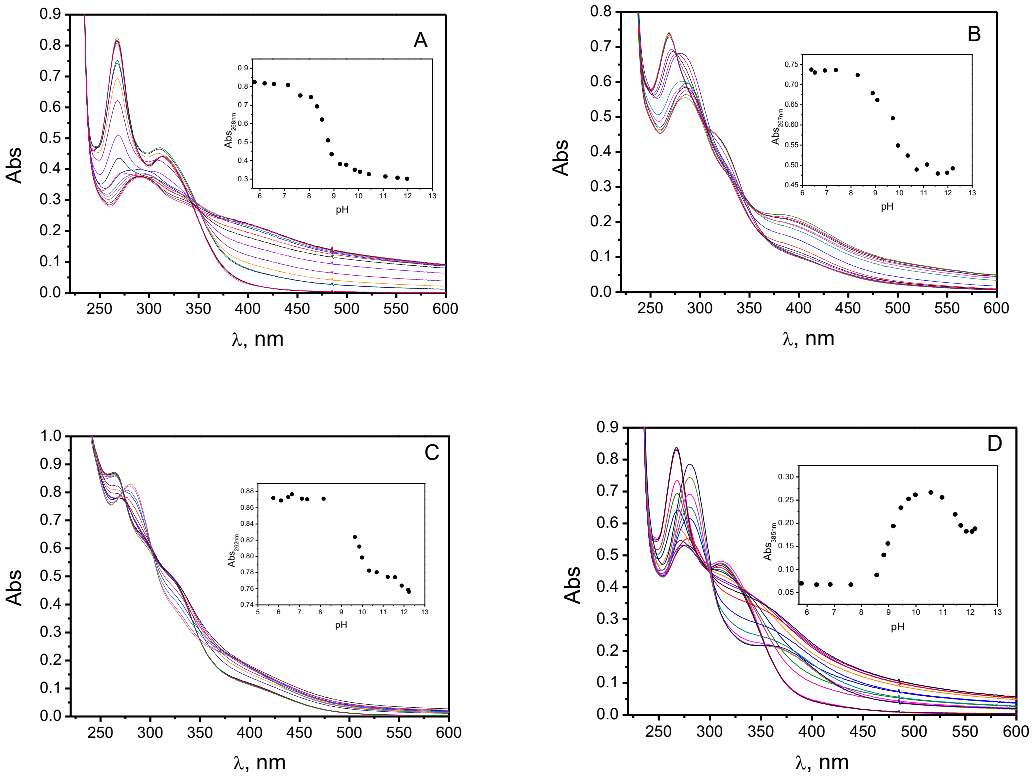

Figure S13. Absorbance spectra of A) **HL4**, B) **L4-Ru**, C) **L4-Rh**, D) **L4-Ir**, recorded in the pH 3–11 range. Inset: pH effect on the complexes absorbance at A) 268 nm, B) 267 nm, C) 262 nm, D) 385 nm. $C_D = 3.3 \times 10^{-5}$ M.

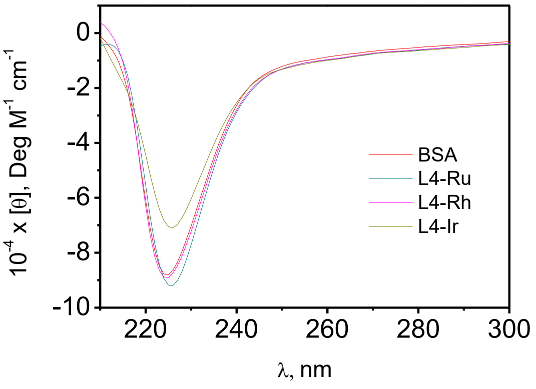

Figure S14. CD spectra of BSA in the absence and in the presence of the **L4-M** complexes (M = Ru, Rh and Ir) at a [**L4-M**]/[BSA] concentration ratio of 5. C_{BSA} = 0.5 μM, C_{DMSO} = 0.002%, I = 2.5 mM sodium cacodylate (NaCac), pH = 7.4 and T = 25 °C.
